# USP14 inhibition corrects an *in vivo* model of impaired mitophagy

**DOI:** 10.15252/emmm.201809014

**Published:** 2018-09-24

**Authors:** Joy Chakraborty, Sophia von Stockum, Elena Marchesan, Federico Caicci, Vanni Ferrari, Aleksandar Rakovic, Christine Klein, Angelo Antonini, Luigi Bubacco, Elena Ziviani

**Affiliations:** ^1^ Department of Biology University of Padova Padova Italy; ^2^ Fondazione Ospedale San Camillo IRCCS Venezia Italia; ^3^ Institute of Neurogenetics University of Lübeck Lübeck Germany; ^4^ Department of Neuroscience University of Padova Padova Italy

**Keywords:** mitochondrial membrane rupture, mitophagy, proteasome, USP14, Neuroscience, Pharmacology & Drug Discovery

## Abstract

Mitochondrial autophagy or mitophagy is a key process that allows selective sequestration and degradation of dysfunctional mitochondria to prevent excessive reactive oxygen species, and activation of cell death. Recent studies revealed that ubiquitin–proteasome complex activity and mitochondrial membrane rupture are key steps preceding mitophagy, in combination with the ubiquitination of specific outer mitochondrial membrane (OMM) proteins. The deubiquitinating enzyme ubiquitin‐specific peptidase 14 (USP14) has been shown to modulate both proteasome activity and autophagy. Here, we report that genetic and pharmacological inhibition of USP14 promotes mitophagy, which occurs in the absence of the well‐characterised mediators of mitophagy, PINK1 and Parkin. Critical to USP14‐induced mitophagy is the exposure of the LC3 receptor Prohibitin 2 by mitochondrial fragmentation and mitochondrial membrane rupture. Genetic or pharmacological inhibition of USP14 *in vivo* corrected mitochondrial dysfunction and locomotion behaviour of PINK1/Parkin mutant *Drosophila* model of Parkinson's disease, an age‐related progressive neurodegenerative disorder that is correlated with diminished mitochondrial quality control. Our study identifies a novel therapeutic target that ameliorates mitochondrial dysfunction and *in vivo* PD‐related symptoms.

## Introduction

Mitochondria generate ATP through oxidative phosphorylation at the inner mitochondrial membrane. However, they can quickly switch from transforming energy to sustain cell viability, to producing reactive oxygen species (ROS) and releasing proteins that activate cell death pathways. In order to prevent the build‐up of ROS and accumulation of aberrant proteins and organelles, cells eliminate dysfunctional mitochondria via autophagy, a process known as mitophagy that requires both autophagy machinery and the ubiquitin–proteasome complex. Key pivotal elements in the orchestration of mitophagy are proteasome localisation to depolarised mitochondria, the degradation of outer mitochondrial membrane (OMM)‐ubiquitinated proteins, and the disruption of the mitochondrial membrane (Tanaka *et al*, 2010; Chan *et al*, 2011; Yoshii *et al*, 2011). It was not clear, however, how mitochondrial membrane rupture can facilitate mitophagy until recently, when Wei *et al* (2017) demonstrated that OMM rupture leads to the exposure of Prohibitin 2, which functions as a receptor for LC3 to form mitophagic vesicles (Wei *et al*, 2017).

These findings raise the possibility that fine‐tuning UPS activity in combination with enhancing autophagy may in turn regulate mitochondrial quality control.

The ubiquitin–proteasome system (UPS) is affected in many neurodegenerative disorders and thus a key target for therapeutic intervention. One possible avenue of intervention is proteasome‐associated deubiquitinating enzymes (DUBs) which regulate proteasome activity. Among the three DUBs associated with mammalian proteasome, USP14 is an attractive target because it was demonstrated to negatively influence UPS activity by increasing protein dwelling time on the proteasome (Hanna *et al*, 2006; Peth *et al*, 2009; Lee *et al*, 2010; Kim & Goldberg, 2017) and is specifically inhibited by a small molecule IU1 (Lee *et al*, 2010) that increases proteasome activity. Interestingly, suppressing USP14 activity, either by knocking USP14 down or by IU1, leads to Beclin1‐dependent autophagy (Xu *et al*, 2016). Because of the unique capability of USP14 to enhance both proteasome complex activity and autophagy, we hypothesised that USP14 inhibition may offer additional advantages over other DUBs in promoting mitochondrial clearance. Also, the availability of the specific inhibitor IU1 makes it an ideal candidate for potential therapy development (Lee *et al*, 2010).

To monitor the therapeutic potential of USP14 inhibition in a mitophagy‐deficient background *in vivo*, we selected animal models of Parkinson's disease (PD), which are associated with mutations in the protein kinase PINK1 and E3 ubiquitin ligase Parkin, both key regulators of mitophagy (Kitada *et al*, 1998; Shimura *et al*, 2000; Valente *et al*, 2004; Silvestri *et al*, 2005; Narendra *et al*, 2008, 2010; Ziviani *et al*, 2010). Upon mitochondrial depolarisation, PINK1 anchors onto the mitochondrial membrane and recruits Parkin to ubiquitinate specific OMM proteins, which signals the elimination of dysfunctional mitochondria via autophagy (Shimura *et al*, 2000; Silvestri *et al*, 2005; Narendra *et al*, 2008, 2010; Ziviani *et al*, 2010; Chen & Dorn, 2013; Harper *et al*, 2018). Though there are other suggested pathways that may lead to the same outcome, the absence of one of them and the level of compensation may impact the efficiency of mitophagy (Baumann, 2018; Pickles *et al*, 2018). Apart from the few genes known to cause familial PD, most cases are sporadic and without any known common genetic trait. However, mitochondrial dysfunction is a key feature in both sporadic and familial PD (Parker *et al*, 1989; Schapira *et al*, 1990; Mizuno *et al*, 1998), indicating that mitochondrial quality control may hold immense therapeutic implications in this disorder.

Here, we report that pharmacological or genetic inhibition of USP14 leads to increased mitochondrial clearance. Mitochondrial fragmentation and mitochondrial membrane rupture to expose the LC3 autophagy receptor Prohibitin 2 are key elements for USP14‐induced mitophagy. *In vivo*, USP14 inhibition corrected mitochondrial dysfunction and locomotion impairment in the established PINK1 and Parkin mutant *Drosophila* model of neurodegeneration, highlighting the potential of USP14 inhibitors as therapeutics for PD symptoms.

## Results

### USP14 inhibition/knockdown induces mitophagy

**Figure 1:**
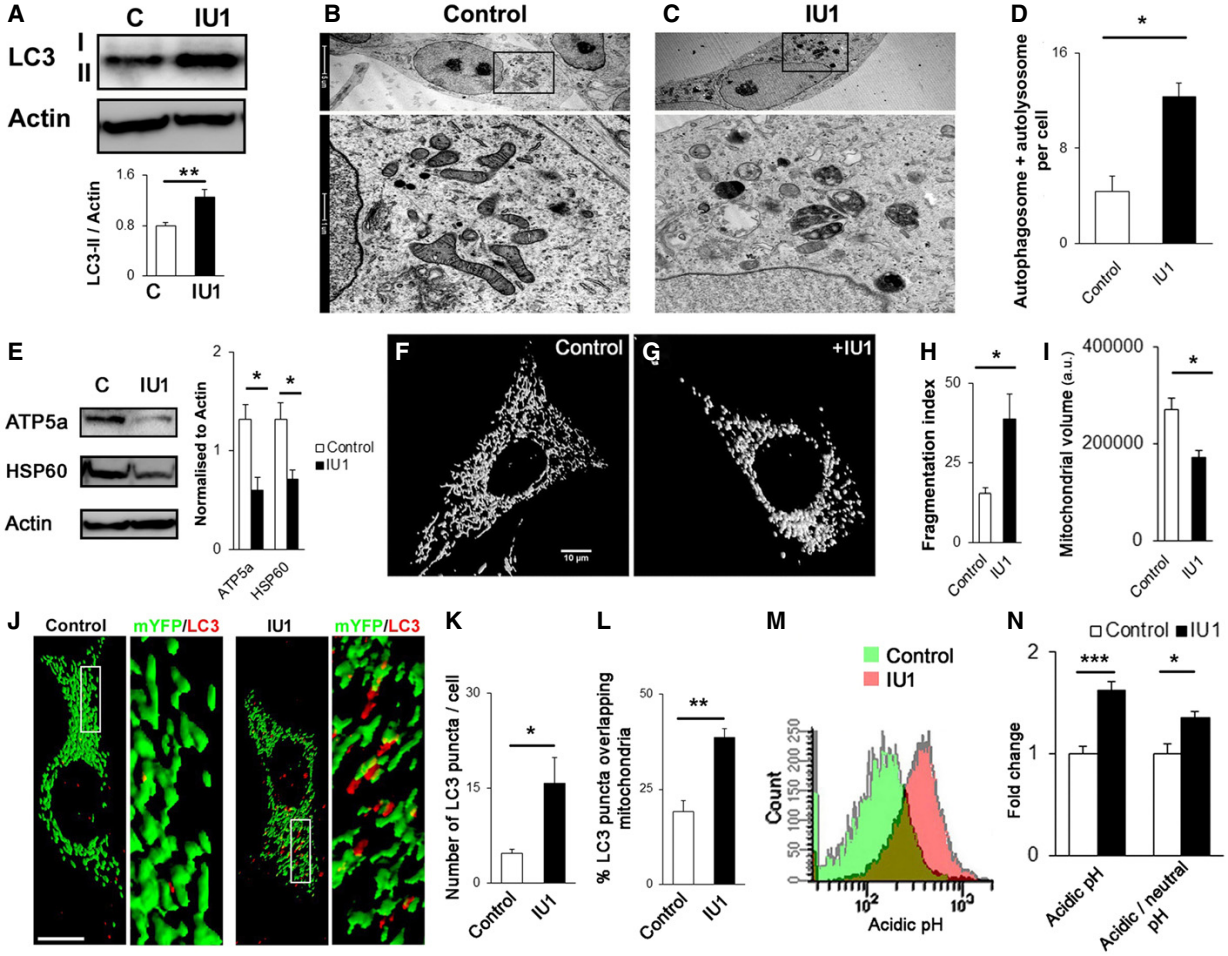
Treatment with the USP14 inhibitor IU1 induces mitophagy in SH‐SY5Y cells AWestern blot analysis of LC3 in SH‐SY5Y cells after IU1 treatment (100 μM, 24 h). Graphs represent the mean ± SEM. *N* = 8.B–DElectron microscopy images were taken to quantify the number of autophagosomes and autolysosomes present in control (DMSO) or IU1‐treated cells. At least 55 cells were analysed from three different experiments. Graphs represent the mean ± SEM.EWestern blot analysis of mitochondrial marker proteins ATP5a and HSP60, normalised by actin after 24 h of DMSO (C) or IU1 (100 μM) treatment. *N* = 3. Graphs represent the mean ± SEM.F–IConfocal images of cells transfected with mito‐YFP and treated with DMSO (F, control) or IU1 (G) for 24 h. Fragmentation index (H) and total mitochondrial volume/cell were measured and are represented in (I) as mean ± SEM. At least 35 cells were analysed from three different experimental conditions. Scale bar: 10 μm.J–LMito‐YFP‐transfected cells were treated with IU1 (100 μM, 24 h), fixed and immunolabelled for LC3. The number of LC3 dots/cell (K) and LC3 dots overlapping mitochondria (L) was counted and represented as mean ± SEM. At least 35 cells from three independent experiments were analysed. Scale bar: 10 μm.M, NCells were transfected with mito‐Keima and treated with DMSO or IU1 for 24 h and subjected to FACS analysis, counting 10,000 cells per experiment. (M) represents the shift of the signal intensity in IU1‐treated cells when excited at 560 nm (false colour). (N) represents the average intensity of the mito‐Keima protein when excited at 560 nm and the ratio between 560:405 nm. Graphs represent the mean ± SEM from three different sets of experiments.Data information: Student's *t*‐test. *P ≤ 0.05, **P ≤ 0.01, *** *P* ≤ 0.001.Source data are available online for this figure.

To standardise a dose of USP14 inhibitor IU1, we incubated SH‐SY5Y cells with different concentrations (1–500 μM) for 24 h and assessed cell viability by MTT assay. We found that doses > 200 μM reduce cell viability, whereas doses up to 100 μM have no effect on cell viability (Appendix Fig S1A). To reconfirm the MTT assay results, we stained the cells with propidium iodide and Hoechst (Appendix Fig S1B). We found that 100 μM does not cause cell death. The number of apoptotic cells in the 200 μM dose was highly variable, but 500 μM IU1 caused severe cell death. To further confirm that IU1 does not affect neuronal survivability at similar doses *in vivo*,* Drosophila* expressing neuronal GFP (UAS CD8‐GFP, elavC155‐Gal4) were treated with different concentrations of IU1 (1–100 μM, 3 days, mixed with food; Appendix Fig S1C). The treatment did not cause any adverse effects on the gross morphology of wing motor neurons of *Drosophila*. To ensure IU1 is effective in human and mouse cells (the two main cell types used for the study) and increases proteasome activity, we used a substrate that is dependent on ubiquitin and UPS. We transfected cells with Ub‐R‐GFP, a proteasomal substrate that is maintained at low levels in normal conditions (Dantuma *et al*, 2000), and then treated the cells with 100 μM IU1 for 24 h. We found a clear decrease in the Ub‐R‐GFP protein levels after IU1 treatment. This effect was reversed by co‐incubation with the UPS inhibitor MG132 (Appendix Fig S2A). Before assessing mitophagy with this concentration of IU1, we wanted to confirm that USP14 inhibition can increase autophagy in SH‐SY5Y cells, as previously suggested (Xu *et al*, 2016). In accordance with the study by Xu *et al* (2016), we found an increase in LC3‐II protein levels after 24 h of IU1 (100 μM) treatment (Fig 1A) or USP14 siRNA treatment (Appendix Fig S3A). We also found an increase in the LC3‐II levels after IU1 treatment when cells were co‐incubated with chloroquine or NH_4_Cl (Appendix Fig S2B). Furthermore, electron microscopy analysis of SH‐SY5Y cells revealed a high number of autophagic vesicles after 24 h of IU1 (100 μM) treatment (Fig 1B–D). An increased number of autophagosomes and autolysosomes was also detected when USP14 was knocked down by siRNA (Appendix Fig S3B and C), thus confirming the specificity of IU1. To characterise the autophagic vacuoles further, we counted the number of initial (AVi) and degradative/mature autophagic vacuoles (Klionsky *et al*, 2016). We found a clear increase in the number of total and degradative autophagic vacuoles (Appendix Fig S2C).

In order to evaluate mitochondrial content in SH‐SY5Y cells, we next measured total mitochondrial volume as well as the levels of the mitochondrial inner membrane‐ and matrix‐resident proteins, ATP5a and HSP60, respectively. Potential enhancement of mitophagy should be reflected by a decrease in these two parameters. We monitored the levels of ATP5a and HSP60 by immunoblot and found that USP14 inhibition by IU1 (Fig 1E) or its knockdown (Appendix Fig S3D) resulted in a significant decrease when compared to the respective control group. Analysis of mitochondrial shape (elongated/fragmented) and mitochondrial volume of SH‐SY5Y cells expressing mito‐YFP revealed increased mitochondrial fragmentation and decreased mitochondrial volume when USP14 was inhibited (Fig 1F–I) or knocked down (Appendix Fig S3E–G). Next, we transfected cells with mito‐YFP and immunostained them for LC3. IU1‐treated cells showed increased LC3 puncta formation as well as increased overlap with mitochondria‐like structures (Fig 1J–L). USP14 knockdown also showed similar results when the mitochondrial marker ATP5a and the LC3 protein were immunostained, and co‐localisation was measured (Appendix Fig S3H–K). An increased number of autophagic vesicles with a mitochondria‐like structure were confirmed by electron microscopy analysis in both IU1‐ and USP14 siRNA‐treated groups (Appendix Fig S4A and B, respectively). Next, we verified the hypothesis that IU1‐mediated mitochondrial loss requires the autophagic machinery. Because NH_4_Cl treatment along with IU1 showed an increase in LC3‐II levels (Appendix Fig S2B), which was also depicted earlier by Xu *et al* (2016), we co‐incubated cells with IU1 and NH_4_Cl (Appendix Fig S5A) and probed them for HSP60/ATP5a. We could not find any significant decrease in these mitochondrial markers after IU1 treatment when NH_4_Cl was co‐administered (Appendix Fig S5A). To further support our hypothesis by using a genetic approach, we incubated ATG7 WT/KO MEF cells with IU1. While a decrease in both ATP5a and HSP60 protein levels was evident in ATG7 WT cells, there was no significant decrease in the ATG7 KO cells (Appendix Fig S5B). To further confirm enhanced mitophagy in USP14‐inhibited cells, we transfected SH‐SY5Y cells with mito‐Keima, which has different excitation spectra at neutral (405/615 nm) and acidic pH (561/615 nm). IU1‐treated cells showed a clear shift in spectra (Fig 1M) with a significant increase in the average signal intensity at 561 nm (i.e. acidic pH) (Fig 1N). Together, these results support that USP14 pharmacological or genetic inhibition induces mitophagy.

### USP14‐mediated mitophagy is DRP1 and Mfn2 dependent

**Figure 2:**
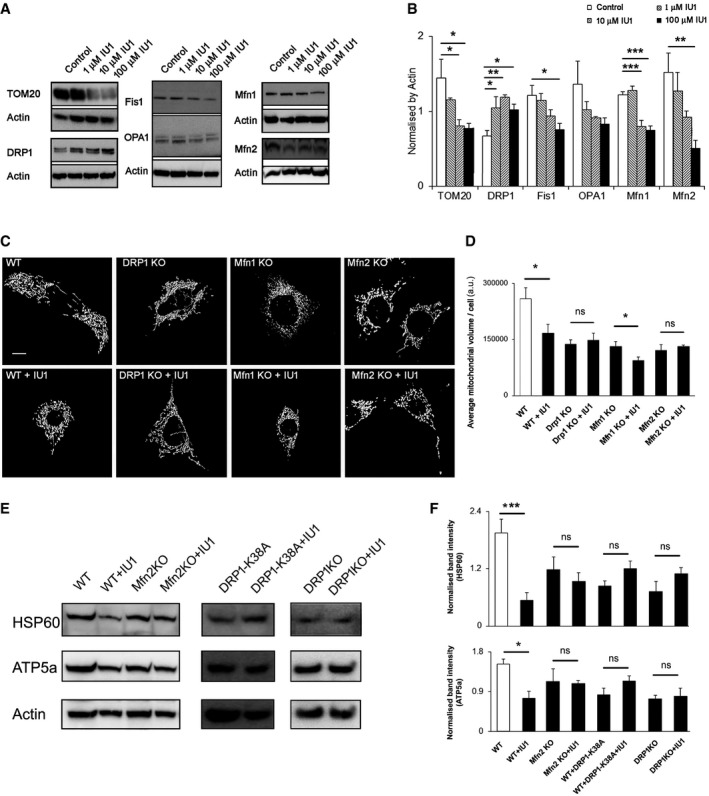
IU1‐facilitated mitochondrial clearance is DRP1 and Mfn2 dependent A, BWestern blot analysis of the indicated proteins in cell lysates from SH‐SY5Y cells, treated with different concentrations of IU1 (1–100 μM) for 24 h. Bar graphs represent the mean ± SEM. Blots are representative of three independent experiments. ANOVA followed by Dunnett's test.C, DRepresentative images of MEF cells knocked out for the indicated mitochondrial dynamics proteins and transfected with mito‐YFP (C) with/without IU1 treatment (100 μM, 48 h). Scale bar: 10 μm. (D) Mitochondrial volume/cell was measured as described and is represented as mean ± SEM. At least 35 cells were evaluated. Student's *t*‐test.E, FWestern blot analysis of HSP60 and ATP5a as mitochondrial markers in the indicated knockout MEF cells (with/without the indicated DRP1 variant) after 48 h of IU1 treatment. Bar graphs represent the mean ± SEM; *n* = at least 3. Student's *t*‐test.Data information: *P ≤ 0.05, **P ≤ 0.01, ***P ≤ 0.001.Source data are available online for this figure.

Because mitochondrial shape and size can affect mitophagy (fragmented ones are preferred over the elongated ones) (Twig *et al*, 2008; Gomes *et al*, 2011), we next evaluated the levels of the mitochondria‐shaping proteins DRP1, Fis1, Mfn1, Mfn2 and OPA1 in USP14‐inhibited cells. SH‐SY5Y cells treated with IU1 (1–100 μM) for 24 h showed a dose‐dependent decrease in the level of TOM20, reflecting the decrease in mitochondrial content (Fig 2A and B). The levels of the mitochondrial pro‐fission protein DRP1 were increased after 24 h, whereas the levels of Fis1 and the mitochondrial fusion proteins Mfn1 and Mfn2 were reduced in a dose‐dependent manner (Fig 2A and B). There was only a trend towards a decrease in the level of the mitochondrial pro‐fusion protein OPA1 after IU1 treatment (Fig 2A and B). USP14 knockdown by siRNA also showed similar results after 24 h, except for OPA1, which was also significantly decreased (Appendix Fig S6A and B). A further follow‐up after 72 h of USP14 knockdown showed decreased levels of all the dynamics‐related proteins, except for Fis1, which could be due to further reduction of mitochondrial content (Appendix Fig S6C and D). These results indicated a shift towards mitochondrial fission at 24 h, consistent with mitochondrial fragmentation prior to mitophagy. To confirm that this shift is essential for USP14‐mediated mitophagy, we incubated MEF cells genetically ablated for DRP1/Mfn1/Mfn2 or WT MEF cells with 100 μM IU1 for 48 h and measured mitochondrial volume by confocal imaging as well as the levels of the mitochondrial inner membrane‐ and matrix‐resident proteins, ATP5a and HSP60, by Western blot. IU1‐mediated mitochondrial volume loss was abolished in DRP1 and Mfn2 KO cells, while the effect persisted in the WT and Mfn1 KO cells (Fig 2C and D). Measurement of HSP60 and ATP5a protein levels by Western blot also confirmed DRP1 and Mfn2 dependency for mitochondrial clearance by IU1 (Fig 2E and F). Transfection of wild‐type MEF cells with DRP1‐K38A (a dominant negative form of DRP1) also abolished the effect of IU1, which was reflected on the HSP60 and ATP5a protein levels (Fig 2E and F). Thus, USP14‐mediated mitophagy requires DRP1 and Mfn2.

### USP14‐induced mitochondrial clearance is PINK1/Parkin independent

**Figure 3:**
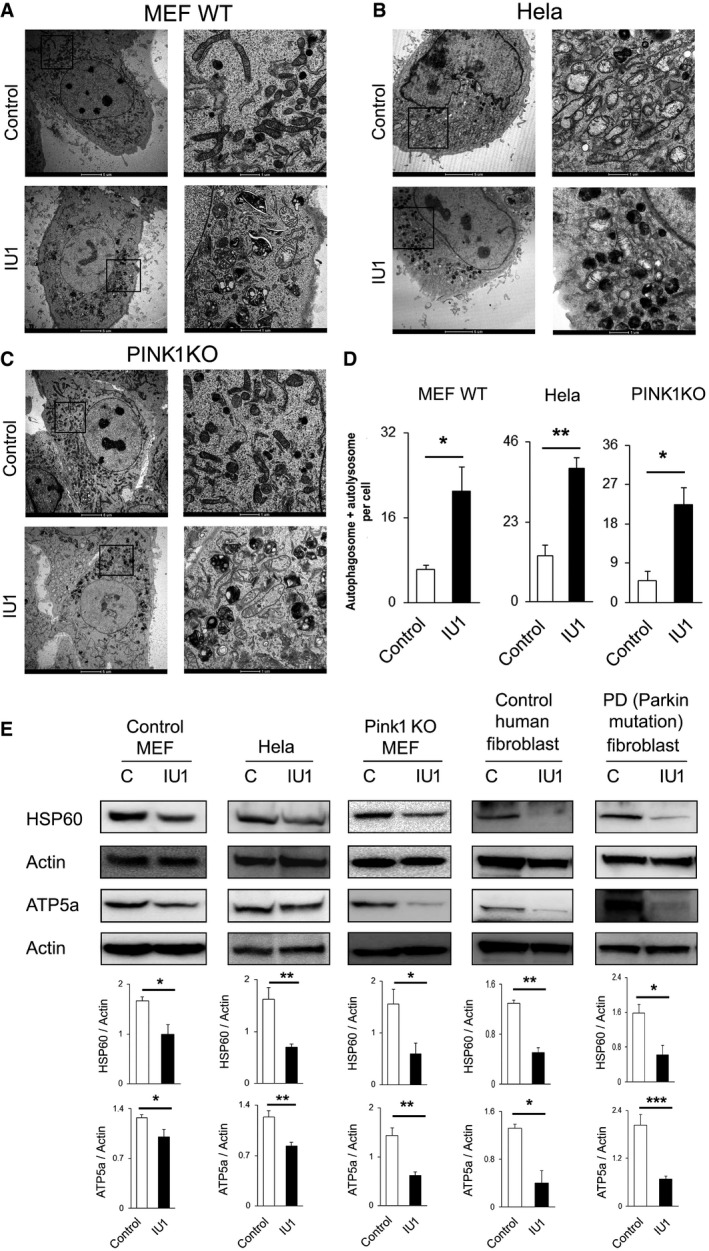
IU1 treatment‐mediated mitophagy is Parkin/PINK1 independent A–DWild‐type MEF (A), HeLa (B) and PINK1 KO MEF (C) cells were treated with IU1 (48 h, 100 μM), and electron microscopy images were evaluated for autophagic vesicle formation. (D) Bar graphs represent the mean ± SEM of the number of autophagosomes and autolysosomes per cell from three different sets of experiments; at least 40 cells were analysed from each group. Student's *t*‐test.EWestern blot analysis of HSP60 protein with or without IU1 treatment in the indicated cell type. Bar graphs represent the mean ± SEM. *N* = at least three independent experiments. Student's *t*‐test.Data information: *P ≤ 0.05, **P ≤ 0.01, ***P ≤ 0.001.Source data are available online for this figure.

We next evaluated whether USP14‐mediated mitophagy is dependent on the canonical PINK1/Parkin mitophagy pathway. To assess PINK1 dependency, we used PINK1 KO MEF cells, and to evaluate Parkin dependency, we used HeLa cells and Parkin mutant human fibroblasts derived from skin biopsies of human patients with compound heterozygous (R275W and exon 3 deletion) *parkin* mutations (see Materials and Methods for clinical details). Wild‐type MEF cells and HeLa cells, which express low and undetectable amounts of Parkin, respectively, and PINK1 KO MEF cells were treated with 100 μM IU1 for 48 h and analysed by electron microscopy. Increased autophagosome and/or autolysosome formation was detected in all three cell types (Fig 3A–D), as well as decreases in HSP60 and ATP5a protein levels (Fig 3E). Human control fibroblast and *Parkin* mutant patient fibroblast cells also exhibited decreased levels of HSP60 and ATP5a proteins after IU1 treatment (Fig 3E), further supporting the notion that USP14**‐**induced mitochondrial clearance is PINK1/Parkin independent.

### USP14 inhibition and knockdown lead to mitochondrial membrane rupture

**Figure 4:**
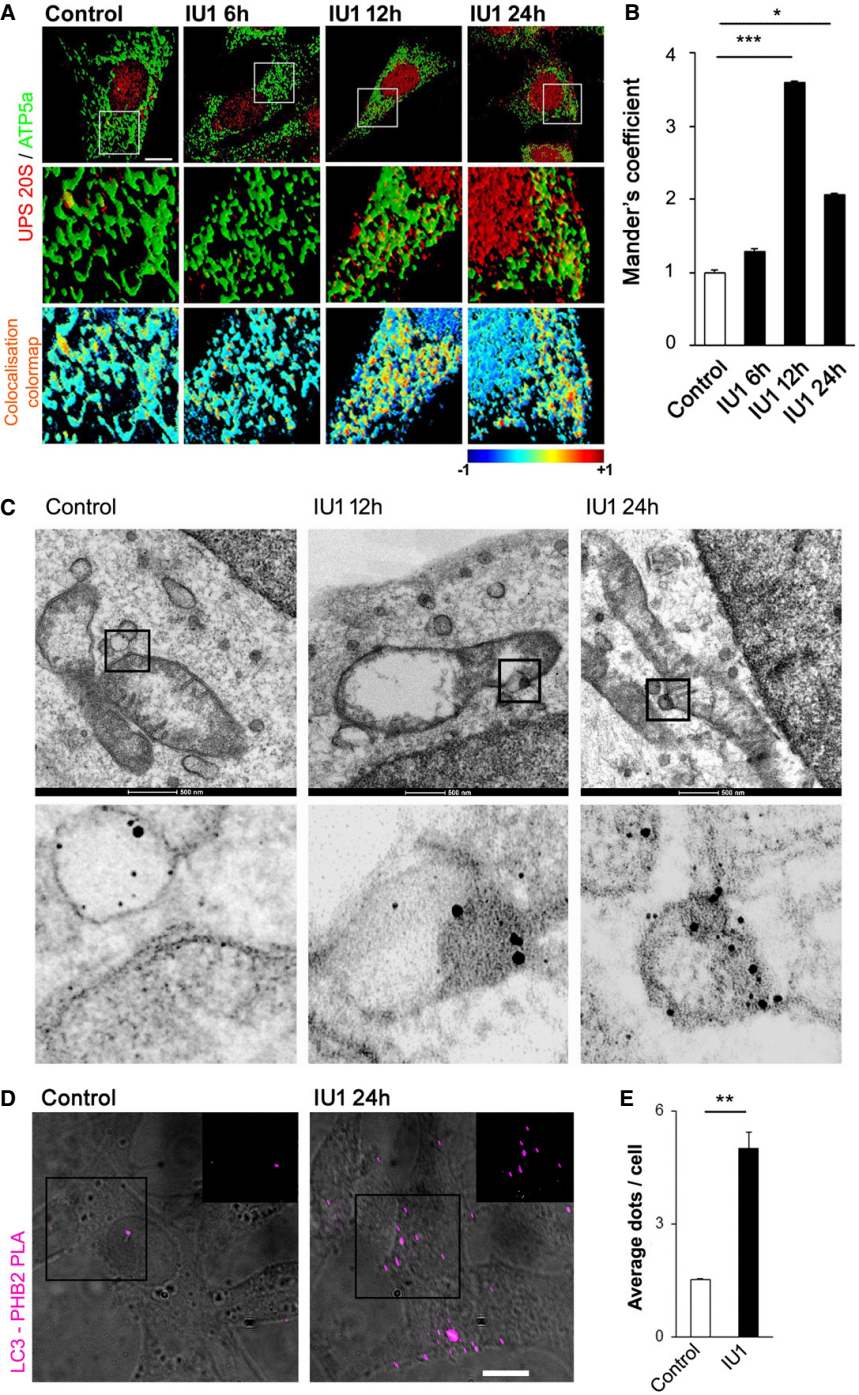
IU1 treatment mediates the translocation of the 20S proteasome subunit to mitochondria A, BSH‐SY5Y cells were immunostained for ATP5a (green) and 20S proteasome core (red) after IU1 treatment (6–24 h). Scale bar: 10 μm. Colour mapping of the co‐localisation of HSP60 and 20S proteasome from the selected regions was generated by ImageJ and is represented in the bottom panel. The intensity of the co‐localisation is represented in the colour map scale at the bottom of the panel. (B) 20S proteasome subunit and mitochondrial co‐localisation was quantified by measuring Mander's coefficient. Bar graphs represent the mean fold change (mean ± SEM) from three different experiments. At least 30 cells were evaluated for the calculations. ANOVA followed by Dunnett's test.CElectron microscopy images showing subcellular localisation of immunogold‐labelled 20S subunit of proteasome complex after 12 or 24 h of IU1 treatment. Experiments were repeated twice in two biological replicates with similar results. The contrast in the magnified region is enhanced from the original image to highlight the immunogold‐labelled signals.D, EProtein proximity ligation assay for Prohibitin 2 (PHB2) and LC3 after 24‐h IU1 treatment. Images showing signal are merged with bright‐field images to count the signal dots (cyan false colour) as depicted in the figure. In the inset, only fluorescence signal is shown, without merging with the bright field from the selected regions. Scale bar: 10 μm. (E) Bar graph represents the mean ± SEM of the number of signal dots per cell from three different experiments. Student's *t*‐test.Data information: *P ≤ 0.05, **P ≤ 0.01, ***P ≤ 0.001.

Given that translocation of the proteasome to mitochondria is required for mitophagy (Tanaka *et al*, 2010; Chan *et al*, 2011; Yoshii *et al*, 2011), we next monitored localisation of proteasome complex (20S subunit α+β) after IU1 treatment in SH‐SY5Y cells. Co‐localisation studies by confocal microscopy revealed an increased association of the 20S proteasome complex subunit with mitochondria after 12‐h IU1 treatment, which started to decrease at 24 h (Fig 4A and B). Increased association of the 20S proteasome complex subunit with mitochondria was also observed following 24 h of USP14 siRNA treatment (Appendix Fig S7A and B). To further investigate the subcellular localisation of the proteasome, we performed electron microscopy following immunogold labelling of the 20S proteasome complex subunit at 12 and 24 h of IU1 treatment (Fig 4C) or 24 h of USP14 siRNA treatment (Appendix Fig S7C). These results confirmed the confocal microscopy co‐localisation studies. During mitophagy, mitochondrial inner membrane proteins, including Prohibitin 2 (PHB2), are exposed and act as receptors for LC3 to form mitophagic vesicles (Wei *et al*, 2017). To evaluate this possibility, we performed proximity ligation assay for PHB2 and LC3 after 24 h of IU1 treatment (100 μM) in SH‐SY5Y cells. IU1‐treated cells exhibited an increased number of dots per cell, representative of increased PHB2–LC3 interaction (Fig 4D and E). Of note, IU1‐induced mitochondrial volume loss (Appendix Fig S8A and B) and the decrease in HSP60 and ATP5a levels (Appendix Fig S8C–E) were inhibited in PHB2^F/F^ cells upon expression of Cre recombinase, indicating that IU1‐mediated mitochondrial clearance is PHB2 dependent.

**Figure 5:**
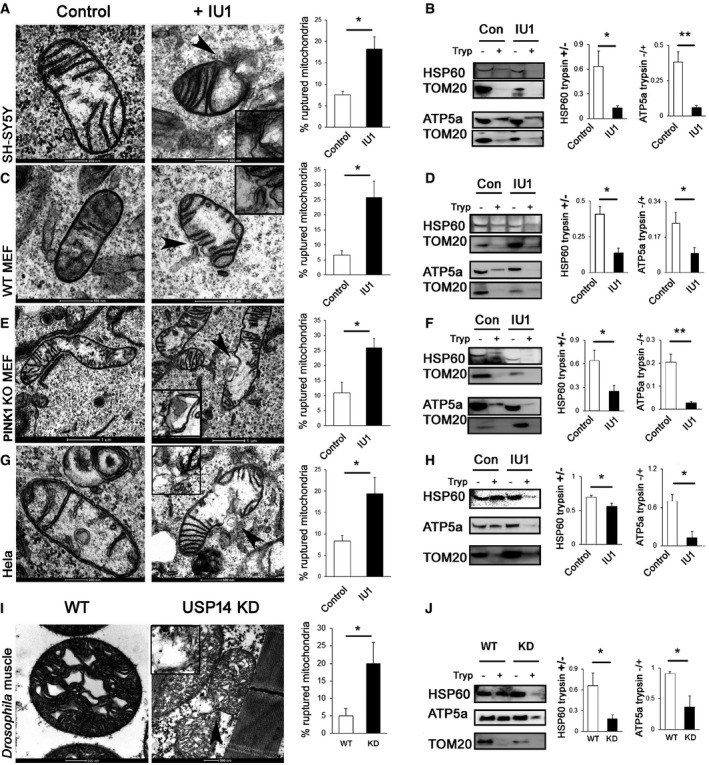
USP14 inhibition or knockdown ruptured mitochondrial membrane integrity A–J(A, C, E, G, I) Electron microscopy images of mitochondria from SH‐SY5Y, MEF WT, MEF PINK1 KO HeLa and *Drosophila* thoracic muscle, as indicated in the figure, with/without USP14 inhibition/knockdown. Arrowheads in the IU1‐treated groups indicate the mitochondrial rupture point. Bar graphs represent the mean (± SEM) of the percentage of ruptured mitochondria from at least three different experiments. For cells, a total of 150–300 mitochondria for the control group and 150–190 mitochondria from the IU1‐treated groups were counted. For flies, more than 200 mitochondria from each group were counted from five different experiments. (B, D, F, H, J) Representative immunoblots of isolated mitochondria from the indicated groups treated with/without trypsin and probed for HSP60, ATP5a and TOM20. Bar graphs represent the mean ± SEM of the ratio of densitometric levels of the indicated proteins (with:without trypsin) from at least three independent experiments. Student's *t*‐test; *P ≤ 0.05, **P ≤ 0.01. Source data are available online for this figure.

Given the increased association between mitochondrial inner membrane protein PHB2 and the autophagy machinery, we hypothesised that USP14 inhibition might result in altered mitochondrial membrane integrity. Indeed, close monitoring by electron microscopy showed that inhibition of USP14 leads to an increased number of mitochondria with ruptured mitochondrial membranes in SH‐SY5Y cells (Fig 5A). Of note, mitochondria isolated from IU1‐treated cells showed increased susceptibility to trypsin‐dependent digestion of HSP60 and ATP5a (Fig 5B), further supporting the hypothesis that USP14‐inhibited cells present altered mitochondrial membrane integrity. To exclude the possibility that the mitochondrial isolation procedure can interfere with mitochondrial membrane integrity, we treated digitonin‐permeabilised cells with trypsin and measured mitochondrial marker protein levels. The results were comparable to those obtained from trypsin‐digested isolated mitochondria (Appendix Fig S9). Inhibition of USP14 resulted in an increased number of mitochondria with ruptured mitochondrial membranes in WT MEF, PINK1 KO MEF and HeLa cells also (Fig 5C, E and G). Accordingly, digestion of isolated mitochondria from these cells resulted in increased susceptibility to trypsin‐dependent degradation of HSP60 and ATP5a (Fig 5D, F and H), corroborating the hypothesis that the effect of USP14 upon mitochondrial clearance is PINK1/Parkin independent. These results were confirmed in digitonin‐permeabilised cells as well (Appendix Fig S9).

We next monitored the effect of USP14 knockdown on mitochondrial membrane integrity *ex vivo*. Mitochondrial ultrastructure was evaluated by electron microscopy in thoracic muscle from control (actin‐Gal4) and USP14‐knocked‐down *Drosophila* (UAS USP14: actin‐Gal4). A marginal increase in the number of ruptured mitochondria was observed in the latter (Fig 5I). Mitochondria from 3‐ to 4‐day‐old control and USP14‐knockdown (KD) flies were isolated from the whole body and subjected to trypsin digestion assay. As previously observed *in vitro*, mitochondria from USP14‐knockdown (KD) flies resulted in increased susceptibility to trypsin‐dependent degradation of HSP60 and ATP5a (Fig 5J). In conclusion, USP14 inhibition and knockdown lead to mitochondrial membrane rupture *in vitro* and *ex vivo*.

### USP14 inhibition/knockdown rescues PINK1‐ and Parkin‐deficient flies by correcting mitochondrial dysfunction *in vivo*

To assess the therapeutic aspects of our findings and evaluate the effect of USP14 inhibition, we turned to two well‐established *Drosophila* models of impaired mitophagy, i.e. PINK1 and Parkin mutant flies. Before generating the USP14 KD PINK1 or Parkin mutant fly lines, we compared life span of control (actin‐Gal4) and USP14 KD flies. We found no significant difference in life expectancy of USP14 KD flies (Appendix Fig S10A). Respiration of isolated mitochondria from 3‐ to 6‐day‐old USP14 KD flies also showed no differences when compared with control flies (Appendix Fig S10B and C). Accordingly, mitochondria from thoracic muscles of USP14 KD flies showed no gross differences in distribution or morphology, except for the previously reported increased number of ruptured mitochondria in the USP14 KD group (Appendix Fig S10D).

**Figure 6:**
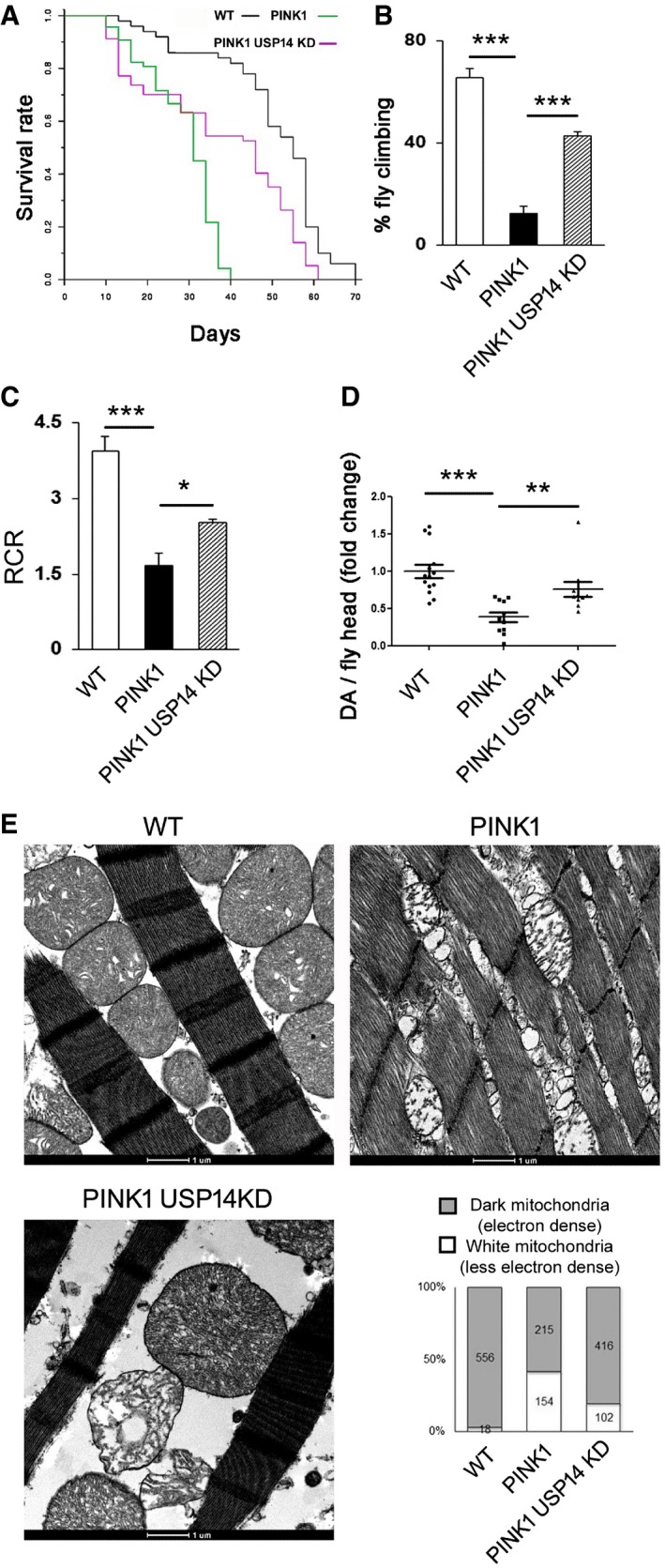
USP14 knockdown corrects life span, locomotor impairment, mitochondrial respiratory defects and muscle degeneration of PINK1‐deficient flies ALife span analysis of flies of the indicated genetic background. At least 50 flies were monitored from each group. Log‐rank test (Mantel–Cox test, Mantel–Haenszel test, *P* < 0.0001).BBar graph represents the mean ± SEM of the climbing performance of flies of the indicated genotypes. *n* = 4–7. ANOVA followed by Newman–Keuls test.CQuantitative analysis of respiratory fitness of isolated mitochondria extracted from flies of the indicated genotypes. Graph shows respiratory control rate (RCR) calculated as described in Materials and Methods and represents the mean ± SEM. *N* = 4–5, ANOVA followed by Newman–Keuls test.DDopamine content in the fly heads (15 days old) was measured by HPLC, and the individual fold change values are represented in the scatter plot. Graph represents the mean ± SEM. *N* = 14, 11 and 11 fly heads for control, PINK1 and PINK1+USP14 KD flies, respectively. ANOVA followed by Newman–Keuls test.EEnlarged representative TEM images of flight muscle mitochondria of the indicated genotypes. The experiment was repeated three times in replicate. Bar graph represents quantification of electron‐dense mitochondria (represented as grey bar) and depolarised mitochondria with ruptured cristae (represented as white bars) from the indicated genotypes.Data information: *P ≤ 0.05, **P ≤ 0.01, ***P ≤ 0.001.

Life span analysis revealed a reduced life expectancy in the PINK1 mutant (KO) flies (PINK1B9) as previously reported (Poddighe *et al*, 2013) (Fig 6A). Interestingly, USP14 down‐regulation ameliorated the shorter longevity of the PINK1 KO mutant flies (Fig 6A). USP14 knockdown also improved climbing ability (3 days old; Fig 6B), mitochondrial respiration (3–5 days old; Fig 6C) and dopamine levels in PINK1 mutant (KO) flies (15 days old; Fig 6D). Mitochondria in the thoracic muscle of PINK1 mutant flies were found to be less electron dense with complete disruption of the mitochondrial cristae when compared to the control group (Fig 6E), a phenotype that was partially corrected by USP14 knockdown (Fig 6E). In order to examine the effect of USP14 inhibition in flies, we first confirmed that IU1 treatment could induce fragmentation and decrease mitochondrial volume in S2R+ fly cells. We incubated S2R+ cells with IU1 (100 μM), which induced mitochondrial fragmentation and a decrease in mitochondrial volume after 24 h (Appendix Fig S11A–C). As USP14 inhibition in fly cells recapitulated the effects observed in mammalian cells, we mixed different concentrations of IU1 (1–100 μM) in the fly food to pharmacologically inhibit USP14 *in vivo*. Although none of the doses alone affected climbing ability (Appendix Fig S12A), increasing inhibitor concentrations decreased fly food uptake (Appendix Fig S12B). In the 1 μM IU1‐treated group, food consumption by the flies was normal (Appendix Fig S12B). Assessing chymotrypsin‐like activity of the proteasome complex indicated that this dose was sufficient to elevate proteasome activity *ex vivo* in flies (Appendix Fig S12C). Of note, 1 μM IU1 partially corrected the climbing ability of the PINK1 mutant (KO) flies (Appendix Fig S12D) and improved the number of electron‐dense mitochondrial population in thoracic flight muscles (Appendix Fig S12E). Again, IU1 treatment (1 μM) alone had no effect on life span (Appendix Fig S13A) or thoracic muscle mitochondrial morphology (Appendix Fig S13B).

**Figure 7:**
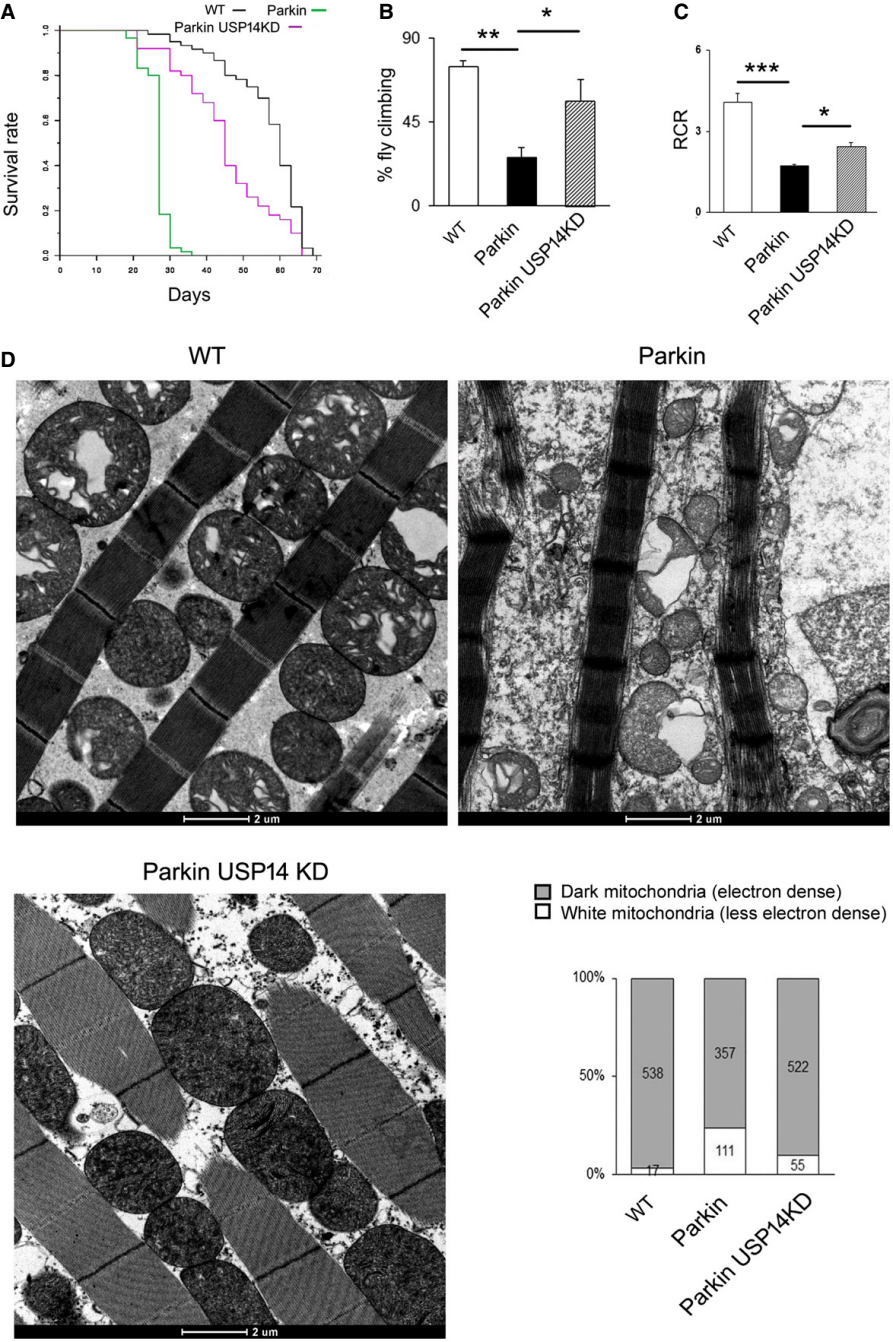
USP14 knockdown corrects life span, locomotor impairment, mitochondrial respiratory defects and muscle degeneration of Parkin‐deficient flies ALife span analysis of flies of the indicated genetic background. At least 50 flies were analysed for each group. Log‐rank test (Mantel–Cox test, Mantel–Haenszel test, *P* < 0.0001).BBar graph represents the mean ± SEM of the climbing performance of flies of the indicated genotypes. One‐way ANOVA followed by Newman–Keuls test, *n* = 3.CQuantitative analysis of respiratory fitness of isolated mitochondria extracted from flies of the indicated genotypes. Graph shows (mean ± SEM, *n* = 3 independent experiments) respiratory control rate (RCR) calculated as described. One‐way ANOVA followed by Newman–Keuls test.DEnlarged representative TEM images of flight muscle mitochondria of the indicated genotypes. The experiment was repeated three times in replicate. Bar graph represents quantification of electron‐dense mitochondria (represented as grey bar) and mitochondria with ruptured cristae (represented as white bars) from the indicated genotypes.Data information: *P ≤ 0.05, **P ≤ 0.01, ***P ≤ 0.001.

As previously reported for PINK1 mutant (KO) flies, Parkin mutant (KO) flies also exhibited a reduced life span in comparison with the control flies (Saini *et al*, 2011). USP14 knockdown improved the life expectancy of Parkin mutant flies (Fig 7A). Both genetic knockdown and pharmacological inhibition resulted in improved climbing activity (Fig 7B, Appendix Fig S14B) and partially corrected mitochondrial respiration (Fig 7C). Parkin mutant flies do not show significant dopamine depletion, which is previously reported by the other studies (Greene *et al*, 2003; Bingol *et al*, 2014). In Parkin mutant flies, mitochondria were not as severely affected as in PINK1 mutant flies, but the number of less electron‐dense mitochondria with ruptured cristae was higher than in the wild type (Fig 7D and Appendix Fig S14B). USP14 knockdown (Fig 7D) or IU1 treatment (Appendix Fig S14B) improved the thoracic flight muscle mitochondrial ultrastructure of the Parkin mutant flies as depicted in the bar graphs. We next tested induced pluripotent stem (iPS) cell lines derived from PD patients with PINK1 mutations (i.e. c.1366C>T; p.Q456X nonsense mutations; L2124) to assess the potential effect of USP14 inhibition. We tested the overall ATP content in iPS cells derived from PINK1 patients as previously reported (Morais *et al*, 2014). However, we did not find a significant decrease in the overall ATP content in cells derived from PINK1 patients as compared to age‐matched control fibroblasts (Appendix Fig S15). Since this result was not in accordance with what was previously reported, we could not test the potential protective effect of USP14 inhibition in this cell model.

## Discussion

Currently, the efficient clearance of dysfunctional mitochondria is modelled to be dependent on three key steps: (i) mitochondrial‐shaping and outer membrane protein post‐translational modifications (Gomes *et al*, 2011; Kageyama *et al*, 2014); (ii) UPS‐mediated mitochondrial membrane rupture (Tanaka *et al*, 2010; Yoshii *et al*, 2011); and (iii) engulfment of mitochondria by autophagic vesicles (Wei *et al*, 2017). Although the precise sequence of events is not clear, it is unequivocally accepted that both UPS and autophagic machinery are required for the completion of mitophagy. Therefore, enhancement of proteasome activity and increased autophagy together might lead to maximal mitophagy. Based on this rationale, recently deubiquitinating enzymes (DUBs) emerged as an alternative to antagonise Parkin/PINK1 dependency for mitophagy (Bingol *et al*, 2014; Durcan *et al*, 2014). In this regard, USP14 stands in a unique juncture because of its non‐selectiveness to the type of ubiquitination (Xu *et al*, 2015) that can influence both autophagy and proteasome activity, other than its well‐known direct inhibitory effect on UPS.

In this study, we found that USP14 modulation can affect mitophagy by inducing autophagy and by enhancing proteasome‐mediated mitochondrial rupture. Among the other factors, we found that USP14‐mediated mitophagy is dependent on mitochondrial dynamics proteins DRP1 and Mfn2 and occurs independently of PINK1 and Parkin. Most importantly, we report for the first time that USP14 knockdown or its pharmacological inhibition with specific inhibitor IU1 offers beneficial effects upon PINK1 or Parkin loss dependent phenotype *in vivo*.

USP14 can negatively regulate proteasome activity by increasing the docking time of the protein onto proteasome (Hanna *et al*, 2006; Lee *et al*, 2016; Kuo & Goldberg, 2017); however, the mechanism by which it does so remains unclear. USP14 is reported to affect the 20S gate opening as well as affect its activity allosterically (Peth *et al*, 2009; Kim & Goldberg, 2017). IU1, a specific inhibitor of USP14, binds to active or proteasome‐associated USP14 without showing any effect on other DUBs. This inhibitor was identified as an enhancer of proteasome activity from a molecular screening of small‐molecule library (Lee *et al*, 2010). In addition to its well‐documented activity as an enhancer of the proteasome, USP14 inhibition by IU1 has also been shown to lead to increased autophagy by stabilising K63 ubiquitination of Beclin1 (Xu *et al*, 2016). However, previous studies did not investigate the potential effect of IU1 or USP14 knockdown on mitophagy.

In accordance with earlier studies, we found increased degradation of proteasomal substrates and increased proteasome activity in IU1‐treated cells and upon USP14 knockdown. In addition, we found that genetic and pharmacological inhibition of USP14 increases autophagosome/autolysosome formation and enhance mitochondrial clearance in a human dopaminergic cell line. Mito‐Keima and LC3–mitochondria co‐localisation experiments along with electron microscopic studies also support that USP14 inhibition enhances mitophagy.

USP14 inhibition activates the proteasome and promotes autophagy, but how does that impact mitophagy? Proteasome can influence mitophagy by different ways. Proteasome‐mediated Miro degradation, which disrupts its complex with mitochondria, halts the movement of the organelle. This is essential for mitophagy (Wang *et al*, 2011; Hsieh *et al*, 2016). Also, effective mitophagy requires the translocation of the proteasome complex on mitochondria to rupture the mitochondrial membrane (Tanaka *et al*, 2010; Yoshii *et al*, 2011; Wei *et al*, 2017). In the current study, immunofluorescence and immunogold labelling assays confirmed the close physical interaction between the proteasome and mitochondria after USP14 inhibition or knockdown. Thus, one of the key downstream events in the activation of mitophagy (i.e. recruitment of proteasome complex onto mitochondria) is sustained upon USP14 inhibition. Furthermore, by employing two different approaches (i.e. electron microscopy analyses and trypsin digestion assays), we demonstrated a clear correlation between proteasome translocation to mitochondria and mitochondrial membrane rupture upon USP14 inhibition, both prerequisites for mitophagy.

Among the other factors, we found that USP14‐mediated mitophagy requires the mitochondria‐shaping proteins DRP1 and Mfn2. The dependency on DRP1 is due to the fact that mitochondria fragment before clearance (Gomes *et al*, 2011). On the other hand, Mfn2 overexpression has detrimental effects on mitochondrial quality control *in vivo* (Yun *et al*, 2014) and Mfn2 is indispensable for efficient mitophagy (Hailey *et al*, 2010; Hamasaki *et al*, 2013), operating as a molecular receptor for the recruitment of Parkin (Chen & Dorn, 2013). Mfn2 is also a key modulator of ER–mitochondria interaction at the mitochondria–endoplasmic reticulum (ER) membrane contacts (MERCs), which are sites of close juxtaposition between the two organelles that are required for DRP1‐mediated mitochondrial fission (de Brito & Scorrano, 2008; Friedman *et al*, 2011; Naon *et al*, 2016; which itself is a prerequisite for mitophagy), LC3 recruitment (Yang & Yang, 2013) and autophagosome formation (Hailey *et al*, 2010; Hamasaki *et al*, 2013). In the light of this, it is conceivable that USP14 inhibition depends on Mfn2 for mitophagy.

Next, we addressed whether USP14 inhibition‐mediated mitophagy is dependent on the PD proteins Parkin and PINK1, which are required for stress‐induced mitophagy (Narendra *et al*, 2008, 2010; Ziviani *et al*, 2010). Much has been unveiled about the functioning of the two proteins using *Drosophila* as the fast and reliable genetic model (Clark *et al*, 2006; Park *et al*, 2006; Yang *et al*, 2006; Poole *et al*, 2008). The canonical pathway illustrates that upon translocation to mitochondria, PINK1 becomes membrane potential‐dependently inactivated, by MPP‐AFG3L2‐PARL proteins (Narendra *et al*, 2008). In depolarised mitochondria, PINK1 recruits Parkin in a process that requires phosphorylation of Parkin (Murialdo *et al*, 1986; Merkwirth *et al*, 2008; Merkwirth & Langer, 2009), ubiquitin (Lee *et al*, 2016) and Mfn2 (Chen & Dorn, 2013). Parkin recruitment and activation results in the ubiquitination of a number of OMM‐resident proteins, including Mfn1 and Mfn2, TOM20, VDAC and Fis1, which is a prerequisite for the orchestration of the mitophagic process (Chen & Dorn, 2013). However, we did not evaluate the synergistic effect of IU1 upon stress‐induced PINK1/Parkin‐dependent mitophagy in the current study, and it remains open for further investigations. Here, we show that USP14 affects mitophagy independently of PINK1 or Parkin and that USP14 knockdown offers beneficial effects *in vivo* by correcting PINK1‐ or Parkin‐loss‐mediated decreased life span, locomotor deficit and mitochondrial dysfunction. In addition, and perhaps more importantly, our study showed that pharmacological inhibition of USP14 by IU1 is also protective *in vivo*, paving the way for a potential therapeutic potential for this drug. Our study showed no toxicity by IU1, in terms of life span and motor neuron survivability of *Drosophila*. Other studies also showed no toxicity of IU1 on neuronal population (Boselli *et al*, 2017; Min *et al*, 2017). However, while chronic intranasal administration is an option, the blood–brain barrier permeability of the drug is yet to be studied. The study by Lee *et al* (2010) has already shown that IU1 crosses cell membrane quite readily, advocating the possibility that it may also cross the blood–brain barrier; however, the metabolism of the drug needs further characterisation in higher mammals.

This study is the first effort to fully characterise the *in vivo* biology of a proteasome‐associated deubiquitinating enzyme in the context of mitophagy. A similar effort has also been made earlier by Bingol *et al* (2014) where a mitochondrial DUB showed antagonising effect with PINK1/Parkin. These data reveal a new mechanism for mitochondrial quality control mediated by USP14 supporting the notion of a physiological cross‐talk between mitophagy and the UPS *in vivo*. In conclusion, our study demonstrates for the first time how regulating UPS activity, autophagy and mitochondrial clearance may offer a therapeutic strategy to reduce the levels of aberrant proteins and organelles in cells and in the whole organism *in vivo*.

## Materials and Methods

### Antibodies and reagents

The following primary antibodies were used: anti‐LC3, anti‐ATP5a, anti‐OPA1, anti‐Mfn1, anti‐Mfn2 and anti‐proteasome 20S (α+β) were from Abcam; anti‐HSP60, anti‐TOM20 and anti‐PHB2 were from Santa Cruz Biotechnology; anti‐DRP1 and anti‐USP14 were from Cell Signaling; anti‐GFP was from Thermo Fisher Scientific; and anti‐Fis1 was from Enzo Life Sciences. HRP‐conjugated anti‐mouse or anti‐rabbit secondary antibodies were from Thermo Fisher Scientific, and Alexa Fluor 488/555‐conjugated anti‐mouse or anti‐rabbit secondary antibodies were from Life Technology. IU1 was purchased from Sigma‐Aldrich. Other sources of the reagents are indicated below when mentioned.

### Plasmids

Mito‐Keima was acquired from MBL International. Ub‐R‐GFP was obtained from Addgene. Mito‐YFP, DRP1 variant and Cre in adenoviral or retroviral backbone were procured from Dr. Luca Scorrano's laboratory (Cereghetti *et al*, 2008; Costa *et al*, 2010).

### Cell culture, transfection, treatment and viability assays

Human midbrain dopaminergic cell line SH‐SY5Y, mouse embryonic fibroblast (MEF) cells, HeLa cells and human control or Parkin mutant (exon 3 deletion plus Arg275 to Tryp) fibroblast cells were maintained in Dulbecco's modified Eagle's medium (DMEM; Thermo Fisher), supplemented with 10% heat‐inactivated foetal bovine serum (Thermo Fisher). PHB2^F/F^ cells were a kind gift from Dr. Thomas Langer. ATG7 KO cells were procured from Dr. Luca Scorrano's laboratory. Cells were maintained in a humidified incubator at 5% CO_2_ level. *Drosophila* S2R+ cells were cultured in Schneider's medium (Invitrogen) supplemented with 10% heat‐inactivated foetal calf serum (Sigma) and were maintained at 25°C.

Human fibroblasts from skin biopsy were obtained from B.A., a 55‐year‐old man who was diagnosed with PD at the age of 28 and presented an excellent response to a combination of levodopa and dopamine agonists until 2008. He then started complaining about motor fluctuations and involuntary movements and more recently developed pathological gambling, excessive impulsivity, aggressiveness and substance abuse (mainly cocaine). Cognitive testing in 2016 showed abnormalities in frontal executive and attention domains. His MRI revealed modest cortical and subcortical atrophy. Cells were grown in Dulbecco's modified Eagle's medium (DMEM) (Gibco) with the addition of 1% penicillin/streptomycin, 1% non‐essential amino acid solution, l‐glutamine and 10% FBS at 37°C with 5% CO_2_ atmosphere.

Cells were transfected with mito‐YFP/mito‐Keima/DRP1K38A‐YFP/Ub‐GFP plasmids using TransFectin™ (Bio‐Rad) and expressed for at least 24 h, and then split into different treatment groups to achieve a homogeneously transfected cell population among the vehicle/IU1 treatment groups. For USP14 (Cell Signaling) siRNA transfection, we used Oligofectamine™ reagent (Invitrogen) using 100 nM siRNA for each treatment. We used the same amount of scrambled siRNA as the control. USP14 knockdown was done for 24–72 h. For Cre transfection in PHB2^F/F^ cells, we used either adenoviral (for immunoblot) or retroviral transfection (for imaging) using standard protocol, exposing the cells at least for 24 h before starting the treatment.

For MTT assay, the cells were plated on 96‐well plates, and after the treatment period, 100 μl MTT solution (10 mg in 10 ml DMEM) was added. The cells were incubated for 2 h and then lysed using DMSO. The reading was taken at 550 nm.

For Hoechst and propidium iodide staining, we followed the standard protocol and concentrations (Hoechst: 1 μg/ml; and propidium iodide: 1.5 μM). Automated imaging and analysis was done using the Operetta imaging system (PerkinElmer).

### Chymotrypsin‐like activity assay of proteasome complex

After treatment, 25 μg whole‐fly tissue lysate in proteasome assay buffer (Tris 10 mM, pH 7.4, EDTA 1 mM, ATP 5 mM, DTT 5 mM and glycerol 20%, v/v) was taken onto proteasome activity buffer (Tris 50 mM, pH 7.4, EDTA 0.5 mM) and incubated with 50 μM substrate for chymotrypsin‐like activity (N‐Suc‐Leu‐Leu‐Val‐Tyr‐7‐amido‐4‐methylcoumarin) for 1 h at 37°C. The reading was taken at 380 nm excitation and 460 nm emission. MG132 was used to monitor the specificity, only protein lysate (no substrate) was kept in activity buffer to monitor autofluorescence, and only substrate (no protein sample) was used to monitor spontaneous breakdown of the substrate.

### Mitochondrial morphology analysis

For mitochondrial volume measurements, SH‐SY5Y/MEF cells were transfected with mito‐YFP plasmid and the next day plated on coverslips for the appropriate treatment. For S2R+ cells, mitochondria were stained with MitoTracker Green (Life Technologies). *Z*‐stack images (with 0.2 μm increments) of mitochondria from the cells were taken using a confocal microscope (Andromeda iMIC spinning disc live cell microscope, TILL Photonics, 60× objective). We used the ImageJ plugin Volumej for the 3D‐rendered representative images of mitochondria. The ImageJ plugin MitoLoc (Vowinckel *et al*, 2015) was used to determine the volume and fragmentation index. For fragmentation index calculation, we considered sum of fragment volume which individually constituted ≤ 20% of the total volume of mitochondria in a SH‐SY5Y cell, whereas for S2R+ cells, we considered 25%.

### Trypsin digestion assay in isolated mitochondria or permeabilised cells

Mitochondria were extracted from cells/flies by differential centrifugation. Each sample was homogenised using a Dounce glass–glass potter in mannitol–sucrose buffer (225 mM mannitol, 75 mM sucrose, 5 mM HEPES, 0.1 mM EGTA, pH 7.4) supplemented with 2% BSA. The samples were first centrifuged at 1,500 × *g* at 4°C for 6 min, and the supernatant was then centrifuged again for 6 min at 6,000 × *g*. The pellet was washed with mannitol–sucrose buffer and centrifuged again at 6,000 × *g* for 6 min. The pellet was then re‐suspended in a small volume of mannitol–sucrose buffer. Around 100 μg of mitochondria was treated with 200 μg/ml trypsin (TPCK treated; Worthington Biochemical) for 15–20 min (depending on the cell type) at 4°C in trypsin digestion buffer (10 mM sucrose, 0.1 mM EGTA/Tris and 10 mM Tris/HCl, pH 7.4); then, the samples were mixed with LDS sample buffer (Life Technologies) + β‐mercaptoethanol (Sigma). The samples were run for immunoblotting as described.

For the intact cells, first, the cells were digitonin‐treated (0.015%, 2 min), washed twice in PBS, re‐suspended in trypsin digestion buffer and divided into two parts. One part was treated with trypsin (200 μg/ml) for 15–20 min (depending on the cell type) at 4°C. Digestion was stopped by adding LDS sample buffer (Life Technologies) + β‐mercaptoethanol (Sigma). Quantification of the corresponding protein intensity is represented in the bar graphs as ratio of ‐ after trypsin : without trypsin digestion of the corresponding proteins. Protein levels of the OMM protein TOM20 were monitored as positive control for trypsin digestion efficiency.

### Mitochondrial respiration

The rate of mitochondrial O_2_ consumption was monitored using an Oxytherm System (Hansatech) with magnetic stirring and temperature control, maintained at 25°C. Isolated *Drosophila* mitochondria were re‐suspended in respiration buffer (120 mM KCl, 5 mM Pi‐Tris, 3 mM HEPES, 1 mM EGTA, 1 mM MgCl_2_, pH 7.2) in the Oxytherm System. O_2_ consumption was analysed according to the slope of the graph, after adding malate–pyruvate (10 mM) and ADP (200 μM). Respiratory control ratios state III versus state IV (200 mM ADP‐stimulated respiration over 1 μg/ml oligomycin‐administered respiration) were also determined from the registered graphs.

### Immunoblotting

In brief, the cells were collected in RIPA buffer (SH‐SY5Y or MEF cells) supplemented with 10 mM NEM, 10 mM MG132 (Sigma) and protease inhibitor cocktail (Roche). The lysate was incubated on ice for 30 min before being centrifuged at 10,000 × *g* for 15 min at 4°C. 25–40 μg protein was run on SDS gels and transferred to PVDF membranes. The following commercial antibodies were used: anti‐LC3 (1:1,000; Abcam), anti‐PHB2 (1:1,000; Santa Cruz Biotechnology), anti‐HSP60 (1:1,000; Santa Cruz Biotechnology), anti‐ATP5a (1:3,000; Abcam), anti‐actin (1:10,000; Chemicon), anti‐GFP (1:1,000; Invitrogen), anti‐TOM20 (1:5,000; Santa Cruz Biotechnology), anti‐Mfn1 (1:1,000; Abcam), anti‐Mfn2 (1:1,000; Abcam), anti‐USP14 (1:2,000; Cell Signaling), anti‐OPA1 (1:2,000; Abcam), anti‐DRP1 (1:1,000; Cell Signaling) and anti‐Fis1 (1:500; Enzo Life Sciences). For detection, secondary antibodies conjugated to HRP (Chemicon) were used (1:3,000), and immunoreactivity was visualised with ECL chemiluminescence (Amersham).

### Immunostaining and proximity ligation assay

For immunofluorescence analysis, the cells were plated on coverslips and grown for 24 h. After the treatment for the scheduled time period, the cells were washed with 0.1 M PBS (pH 7.2), fixed with 4% paraformaldehyde for 30 min, permeabilised with 0.1% Triton X‐100 (Sigma) for 5 min, blocked with 4% BSA (Sigma) for 30 min and finally incubated overnight with the primary antibody prepared in PBS containing 2% BSA and 0.05% Triton X‐100. The next day, after washing with PBS, the cells were incubated with a fluorescently tagged secondary antibody for 1 h. After washing with PBS, the cells were taken onto slides and mounted with anti‐fade mounting media (Invitrogen). *Z*‐stack images (with 0.2 or 0.4 μM increment) were taken using a confocal microscope (Andromeda iMIC spinning disc live cell microscope, TILL Photonics, 60× objective). The following primary antibodies were used for the study: LC3 (rabbit polyclonal, Abcam, 1:200), HSP60 (rabbit polyclonal, Santa Cruz Biotechnology, 1:200), ATP5a (mouse monoclonal, Abcam, 1:500) and proteasome 20S α+β (rabbit polyclonal, Abcam, 1:100). Secondary antibodies tagged with Alexa Fluor 488 or 555 (Invitrogen, 1:500) were used for the corresponding primary antibody.

For proximity ligation assay, we used a kit from Sigma‐Aldrich and followed the manufacturer's instructions. We fixed the subconfluent cells grown on coverslips with 4% PFA and incubated with Prohibitin 2 (Santa Cruz Biotechnology, 1:100) and LC3 antibody (Abcam, 1:200).

### Electron microscopy

Cells cultured in 24‐well plates or thoraces from adult male flies were fixed in 2% paraformaldehyde and 2.5% glutaraldehyde for 1 h/overnight. After rinsing in 0.1 M cacodylate buffer with 1% tannic acid, the samples were post‐fixed in 1:1 2% OsO_4_ and 0.2 M cacodylate buffer for 1 h. The samples were rinsed, dehydrated in ethanol and embedded in Epon. Ultrathin sections were examined using a transmission electron microscope. Data are represented as the number of dark and white mitochondria for electron‐dense or depolarised mitochondria, respectively.

For characterising autophagic vacuoles, we followed the standard guidelines (Klionsky *et al*, 2016).

For immunogold labelling, cells fixed with 4% PFA were permeabilised with saponin and incubated overnight with proteasome 20S antibody (Abcam, 1:100). Nanogold‐labelled secondary antibody (1:100) was incubated for 2 h, fixed with 1% glutaraldehyde and enhanced for 5–10 min by Gold enhancer (Nanoprobes).

### *Drosophila* stocks, treatment and climbing assay

*Drosophila* stocks were maintained under standard conditions at 25°C on agar, cornmeal and yeast food. Park25 mutants have been described before (Greene *et al*, 2003). PINK1B9 mutants were provided by Dr. J. Chung (KAIST, South Korea). Actin‐Gal4 strains were obtained from the Bloomington *Drosophila* Stock Center (Bloomington). UAS‐USP14 KD lines were obtained from VDRC Stock Center.

The USP14 inhibitor IU1 (Sigma) was administered to flies in the food. IU1 (or DMSO) was diluted in water to the desired concentration and used to reconstitute dry Formula 4–24 Instant *Drosophila* Medium (Carolina Biological Supply). Three‐day‐old mutant or control flies in groups of ten were fed on the supplemented food for 72 h, and subsequently, climbing assay was performed.

For the climbing assay, after scheduled time point, groups of 9–10 flies were collected and placed into empty transparent tubes (12 × 5 cm). Tubes were placed under a light source, and flies were gently tapped to the bottom of the tube. The number of flies that successfully climbed above the 6‐cm mark after 10 s was counted. Fifteen consecutive trials were performed for each experiment, and the average was taken.

Food intake was measured by adding food colouring (Patent Blue E131) in the fly food supplemented with vehicle or IU1. Flies kept for 3 days in the food were weighed (10/group) and homogenised in 10 volumes of PBS. The homogenate was centrifuged, and food colouring in the supernatant was measured by monitoring the absorption at 615 nm.

### Life span analysis

*Drosophila* from the mentioned genotypes were collected during 12 h after hatching and grouped into 20 flies per food vial. At least 50 flies were used for the analysis. The flies were transferred to fresh food (and fresh drug for the inhibitor treatment) every 3 days, and the number of dead flies was counted simultaneously.

### Dopamine measurement

Fifteen‐day‐old male *Drosophila* heads were dissected out and collected separately in 20 μl of ice‐cold 0.2 N perchloric acid. Then, the tissue was homogenised by sonication for 15 s and kept on ice for another 20 min and centrifuged at 12,000 × *g* for 10 min, and 5 μl of the supernatant was injected into a HPLC system equipped with a Rheodyne injector and a guard cell, set to +350 mV (E1 = +150 mV, E2 = −350 mV, s: 2 nA). A C_18_ ion‐pair, reverse‐phase analytical column (4.6 × 250 mm; 5 μm particle size; Agilent Technologies, USA) was used for the separation with a flow rate of 0.8 ml/min. The mobile phase contained 75 mM sodium phosphate monobasic monohydrate, 6% acetonitrile, 1.7 mM 1‐octane sulfonic acid and 25 μM EDTA (pH 3 ± 0.01). Dopamine values were determined by comparing with the standard peak value.

### Human fibroblasts and iPS cells derived from PINK1 PD‐causing mutation

The PINK1‐derived induced pluripotent stem (iPS) cells were obtained according to the previously described protocol (Twig *et al*, 2008), where previously published patient (L2124) and age‐matched control (HFF NT and L2131) lines were analysed.

### ATP determination

ATP levels measured in lysates from cell lines were determined as described in Harper *et al* (2018) using a luminescent solution (ATP Determination Kit; Invitrogen) according to the supplier's protocol. Luminescence values was measured on an EnVision Multilabel Reader (PerkinElmer), and luminescent ATP (nmol) was determined using a standard curve and normalised to total protein content (mg) measured by BCA assay (Pierce).

### Statistics

Representative graphs presented in the figures are mean ± standard error of the mean or as mentioned in the figure legends. Two‐tailed Student's *t*‐test or one‐way ANOVA was employed to determine the level of significance of comparisons between two groups or more than two groups, respectively, unless mentioned in the text. Dunnett's *post hoc* test was done to compare the experimental groups with the control group. The Newman–Keuls test was used for multiple comparisons where only three groups were involved, and Tukey's multiple comparison test was employed for comparisons where more than three groups were involved, as mentioned in the figure legends. *P* ≤ 0.05 was considered as significant difference. For survival study, we used log‐rank test (Mantel–Cox test, Mantel–Haenszel test). Please refer to Table EV1 for exact *n* and *P*‐values.

The sample size was chosen to ensure 80% power to detect an effect size of 0.75 on the basis of 5% type I error rate. No samples or animals were excluded from the analysis. Animals as well as samples were randomly chosen for treatment.

### Human subjects

The study protocol was approved by the ethics committee of Fondazione Ospedale San Camillo IRCCS, Venice, Italy. Informed consent was obtained from all subjects, and the experiments conformed to the WMA Declaration of Helsinki and the Department of Health and Human Services Belmont Report.

## Author contributions

JC designed and performed the experimental work and data analysis and contributed to manuscript writing. SvS designed, performed and analysed *in vivo* fly experiments. EM performed Hoechst/propidium iodide viability assay and some of the Western blots. FC performed electron microscopy acquisition and provided technical support. VF performed the HPLC experiment. AR generated PINK1‐derived iPS cells and performed ATP content analysis. AA provided human primary fibroblasts from *parkin* patient. CK and LB read the manuscript and intellectually contributed to the work. EZ designed the experiments and wrote the manuscript.

## Conflict of interest

The authors declare that they have no conflict of interest.

**The paper explained**

**Problem**

Cells need to clear damaged mitochondria through autophagy (mitophagy) for cell survival. Not surprisingly, perturbation of mitophagy has been correlated with age‐related neurodegenerative disorders. Mitophagy is a multi‐step process requiring both activation of autophagy and the proteasome. However, how to orchestrate both in order to stimulate mitophagy for therapeutic applications has not been fully addressed.

**Results**

Our results show that USP14 inhibition activates both the proteasome and autophagy and results in mitochondrial clearance. Mitochondrial fragmentation and mitochondrial membrane rupture to expose Prohibitin 2 were found to be essential to exhibit USP14 effect. *In vivo*, genetic or pharmacological inhibition of USP14 corrected mitochondrial dysfunction and locomotion performance of the PINK1 and Parkin mutant *Drosophila* model of Parkinson's disease (PD), an age‐related progressive neurodegenerative disorder whose aetiology has been directly correlated with deficient mitochondrial quality control.

**Impact**

Our study identified a novel therapeutic target antagonising mitochondrial dysfunction and *in vivo* PD‐related symptoms and may lead to a novel strategy design for neurodegenerative disorders.

## Supporting information



Appendix

Table EV1

Source Data for Appendix

Review Process File

Source Data for Figure 1

Source Data for Figure 2

Source Data for Figure 3

Source Data for Figure 5
