# Supplementary material for: USP14 inhibition corrects an in vivo model of impaired mitophagy
Source: EMBO Mol Med. 2018 Sep 24;10(11):e9014. doi: 10.15252/emmm.201809014 (PMC6220287; doi:10.15252/emmm.201809014)
Supplement: Supplementary file 1 — Appendix [file EMMM-10-e9014-s001.pdf]

USP14 inhibition corrects an *in vivo* model of impaired mitophagy

Joy Chakraborty[1], Sophia von Stockum[2], Elena Marchesan[2], Federico Caicci[1], Vanni Ferrari[1], Aleksandar Rakovic[3], Christine Klein[3], Angelo Antonini[4], Luigi Bubacco[1] and Elena Ziviani[1,2*]

Appendix

Table of content **Page**

Figure S1.. **2**

Figure S2.. **4**

Figure S3.. **6**

Figure S4.. **8**

Figure S5.. **9**

Figure S6..**10**

Figure S7..**11**

Figure S8..**13**

Figure S9..**14**

Figure S10..**16**

Figure S11..**18**

Figure S12..**19**

Figure S13..**22**

Figure S14..**23**

Figure S15..**24**

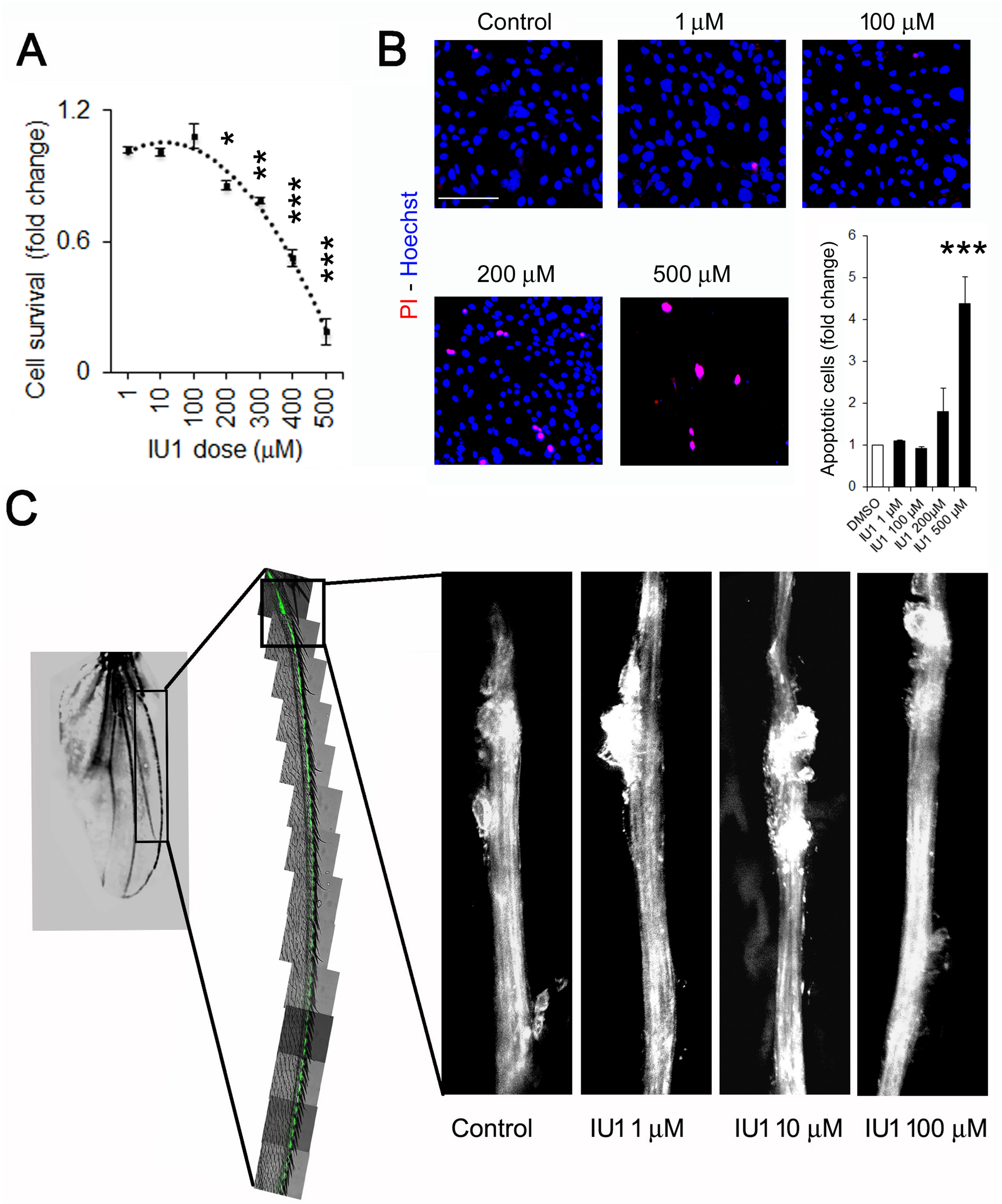

Figure S1. IU1 does not affect the survivability of SH-SY5Y cells and *Drosophila* wing neurons

A SH-SY5Y cells were treated with 1-500 µM IU1 for 24h and the cell survivability was monitored by MTT assay.

B SH-SY5Y cells were treated with different concentrations of IU1 for 24h and the cell viability was measured by propidium iodide / Hoechst staining. Scale bar 100 µm.

Line graph and bar graph represent fold change compared to the corresponding control group. Experiments are repeated 3 times and represented as mean ± SEM.

*$P \leq 0.05$, **$P \leq 0.01$, *** $P \leq 0.001$, ANOVA followed by Dunnet's test.

C 3-4 days old Drosophila expressing neuronal GFP was fed with IU1 (1-100 μM) mixed food for 3days. PFA fixed wings were dissected and the indicated area of the wing was imaged under confocal microscope at 60X for neuronal integrity. Images of neurons from one part of the wing is represented in the figure. n=4 control and n=5 IU1 treated flies were monitored.

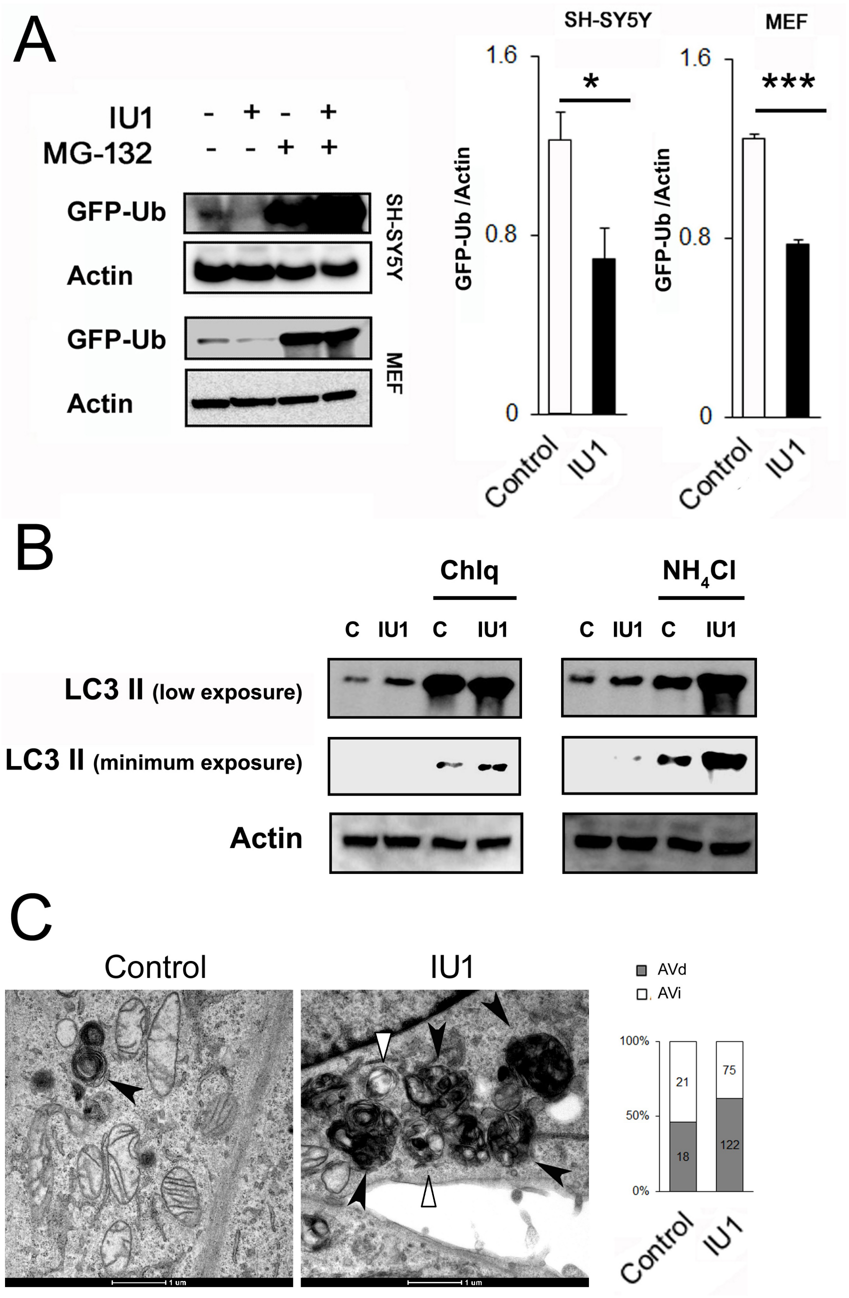

Figure S2. USP14 inhibition enhances proteasome activity and autophagy levels.

A SH-SY5Y / MEF cells expressing Ub-GFP were treated with IU1 (100 μM) for 24h and 48h respectively in combination with MG-132 as indicated and the level of GFP was monitored by western blot. Blots are representative of at least 3 different independent experiments. Bar graphs represent mean ± SEM. *P ≤ 0.05, ***P ≤ 0.001.

B SH-SY5Y cells were co-incubated with IU1 and chloroquine (chlq, 50 μM) or NH_4Cl (10 mM) for 24h and then probed for LC3-II by immunoblotting. Blots are representative of three different experiments.

C SH-SY5Y cells were treated with IU1 (100 μM) for 24h. The number of initial autophagic vacuoles (AVi) and degradative / late autophagic vacuoles (AVd) were counted from the electron microscope images. Black arrowheads and white arrowheads indicate AVd and AVi respectively. The experiment was done trice and images were taken from randomly selected fields. At least 30 cells were analysed for the quantification. Numeric figures in the graph represents the numbers counted.

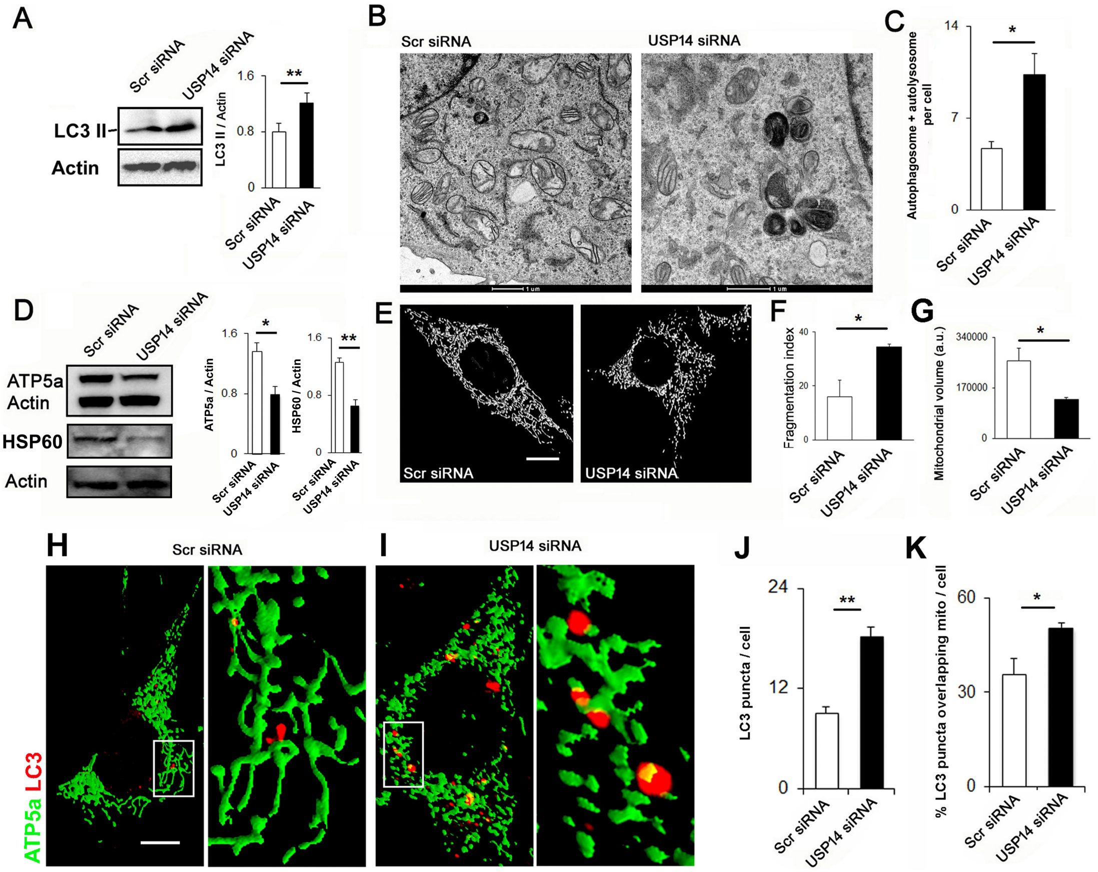

Figure S3. USP14 knockdown induces mitophagy.

A SH-SY5Y cells were treated with scramble / USP14 siRNA and the level of LC3 II protein was monitored by western blot. Bar graphs represent mean ± SEM of band intensities upon densitometric analysis of USP14 normalised by actin (n=3).

B-C Cells treated with scramble/ USP14 siRNA were fixed and examined under electron microscope. Bar graphs represent average number ± SEM of total number of autophagosomes and autolysosomes per cell. Total number of cells=60 for each group. The experiment was repeated 3 times.

D Western blot analysis of ATP5a and HSP60 protein levels measured from cell lysates treated with scramble / USP14 siRNA after 3 days. Bar graphs

represent mean ± SEM of band intensity upon densitometric analysis normalised by actin (n=3).

E-G Representative of the confocal images of mitochondria taken from SH-SY5Y cells expressing mito-YPF (E), treated with scramble / USP14 siRNA for 3 days and total mitochondrial volume / cell was measured. (F-G) Bar graphs represent fragmentation index and average mitochondrial volume / cell (± SEM) respectively, from 3 different experiments. At least 30 cells were considered for the experiment.

H-I SH-SY5Y cells were treated with scramble / USP14 siRNA (72h), fixed and immuno labelled for LC3 (H). Number of LC3 dots / cell (J) and LC3 dot overlapping mitochondria (K) were counted. At least 30 cells from 3 independent experiments were analysed. Graphs represent mean ± SEM. Students t test.

*P ≤ 0.05, **P ≤ 0.01.

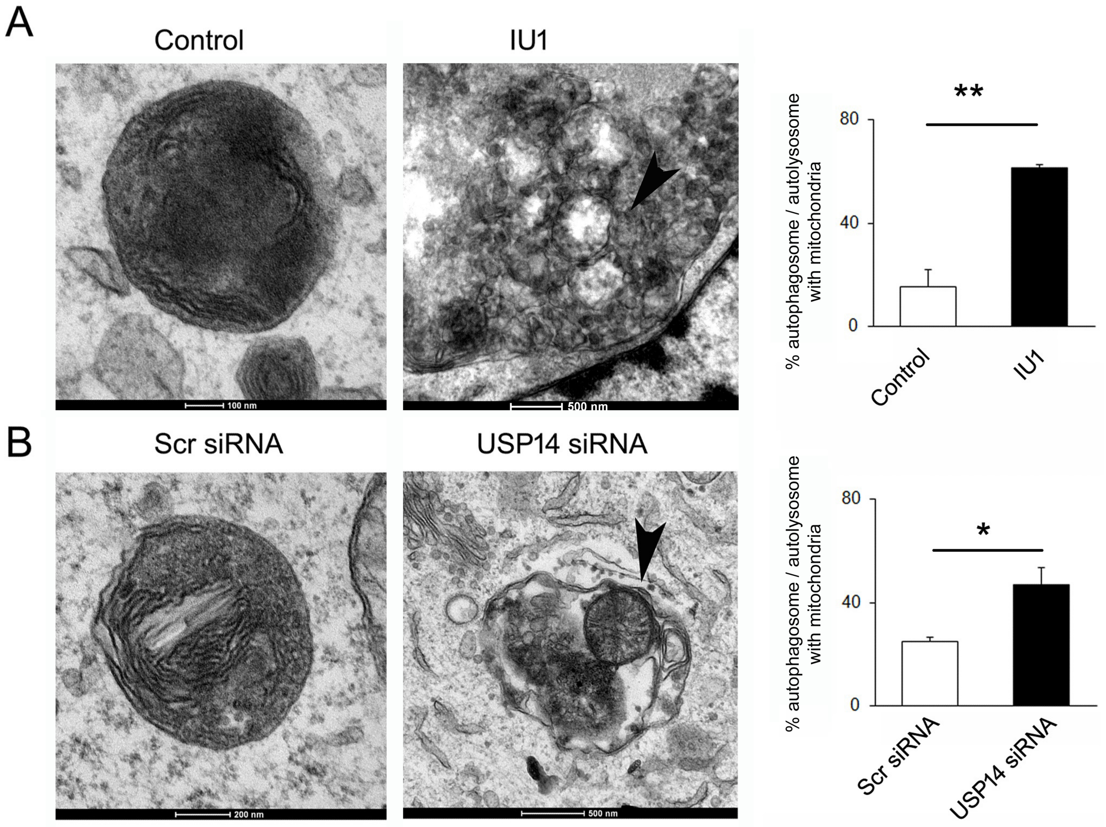

Figure S4. USP14 inhibition or knock down leads to mitophagy

A-B SH-SY5Y (A) cells were treated with IU1 (100 μM) or (B) USP14 siRNA for 3d were observed under electron microscope and autophagosomes / autolysosome with mitochondrial like structures inside were counted. 60-80 autophagosomes were counted from 30 cells (n=3). Bar graphs represent mean ± SEM of at least 3 independent experiments. Student's t test, *P ≤ 0.05, **P ≤ 0.01.

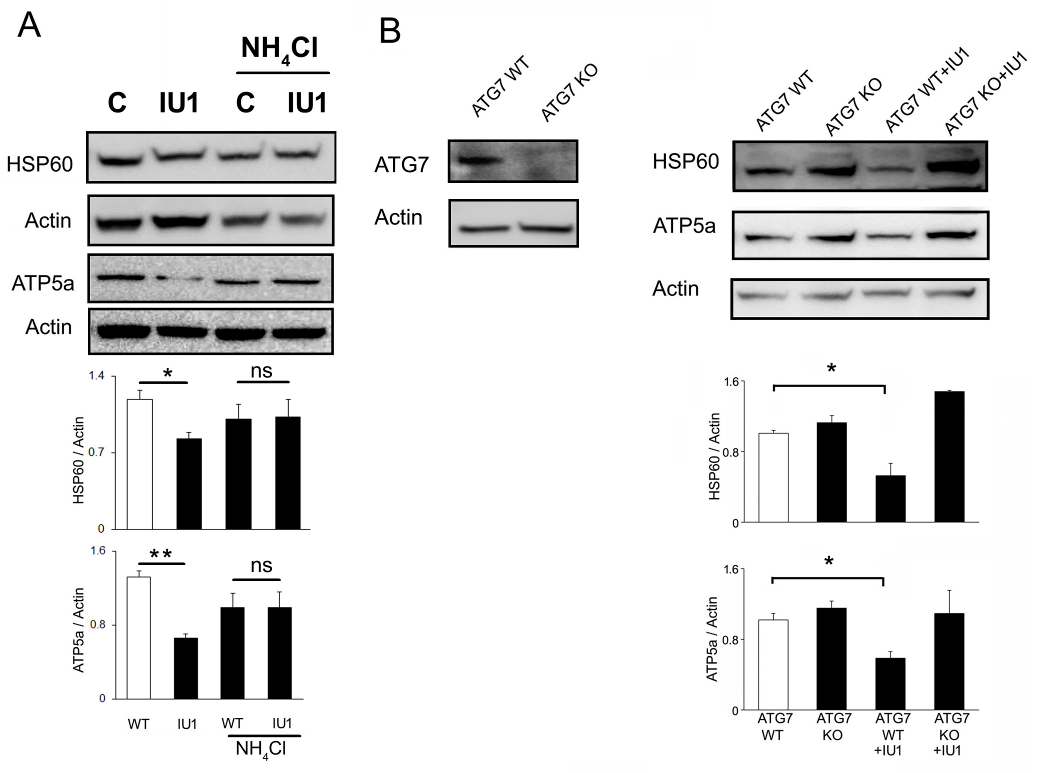

Figure S5. IU1 mediated mitophagy requires autophagic machinery.

A SH-SY5Y cells were co-incubated with IU1 (100 µM) and NH_4Cl (10 mM) for 24h and levels of LC3, HSP60 and ATP5a proteins were assessed by immunoblot. Bar graphs represent mean ± SEM of the ratio between densitometric levels of the indicated proteins (LC3, HSP60 and ATP5a, respectively) and those of Actin from of at least 3 independent experiments.

B ATG7 WT and KO MEF cells were incubated with 100 µM IU1 for 48h and levels of HSP60 and ATP5a were monitored by immunoblot. Bar graphs represent mean ± SEM of the ratio between densitometric levels of the indicated proteins (HSP60 and ATP5a, respectively) and those of Actin from of at least 3 independent experiments. Student's t test, $*P \leq 0.05$, $**P \leq 0.01$.

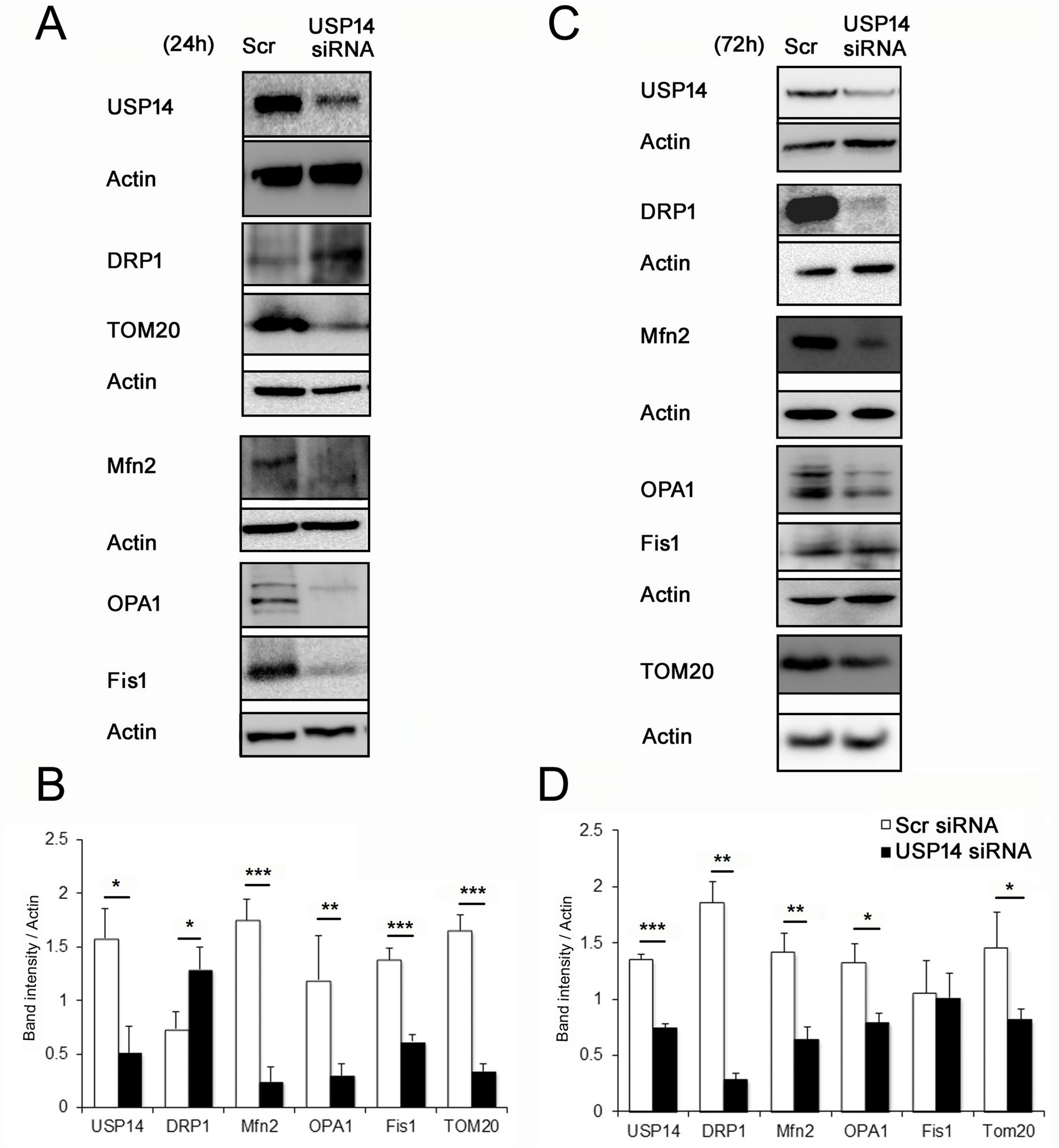

Figure S6. Effect of USP14 knockout on protein levels of mitochondrial shaping proteins.

A-D SH-SY5Y cells were administered with scramble or USP14 siRNA for (A) 24h or (C) 72h and levels of mitochondrial proteins (DRP1, Mfn2, Fis1, OPA1, TOM20) were analysed by western blot. Bar graphs represent mean ± SEM of the ratio between densitometric levels of the indicated proteins and those of Actin from of at least 3 independent experiments. Student's t test, $*P \leq 0.05$, $**P \leq 0.01$, $***P \leq 0.001$.

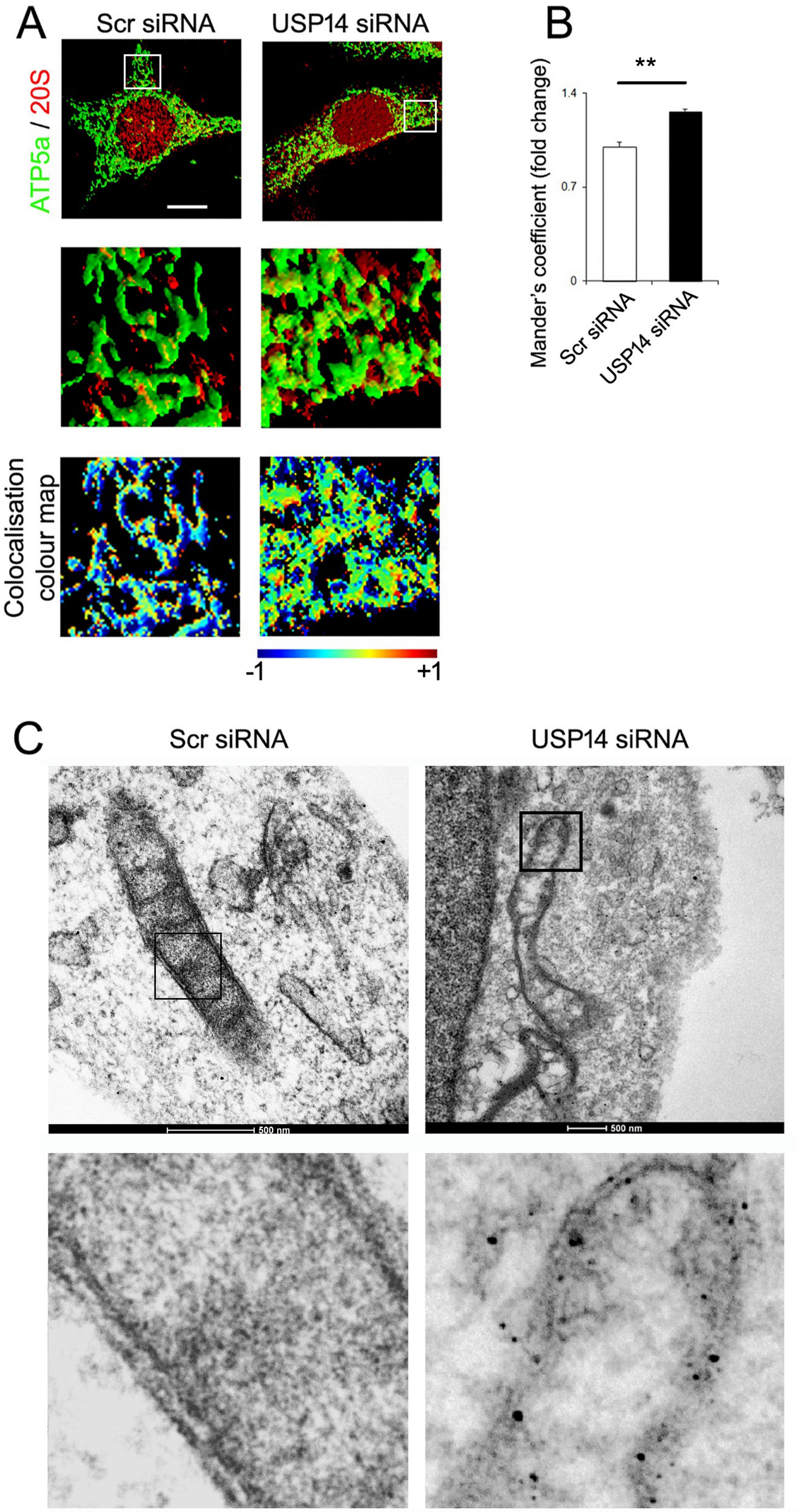
A
Scr siRNA
USP14 siRNA
ATP5a / 20S
Colocalisation colour map
-1
+1
B
**
Mander's coefficient (fold change)
1.4
0.7
0
Scr siRNA
USP14 siRNA
C
Scr siRNA
USP14 siRNA
500 nm
500 nm

Figure S7. USP14 knockdown mediates the translocation of the 20S proteasome subunit to mitochondria.

A-B SH-SY5Y cells were immunostained for ATP5a (green) and 20S proteasome subunit (red) after scramble/ USP14 siRNA treatment (A, 24h). Scale bar 10 μm. Colour mapping of the co-localisation of ATP5a and 20S proteasome subunit from the selected regions were generated by image j and represented in the bottom panel. Intensity of the co-localisation is represented in the colour map scale at the bottom of the panel. (B) The 20S proteasome subunit and mitochondria co-localisation was quantified by measuring Mander's coefficient. Bar graphs represent mean ± SEM from 3 different experiments. At least 30 cells were evaluated for the calculations. Student's t test, $**P \leq 0.01$.

C Electron microscopy images showing subcellular localization of immuno-gold labelled 20S subunit of proteasome complex after 24h of USP14 siRNA treatment. Experiments were repeated twice in two biological replicates with similar results. The contrast in the magnified region is enhanced from the original image to highlight the immunogold labelled signals

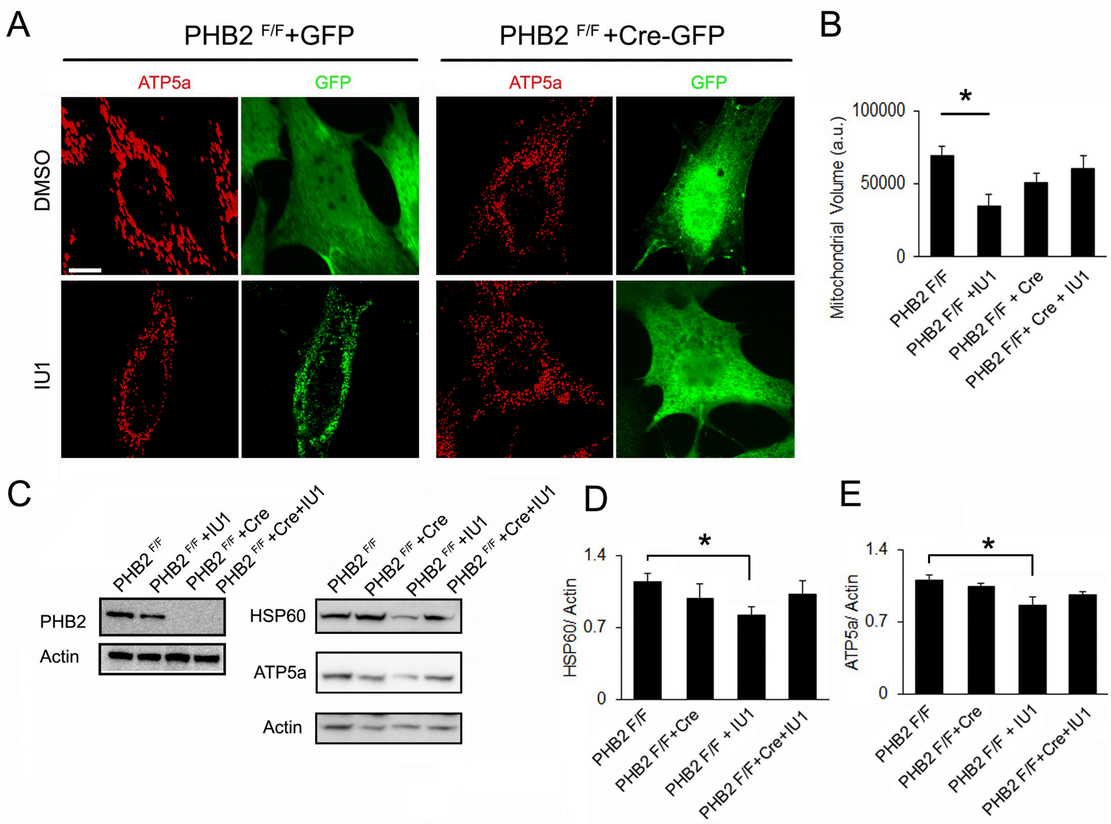

Figure S8. IU1 mediated mitophagy requires PHB2.

A-B PHB2 $^{F/F}$ MEF cells were transfected with either GFP or Cre-GFP and treated with IU1 (100 μM) for 48h. Cells were fixed and immunostained for ATP5a. Mitochondrial volume was measured by Image J. Bar graphs represent mean ± SEM of the average mitochondrial volume / cell from 3 different experiments. At least 30 cells were evaluated for the calculations.

C-E PHB2 $^{F/F}$ MEF cells were transfected with either GFP or Cre-GFP and treated with IU1 (100 μM) for 48h. Levels of PHB2, HSP60 and ATP5a were monitored by western blot (C). Bar graphs represent mean ± SEM of the ratio between densitometric levels of HSP60 (D) and ATP5a (E) and those of Actin from of at least 3 independent experiments.

Student's t test, $^{*}P \leq 0.05$

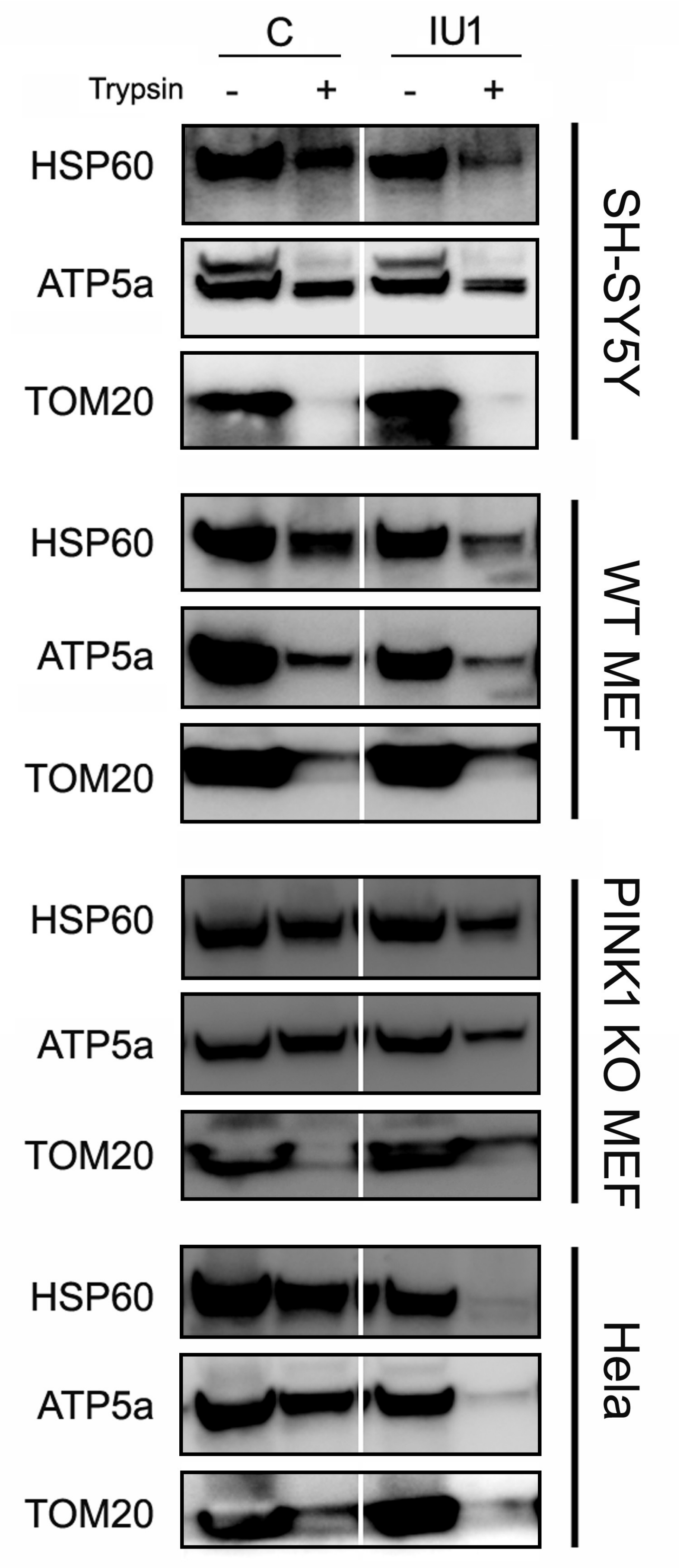
C
IU1
Trypsin
-
+
-
+
HSP60
ATP5a
TOM20
SH-SY5Y
HSP60
ATP5a
TOM20
WT MEF
HSP60
ATP5a
TOM20
PINK1 KO MEF
HSP60
ATP5a
TOM20
Hela

Figure S9. IU1 treatment affects mitochondrial membrane integrity.

Digitonin treated cells of the indicated sources, previously treated with or without IU1, were digested with trypsin before being lysated to evaluate protein levels of TOM20 (outer mitochondrial membrane resident protein), ATP5a (inner mitochondrial membrane resident protein) and HSP60 (mitochondrial matrix resident protein) by immunoblotting. Representative of two biological replicates.

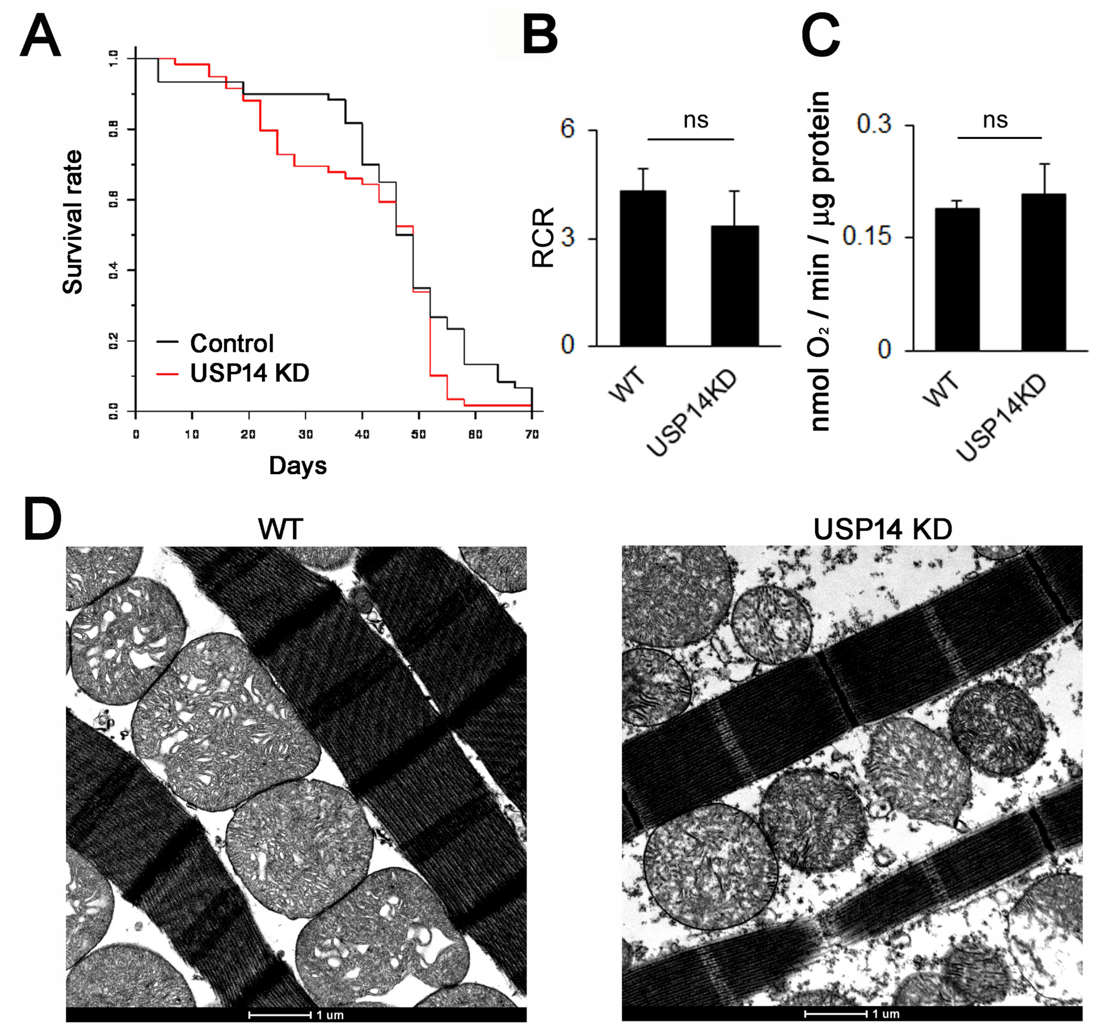

Figure S10. USP14 knock down *in vivo* shows no alteration in survivability, mitochondrial respiration and mitochondrial size / distribution.

A Lifespan analysis of male flies of the indicated genotypes. At least 60 flies were included in the experiment. Log-rank test (Mantel-Cox test, Mantel-Haenszel test, p=0.058).

B Quantitative analysis of respiratory fitness of isolated mitochondria extracted from 3-6 days old flies of the indicated genotype. Graph shows mean ± SEM (n=3 independent experiments) of RCR calculated as previously described.

C Bar graphs shows mean ± SEM (n=3 independent experiments) of oxygen (O_2) consumption during state III respiration (i.e. upon ADP stimulation).

D Enlarged TEM Images of indirect flight muscles from fly thoraces of the indicated genotypes.

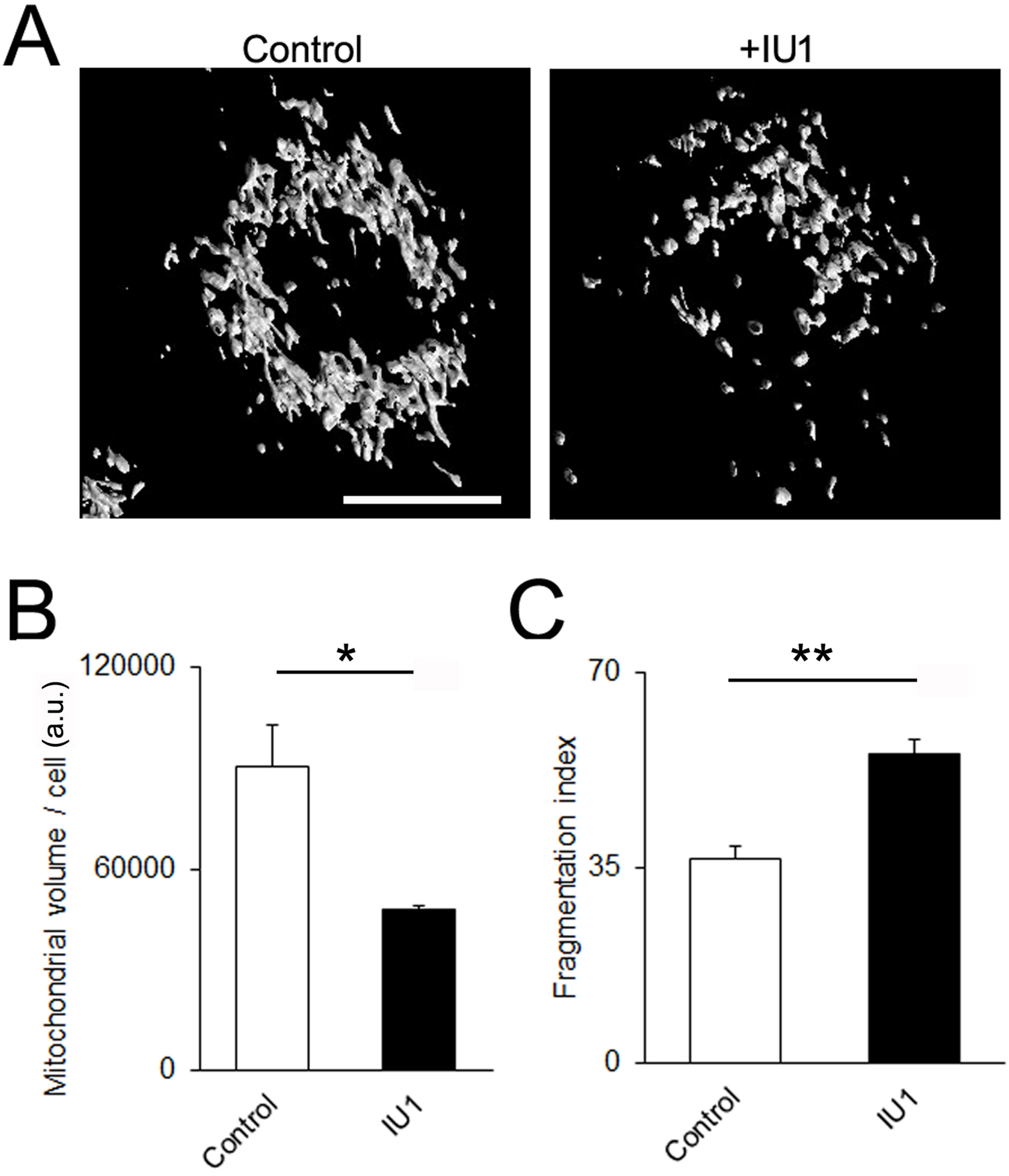

Figure S11. IU1 treatment induces mitochondrial volume loss in S2R+ fly cells.

A-C Confocal images (projection, Z stack) of S2R+ cells loaded with mito-tracker green and treated with DMSO (Control) or IU1 for 24h. Average mitochondrial volume / cell and fragmentation index were measured and represented as mean ± SEM (B and C respectively). At least 35 cells were analysed for the experiment. Student's t test; *P ≤0.05; **P ≤ 0.01.

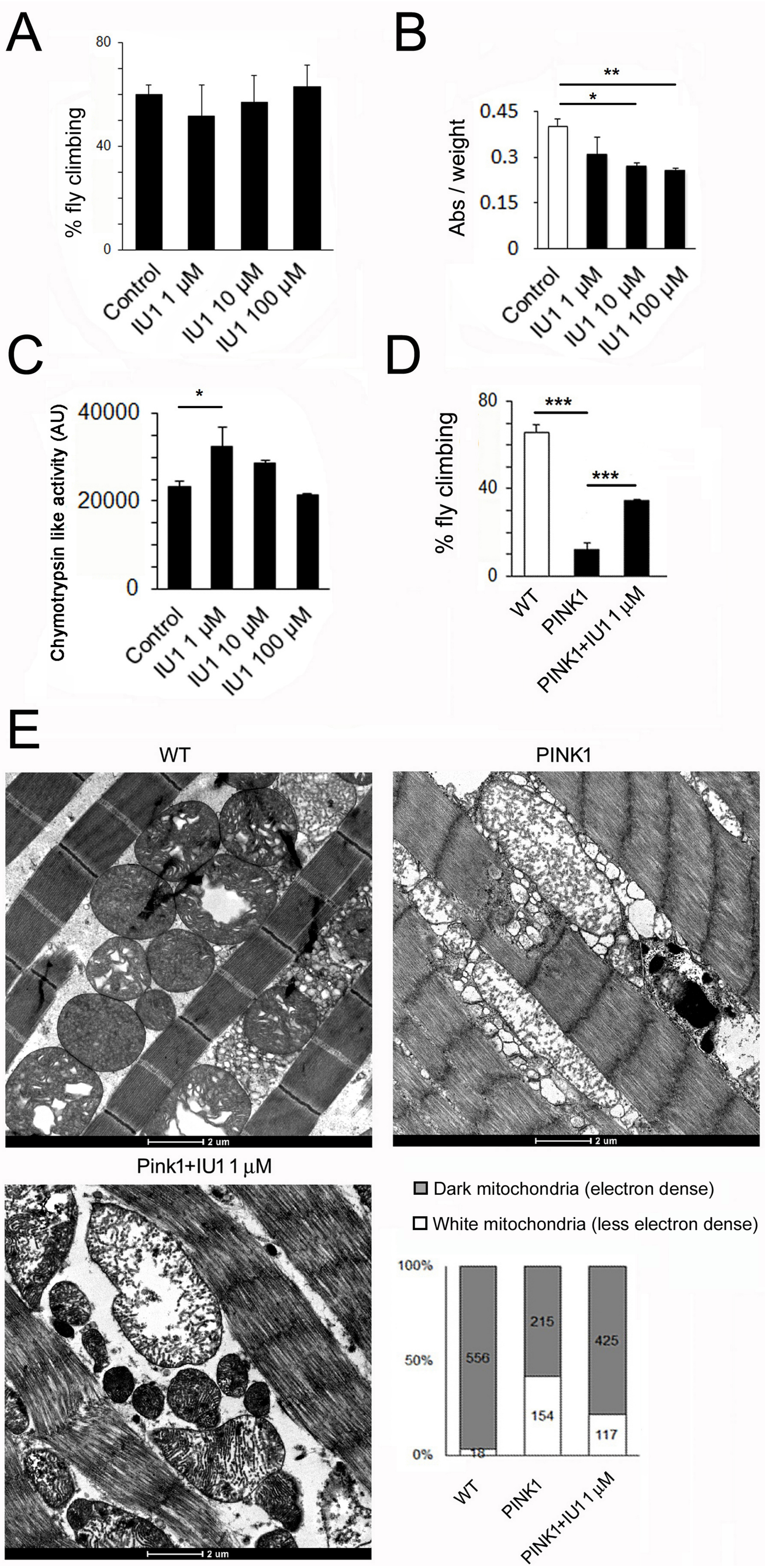
A
% fly climbing
80
40
0
Control
IU1 1 µM
IU1 10 µM
IU1 100 µM
B
Abs / weight
0.45
0.3
0.15
0
**
*
C
Chymotrypsin like activity (AU)
40000
20000
*
D
% fly climbing
***
WT
PINK1
PINK1+IU1 1 µM
E
WT
PINK1
Pink1+IU1 1 µM
2 um
Dark mitochondria (electron dense)
White mitochondria (less electron dense)
100%
50%
0%
556
18
215
154
425
117

Figure S12. IU1 consumed at the lowest dose attenuates PINK1 mutation induced climbing disability and mitochondrial cristae disarrangement.

A Graph bar shows mean ± SEM of the climbing performance of control flies fed with different concentrations of IU1 for three days from at least three independent experiments. ANOVA followed by Dunnet's test.

B Graph bar shows quantification of fly food uptake as measured by patent blue E131 absorbance in fly lysates derived from flies fed with increased IU1 concentration in the food. The absorbance of patent blue was measured at 615 nm and normalised by body weight. 3 different biological replicates with 10 flies each group. ANOVA followed by Dunnet's test.

C Chymotrypsin like proteasome activity of the tissue homogenates isolated from flies kept in food with different concentrations of IU1 (1-100 μM) for 3 days. Bar graphs represent mean ± SEM of 3 independent experiments. ANOVA followed by Dunnet's test.

D Climbing ability of 3-4 day old flies from the indicated genotypes and treated as mentioned was measured. Graph bars show mean ± SEM of climbing performance of at least three biological replicates. ANOVA followed by Tukey's test.

$^{*}P \leq 0.05$, $^{**}P \leq 0.01$, $^{***} P \leq 0.001$.

E Ultrastructural analysis of the indirect flight muscles from fly thoraces of the indicated genotypes. Images show transmission electron microscopy (TEM) images of thorax muscles from flies of the indicated genotypes. Total number of electron dense mitochondria (represented as dark bar, value represent the total number counted) and mitochondria with white

cristae (represented as white bar, value represent the total number counted) was calculated. The experiment was repeated 3 times.

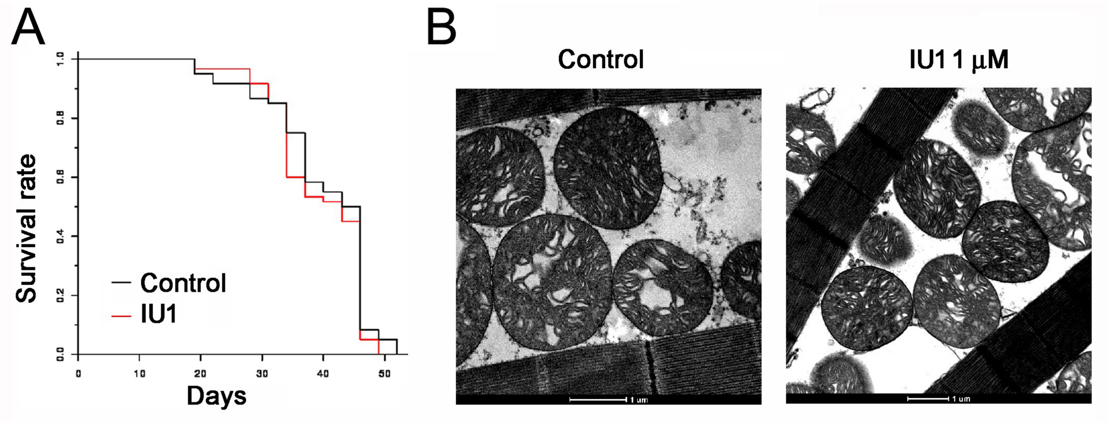

Figure S13. IU1 consumed at the lowest dose does not alter life span or mitochondrial morphology in male *Drosophila*

A Flies were kept with IU1 mixed food (1 µM) and life expectancy was analysed. At least 60 flies were considered for the experiment. Log-rank test (Mantel-Cox test, Mantel-Haenszel test, p=0.33).

B Flies were fed with vehicle or 1 µM IU1 for 3 days and mitochondria from the thoracic muscle was examined under electron microscope. The experiment was repeated 3 times.

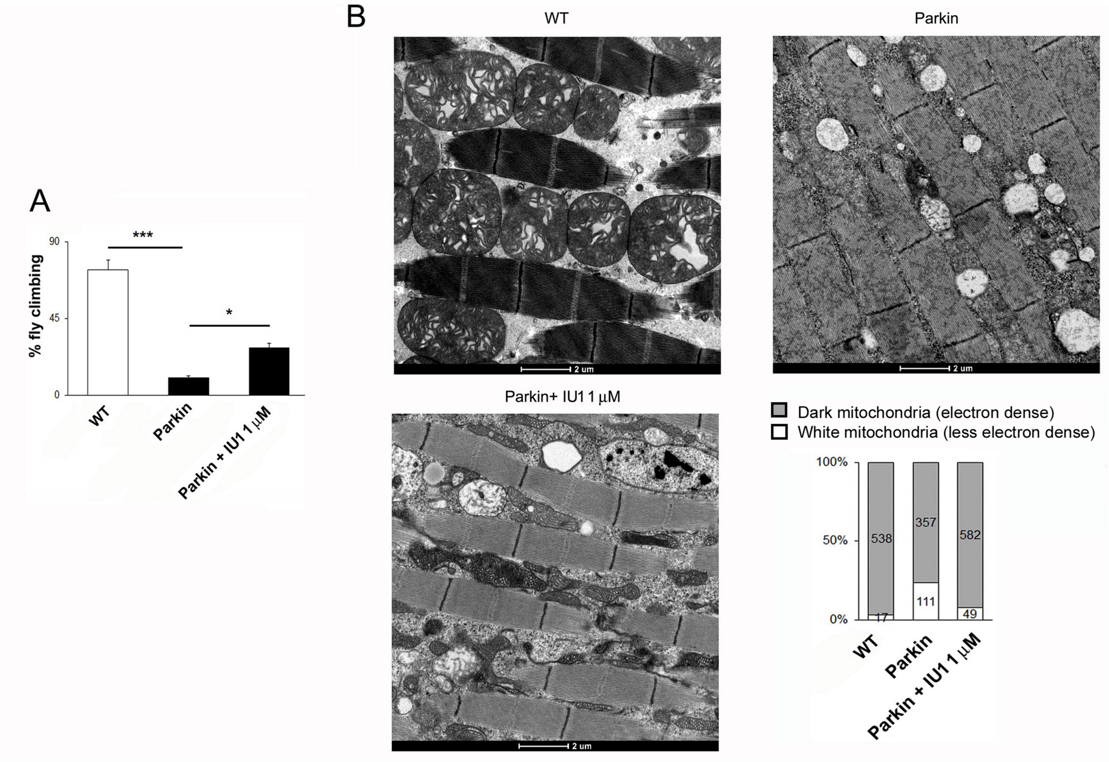

Figure S14. IU1 treatment improves Parkin mutation induced climbing disability and mitochondrial cristae disarrangement.

A 3 days old flies from the respective groups were analysed for their climbing ability. The experiment was repeated 3 times (Graphs represent mean± SEM). $*P \leq 0.05$; $***P \leq 0.001$. ANOVA followed by Newman-Keuls test.

B Fly thoracic muscle mitochondria from control (WT), Parkin mutant and Parkin mutant+1 μM IU1 treated flies were imaged under electron microscope and total number of electron dense mitochondria (represented as dark bar, value represents the total number counted) and mitochondria with white cristae (represented as white bar, value represents the total number counted) was calculated. The experiment was repeated 3 times.

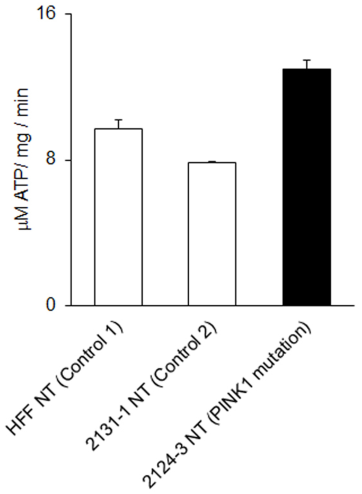

Figure S15. Human PD patient (a homozygous PINK1 c.1366C>T; p.Q456X)-derived iPS cells do not show defect in ATP levels.

Differentiated neuronal lines as mentioned were used to measure ATP levels and normalised by protein levels. Bar graphs represent mean± standard deviation; the experiment was done in duplicate.
