# Supplementary material for: USP14 inhibition corrects an in vivo model of impaired mitophagy
Source: EMBO Mol Med. 2018 Sep 24;10(11):e9014. doi: 10.15252/emmm.201809014 (PMC6220287; doi:10.15252/emmm.201809014)
Supplement: Supplementary file 2 — Table EV1 [file EMMM-10-e9014-s002.pdf]

Table EV 1 Exact n and p values and statistical test used

Figure	N	P value	Statistical analysis used
1A	8	0.0042	Two tailed t-test
1D	3	0.0102	Two tailed t-test
1E (ATP5a)	3	0.0212	Two tailed t-test
1E (HSP60)	3	0.0363	Two tailed t-test
1H	3	0.043	Two tailed t-test
1I	3	0.0226	Two tailed t-test
1K	3	0.0481	Two tailed t-test
1L	3	0.0062	Two tailed t-test
1N (Acidic pH)	Control= 6; IU1 =10	< 0.0001	Two tailed t-test
1N (Acidic / neutral pH)	Control= 6; IU1 =10	0.0154	Two tailed t-test
2B (TOM20)	3	0.0244	one way ANOVA/Dunnett's
2B (DRP1)	4	0.0101	one way ANOVA/Dunnett's
2B (Fis1)	3	0.0427	one way ANOVA/Dunnett's
2B (OPA1)	3	0.212	one way ANOVA/Dunnett's
2B (Mfn1)	4	P<0.0001	one way ANOVA/Dunnett's
2B (Mfn2)	4	0.0139	one way ANOVA/Dunnett's
2D	WT=4; WT+IU1=5	0.0427	Two tailed t-test
2D	DRP1KO=4; DRP1 KO+IU1=4	0.6734	Two tailed t-test
2D	Mfn1 KO=3; Mfn1 KO+IU1=4	0.0236	Two tailed t-test
2D	Mfn2 KO=4; Mfn2 KO+IU1=4	0.5473	Two tailed t-test
2F (HSP60)	WT=6; WT+IU1=6	0.0007	Two tailed t-test
2F (HSP60)	Mfn2 KO=3; Mfn2 KO+IU1=3	0.3484	Two tailed t-test
2F (HSP60)	WT+DRP1k38A=3; DRP1k38A+IU1=3	0.1229	Two tailed t-test
2F (HSP60)	DRP1 KO=3; DRP1 KO+IU1=3	0.1984	Two tailed t-test
2F (ATP5a)	WT=3; WT+IU1=3	0.0331	Two tailed t-test
2F (ATP5a)	Mfn2 KO=3; Mfn2 KO+IU1=3	0.8911	Two tailed t-test
2F (ATP5a)	WT+DRP1k38A=3; DRP1k38A+IU1=3	0.1764	Two tailed t-test
2F (ATP5a)	DRP1 KO=3; DRP1 KO+IU1=3	0.8137	Two tailed t-test
3D	WT MEF (+/- IU1)=3	0.0354	Two tailed t-test
3D	Hela (+/- IU1)=3	0.004	Two tailed t-test
3D	PINK1 KO (+/- IU1)=3	0.0182	Two tailed t-test
3E HSP60	Control MEF (+/-)=3	0.0315	Two tailed t-test
3E HSP60	Hela (+/- IU1)=4	0.0076	Two tailed t-test
3E HSP60	PINK1 KO (+/- IU1)=3	0.0478	Two tailed t-test
3E HSP60	Control human fibroblast (+/- IU1)=3	0.0013	Two tailed t-test
3E HSP60	PD patient fibroblast (+/- IU1)=3	0.016	Two tailed t-test
3E ATP5a	Control MEF (+/-)=5	0.0435	Two tailed t-test
3E ATP5a	Hela (+/- IU1)=4	0.0057	Two tailed t-test
3E ATP5a	PINK1 KO (+/- IU1)=3	0.0092	Two tailed t-test
3E ATP5a	Control human fibroblast (+/- IU1)=3	0.0142	Two tailed t-test
3E ATP5a	PD patient fibroblast (+/- IU1)=6	0.0003	Two tailed t-test
4B	3	0.0002	one way ANOVA/Dunnett's
4E	3	0.0012	Two tailed t-test
5A	3	0.0219	Two tailed t-test
5B HSP60	4	0.034	Two tailed t-test
5B ATP5a	4	0.0038	Two tailed t-test
5C	3	0.0257	Two tailed t-test

Figure	N	P value	Statistical analysis used
5D HSP60	3	0.0111	Two tailed t-test
5D ATP5a	4	0.046	Two tailed t-test
5E	3	0.0318	Two tailed t-test
5F HSP60	4	0.041	Two tailed t-test
5F ATP5a	3	0.0073	Two tailed t-test
5G	3	0.0483	Two tailed t-test
5H HSP60	4	0.036	Two tailed t-test
5H ATP5a	3	0.0261	Two tailed t-test
5I	5	0.0488	Two tailed t-test
5J HSP60	4	0.0436	Two tailed t-test
5J ATP5a	3	0.0418	Two tailed t-test
6A	WT=50; Pink1=120; Pink1 USP14 KD=57	< 0.0001	Log-rank test
6B	WT=7; PINK1=5; PINK1 USP14KD= 4	<0.0001	One way ANOVA/Newman-Keuls
6C	WT=5; PINK1=5; PINK1 USP14 KD=4	0.0001	One way ANOVA/Newman-Keuls
6D	WT=14; PINK1=11; PINK1 USP14 KD=11	<0.0001	One way ANOVA/Newman-Keuls
7A	WT=60; Parkin=60; Parkin USP14 KD=50	0.0001	Log-rank test
7B	WT=3; Parkin=3; Parkin USP14 KD=3	0.0095	One way ANOVA/Newman-Keuls
7C	WT=3; Parkin=3; Parkin USP14 KD=3	0.0005	One way ANOVA/Newman-Keuls

Figure	N	P value / P value summery	Statistical analysis used
S1A	3	P<0.0001	one way ANOVA/Dunnett's
S1A	3	ctrl vs 200uM p=0.0025	Two tailed t-test
S1A	3	ctrl vs 300uM p=0.0003	Two tailed t-test
S1A	3	ctrl vs 400uM p=0.0002	Two tailed t-test
S1A	3	ctrl vs 500uM p=0.0002	Two tailed t-test
S1B	3	0.0003	one way ANOVA/Dunnett's
S2A SH-SY5U	4	0.0302	Two tailed t-test
S2A MEF	3	0.0002	Two tailed t-test
S3A	6	0.0018	Two tailed t-test
S3C	3	0.0285	Two tailed t-test
S3D ATP5a	3	0.0285	Two tailed t-test
S3D HSP60	3	0.0038	Two tailed t-test
S3F	3	0.0413	Two tailed t-test
S3G	3	0.0423	Two tailed t-test
S3J	3	0.0031	Two tailed t-test
S3K	3	0.0427	Two tailed t-test
S4A	3	0.0022	Two tailed t-test
S4B	3	0.029	Two tailed t-test
S5A ATP5a (WT vs IU1 only)	4	0.0002	Two tailed t-test
S5A ATP5a (WT vs IU1 + NH4Cl)	4	0.9892	Two tailed t-test
S5A HSP60 (WT vs IU1 only)	4	0.0143	Two tailed t-test
S5A HSP60 (WT vs IU1 + NH4Cl)	4	0.9354	Two tailed t-test
S5B HSP60 (WT vs WT+IU1)	3	0.0414	Two tailed t-test
S5B HSP60 (KO vs KO+IU1)	3	0.2059	Two tailed t-test
S5B ATP5a (WT vs WT+IU1)	3	0.044	Two tailed t-test
S5B ATP5a (KO vs KO+IU1)	3	0.2229	Two tailed t-test
S6B USP14	3	0.0445	Two tailed t-test
S6B DRP1	3	0.0413	Two tailed t-test
S6B Mfn2	4	0.0007	Two tailed t-test
S6B OPA1	3	0.0033	Two tailed t-test
S6B Fis1	4	0.0006	Two tailed t-test
S6B TOM20	4	0.0002	Two tailed t-test
S6D USP14	3	0.0005	Two tailed t-test
S6D DRP1	3	0.0012	Two tailed t-test
S6D Mfn2	4	0.0092	Two tailed t-test
S6D OPA1	4	0.0275	Two tailed t-test
S6D Fis1	3	0.9079	Two tailed t-test
S6D TOM20	5	0.0397	Two tailed t-test
S7B	3	0.0028	Two tailed t-test
S8B (PHB2 F/F vs F/F + IU1)	4	0.0135	Two tailed t-test
S8B (PHB2 F/F Cre vs F/F Cre + IU1)	4	0.4843	Two tailed t-test
S8D (PHB2 F/F vs F/F + IU1)	4	0.0264	Two tailed t-test
S8D (PHB2 F/F Cre vs F/F Cre + IU1)	4	0.8414	Two tailed t-test
S8E (PHB2 F/F vs F/F + IU1)	4	0.0479	Two tailed t-test

Figure	N	P value	Statistical analysis used
S8E (PHB2 F/F Cre vs F/F Cre + IU1)	4	0.1528	Two tailed t-test
S10A	60	0.058	Log-rank test
S10B	3	0.465	Two tailed t-test
S10C	3	0.6967	Two tailed t-test
S11B	3	0.0243	Two tailed t-test
S11C	3	0.0051	Two tailed t-test
S12A	4	0.8274	one way ANOVA/Dunnett's
S12B	3	0.036	one way ANOVA/Dunnett's
S12B	3	ctrl vs IU1 1uM; p=0.1996	Two tailed t-test
S12B	3	ctrl vs IU1 10uM; p=0.0102	Two tailed t-test
S12B	3	ctrl vs IU1 100uM; p=0.0038	Two tailed t-test
S12C	3	0.0148	one way ANOVA/Dunnett's
S12D	5	0.0292	one way ANOVA/Tukey
S13A	60	0.33	Log-rank test
S14A	4	P<0.0001	One way ANOVA/Newman-Keuls
S15	2		
