# Supplementary figures and images for: USP14 inhibition corrects an in vivo model of impaired mitophagy
Source: EMBO Mol Med. 2018 Sep 24;10(11):e9014. doi: 10.15252/emmm.201809014 (PMC6220287; doi:10.15252/emmm.201809014)

Fig S2A

SH-SY5Y

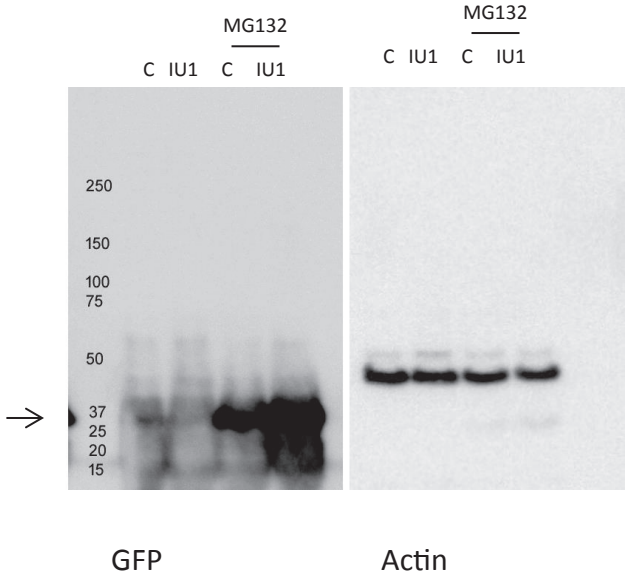

MEF

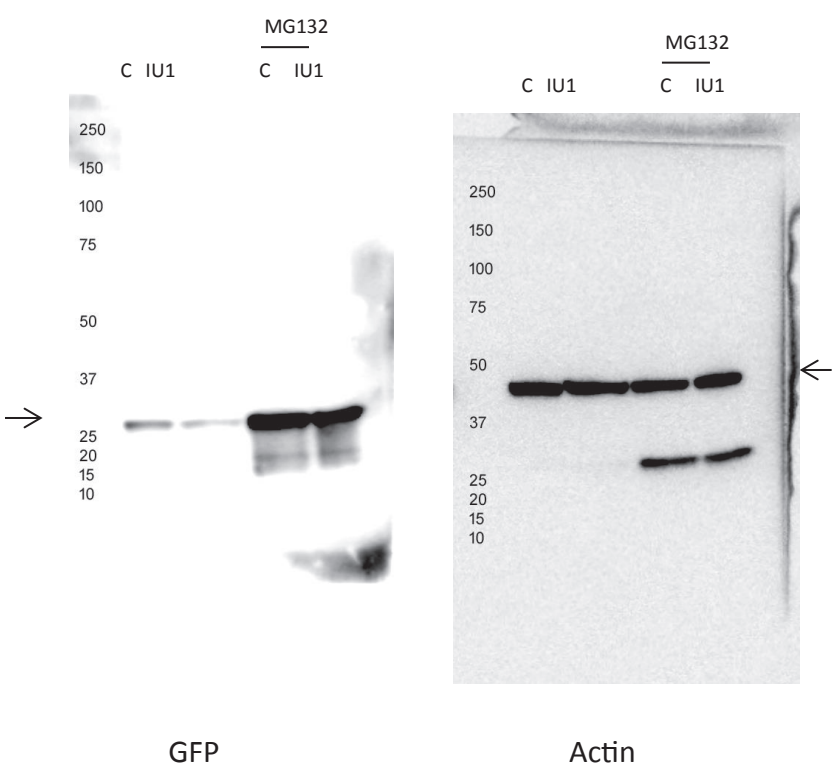

Fig S2B

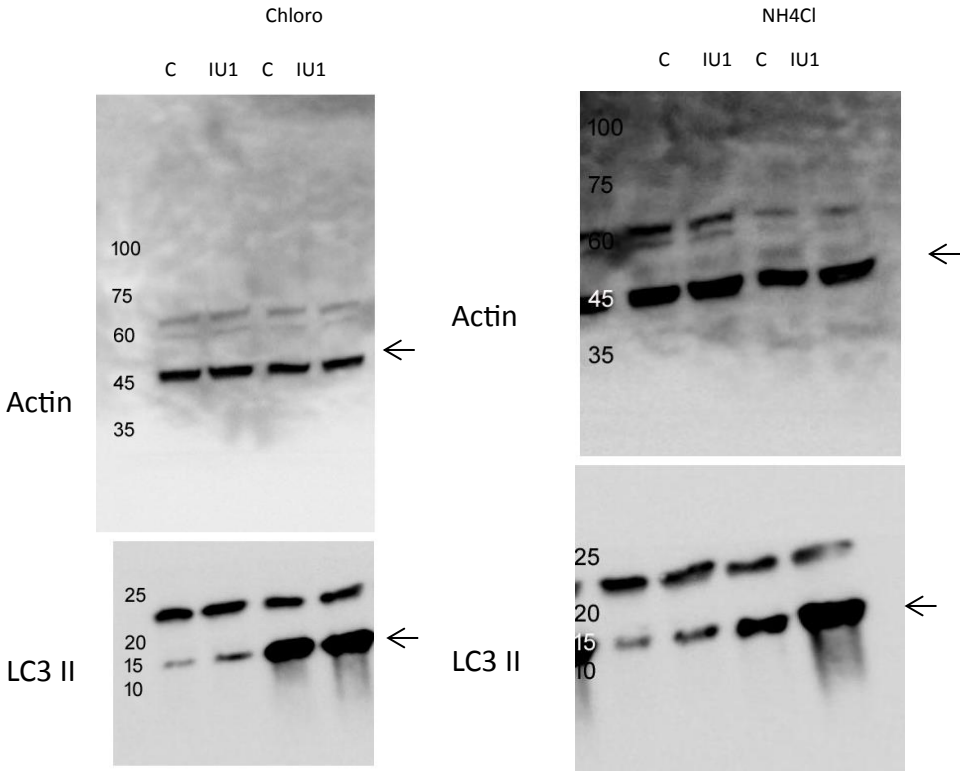

Fig S3A

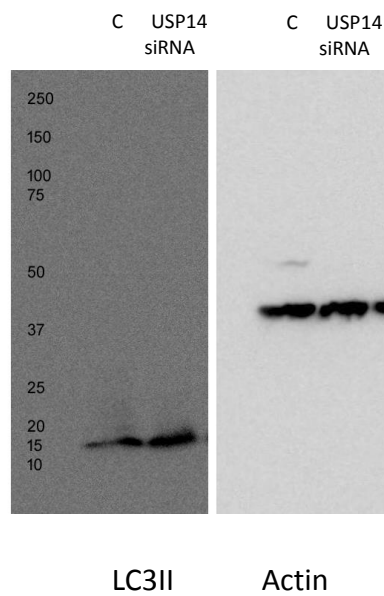

Fig S3D

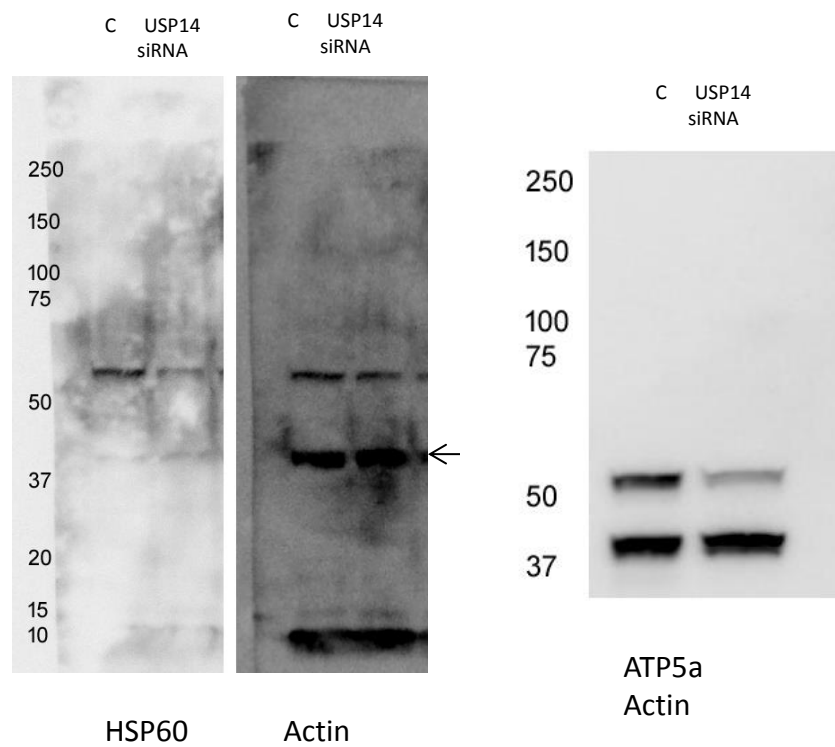

Fig S5A

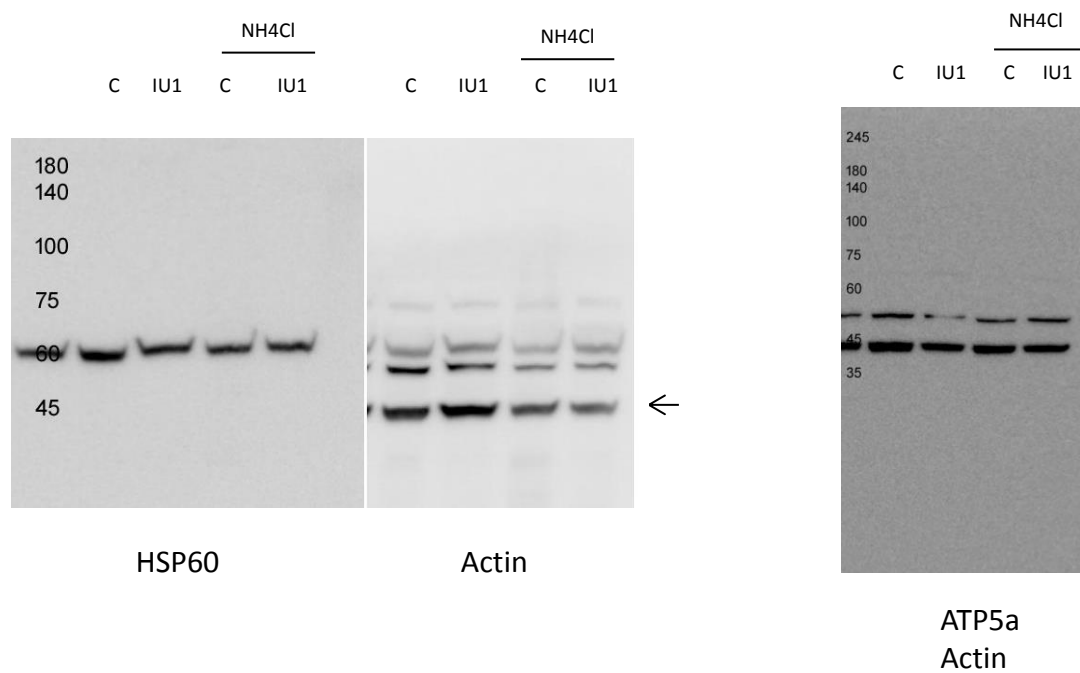

Fig S5B

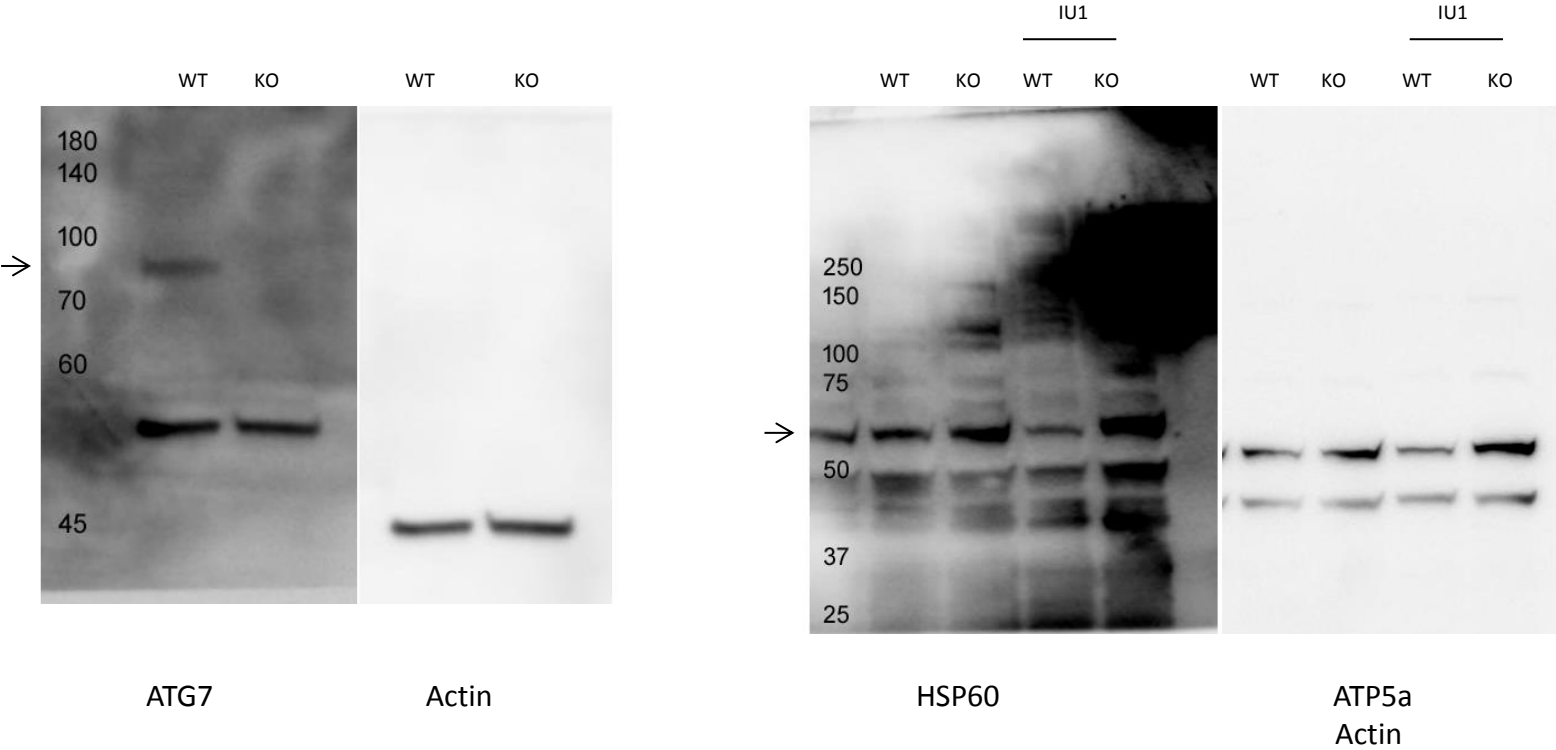

Fig S6A

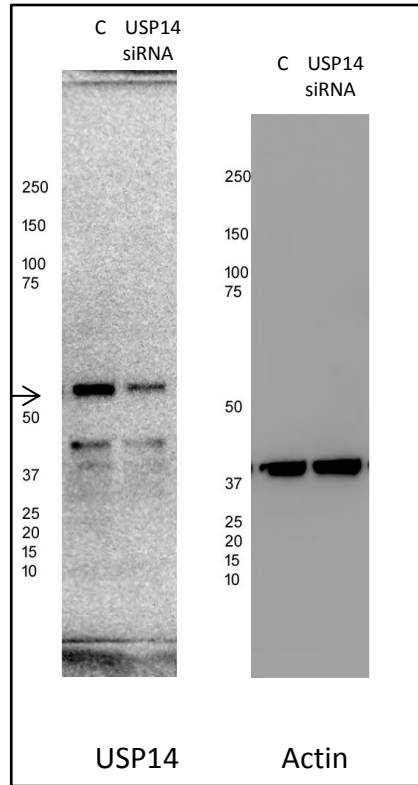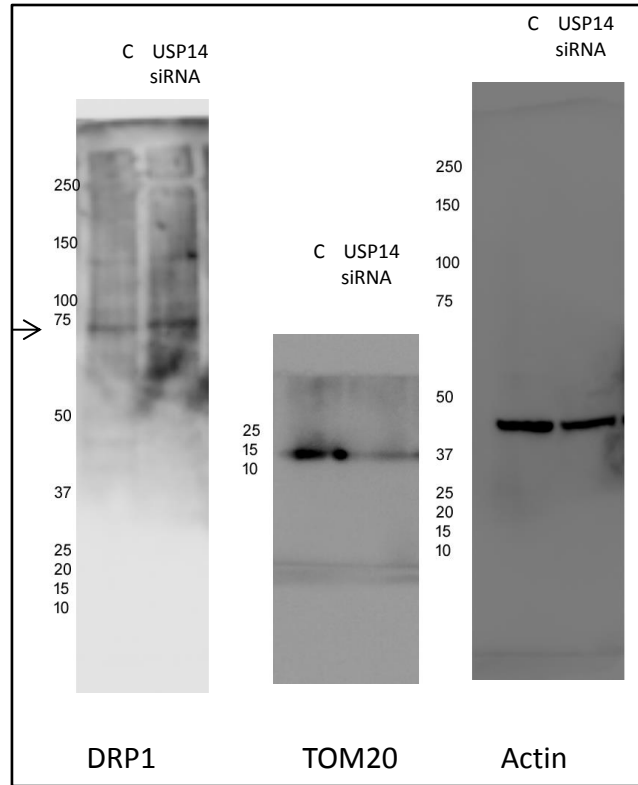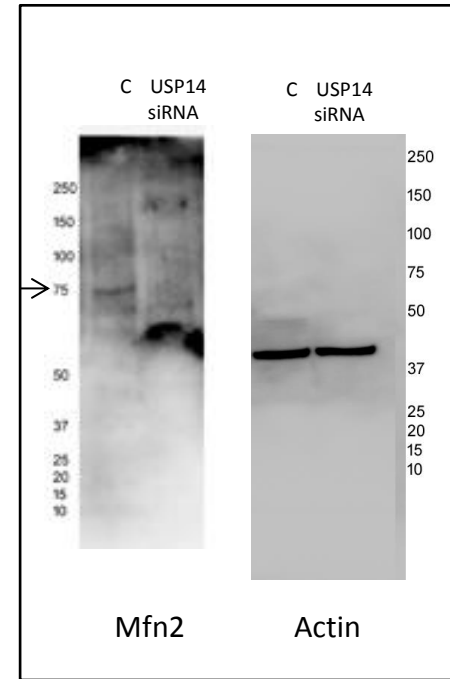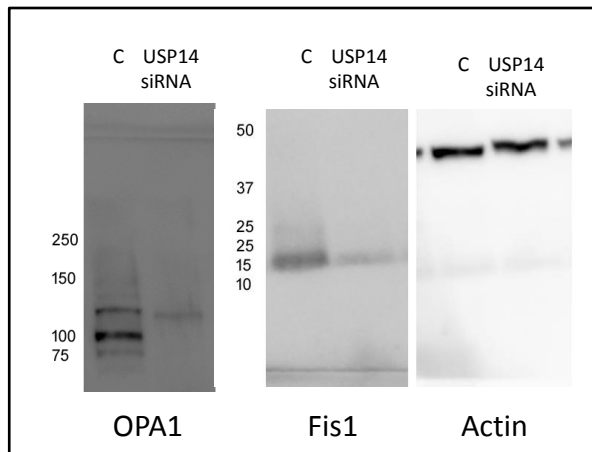

Fig S6C

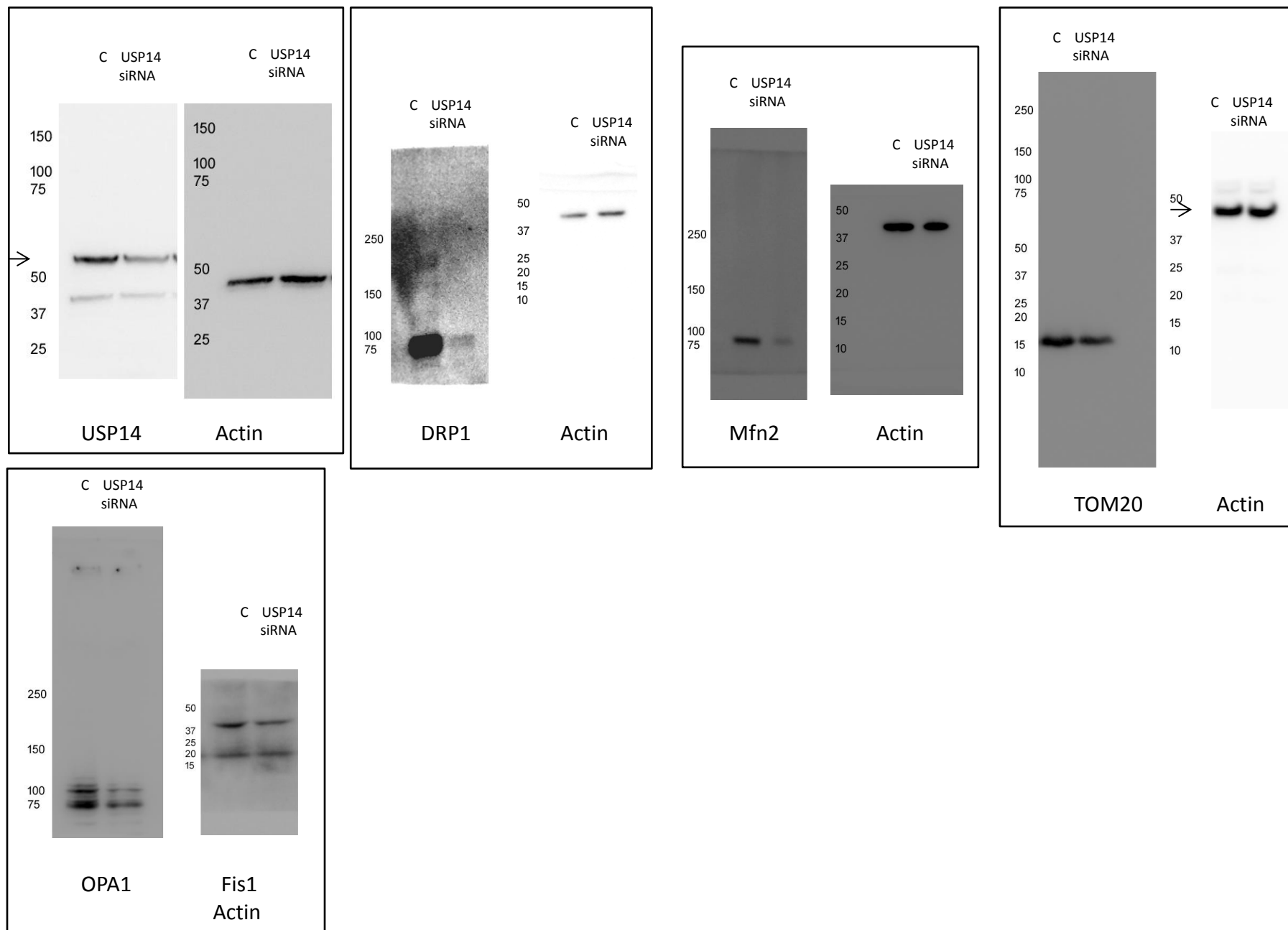

Fig S8C

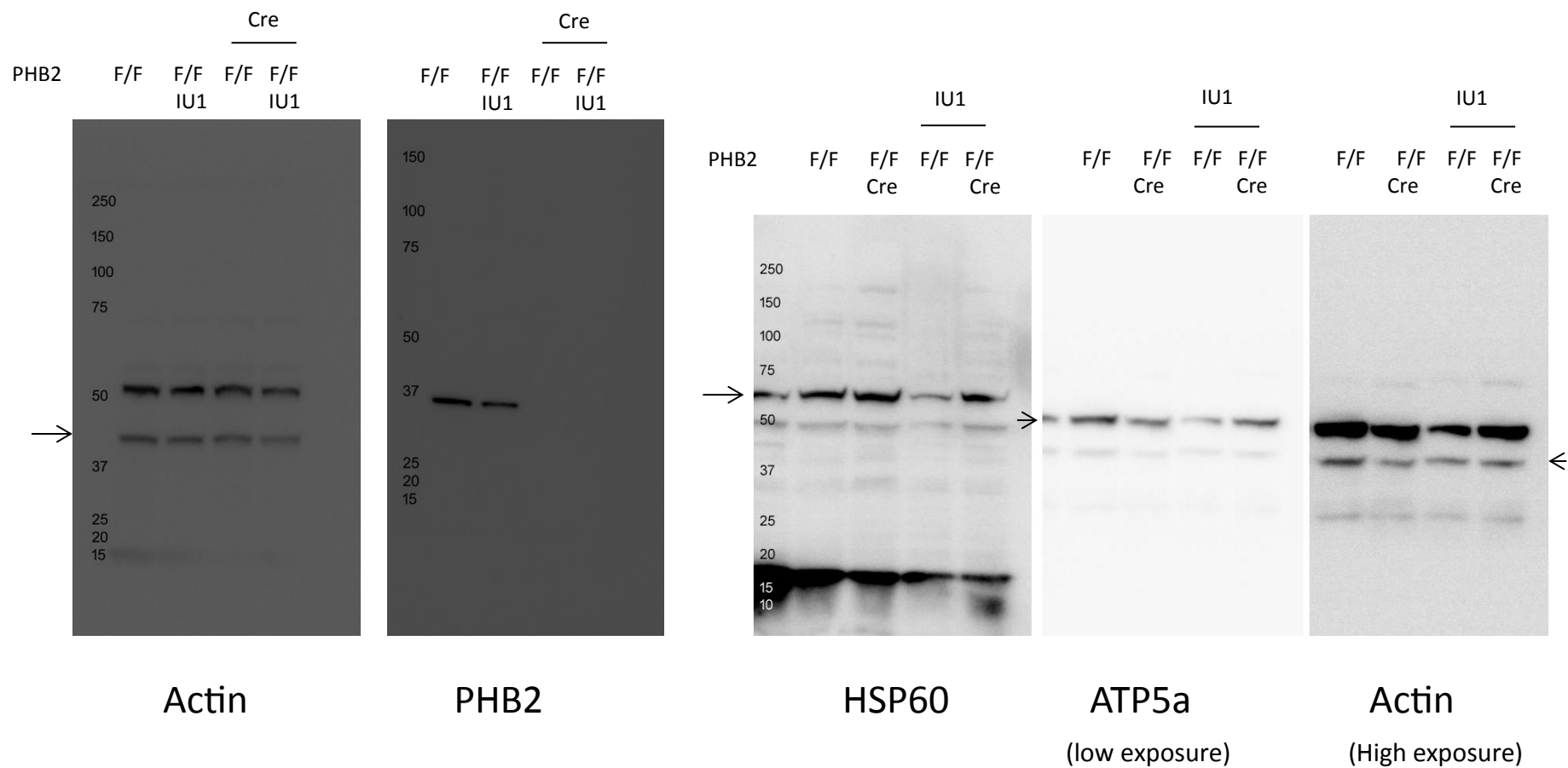

Fig S9

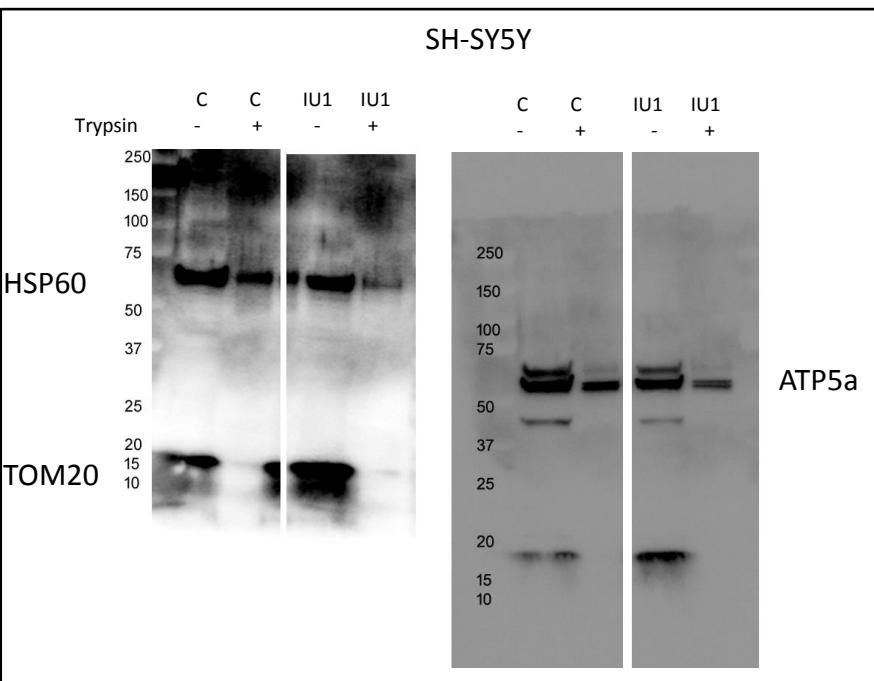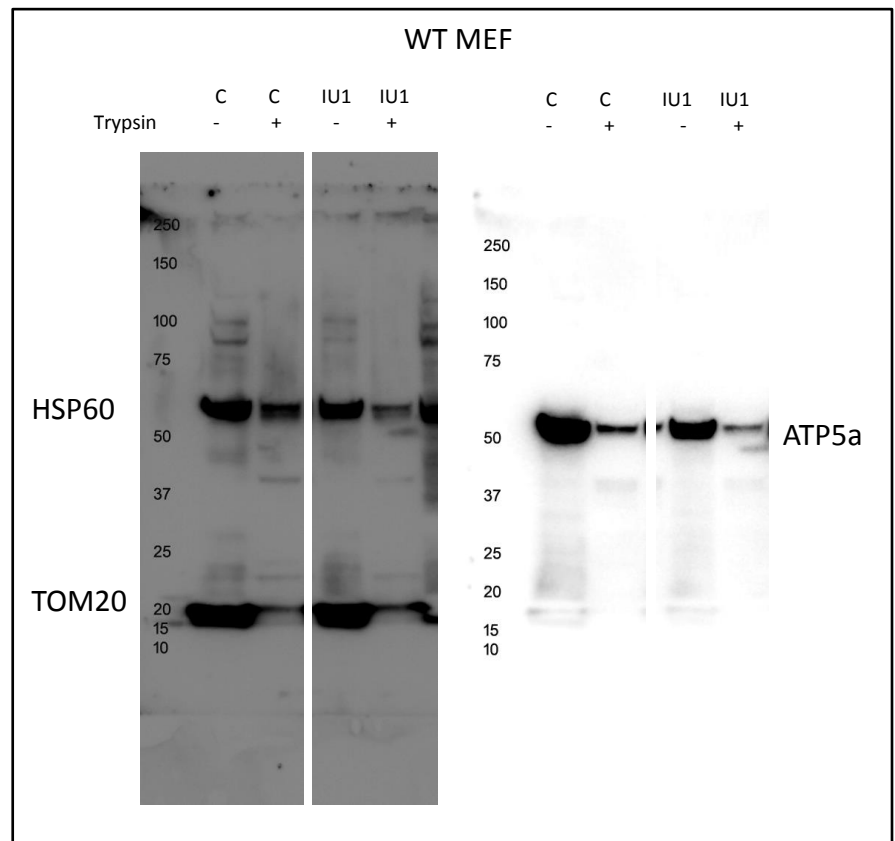

# PINK1 KO MEF

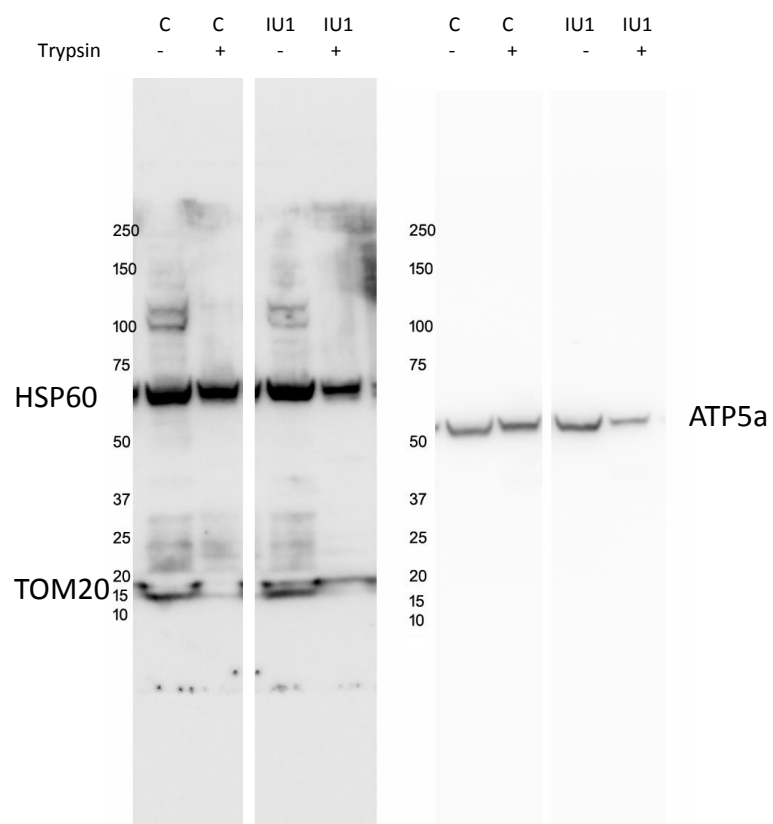

# Hela

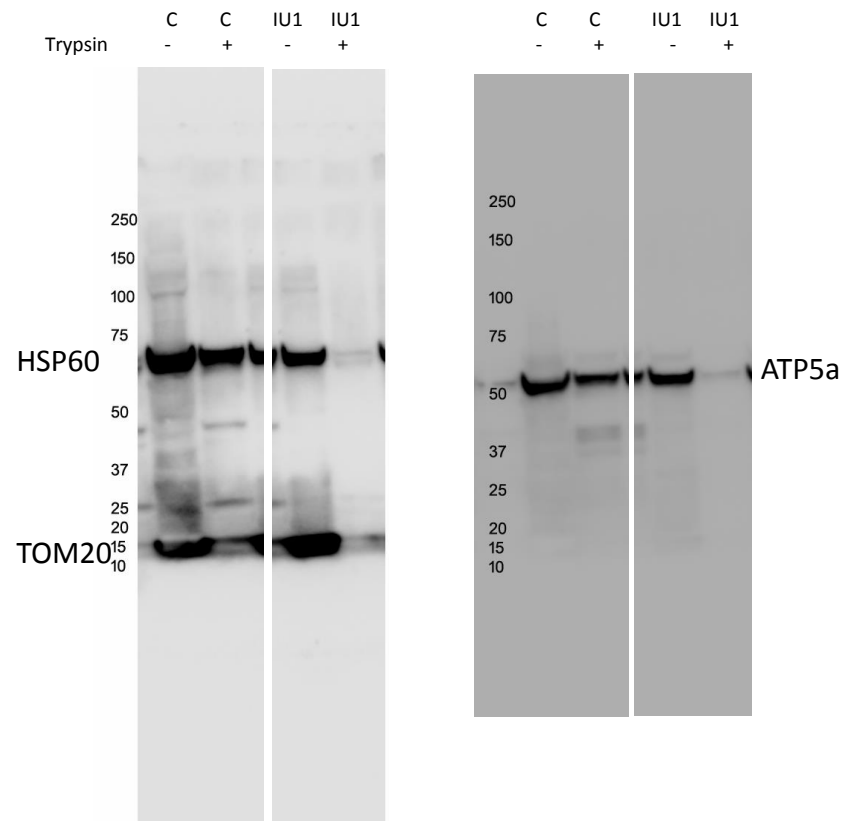

Supplement: Supplementary file 3 — Source Data for Appendix [file EMMM-10-e9014-s007.pdf]

Fig 1A

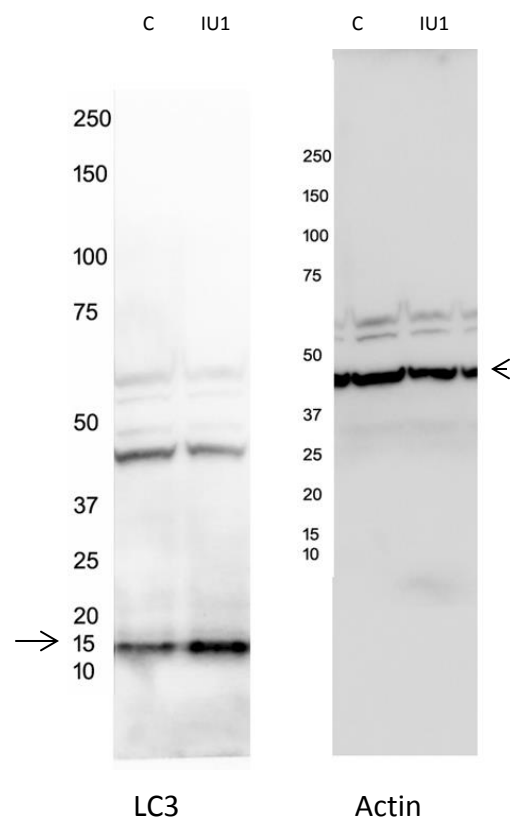

Fig 1E

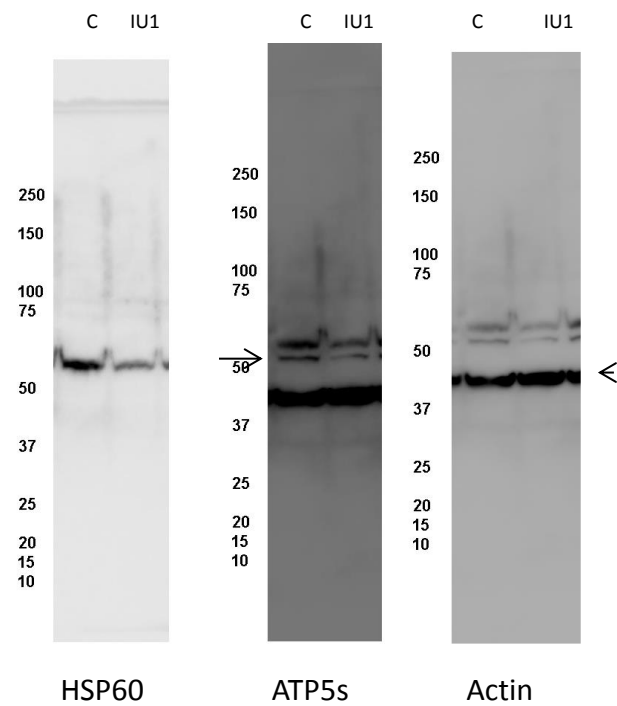

Supplement: Supplementary file 5 — Source Data for Figure 1 [file EMMM-10-e9014-s003.pdf]

Fig 2A

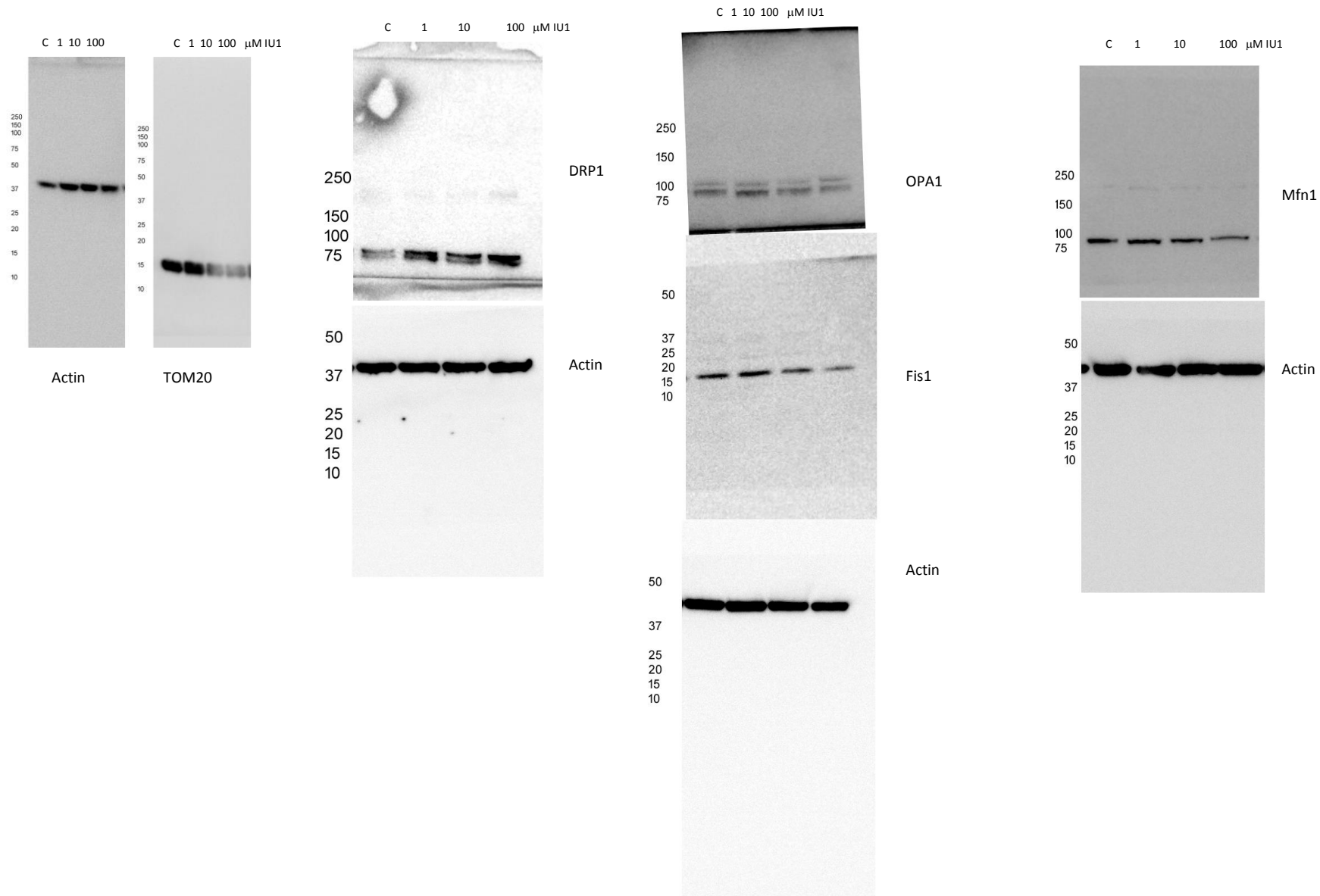

Fig 2A

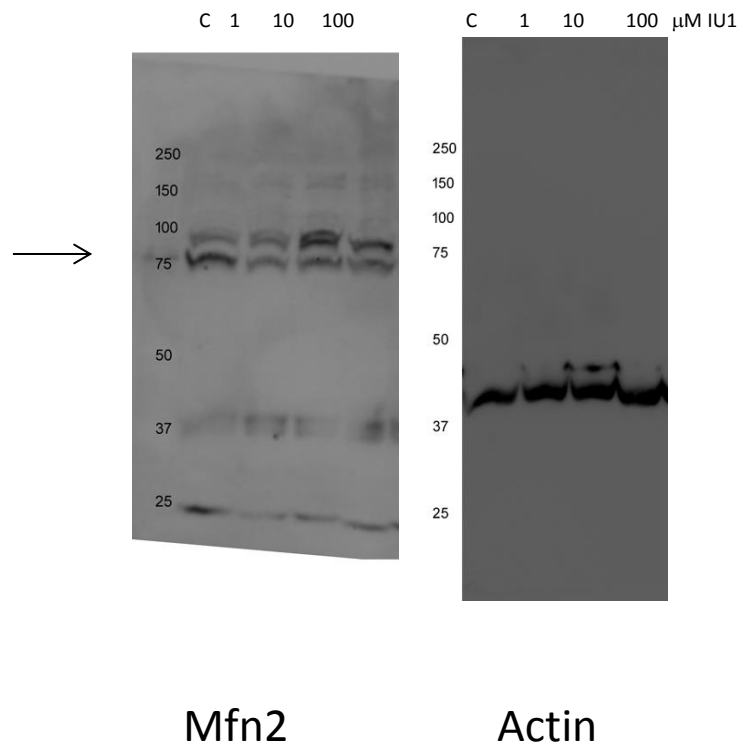

Fig 2E

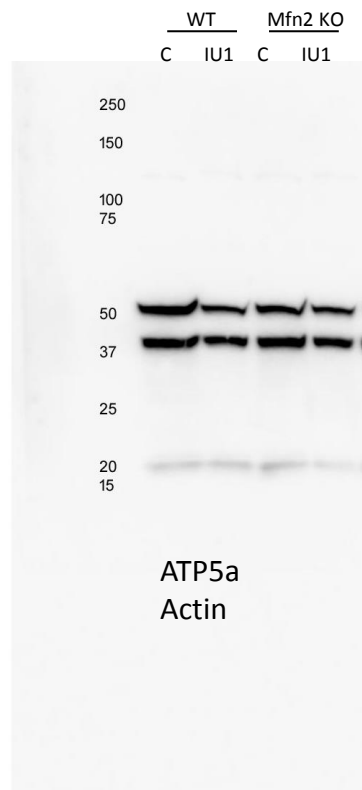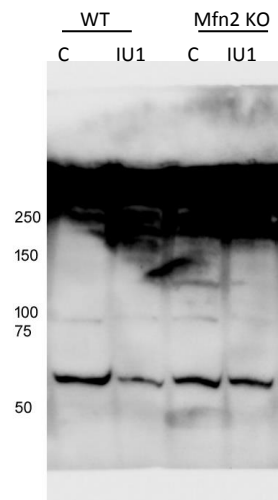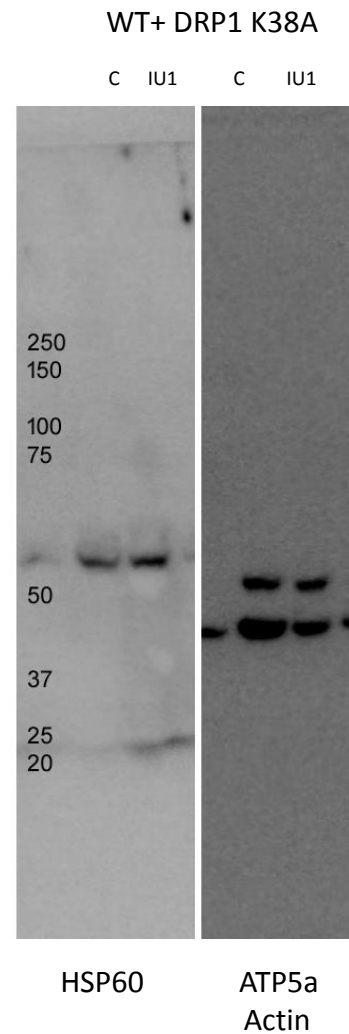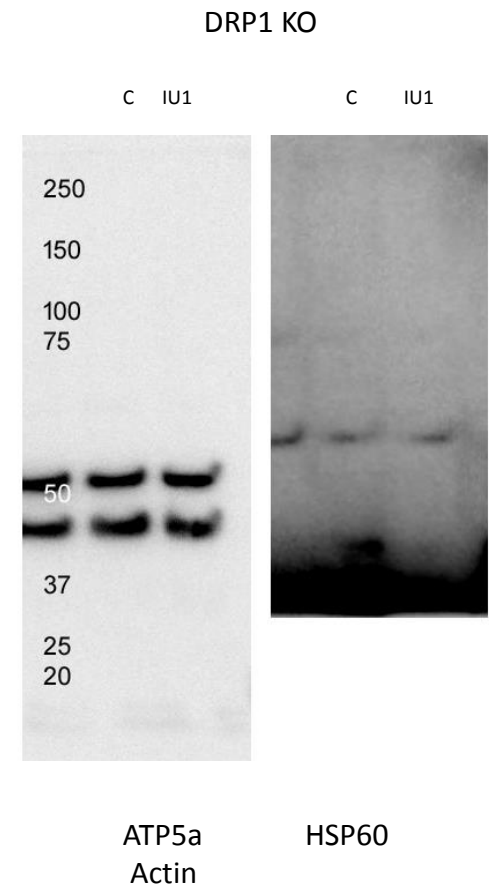

Supplement: Supplementary file 6 — Source Data for Figure 2 [file EMMM-10-e9014-s004.pdf]

Fig 5B

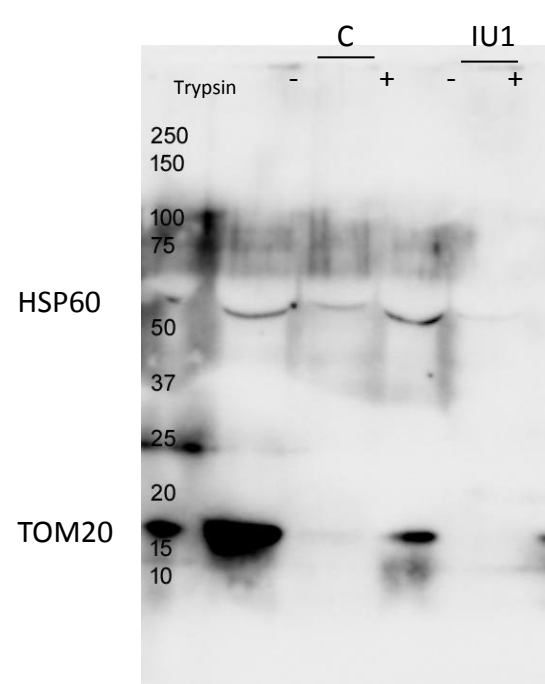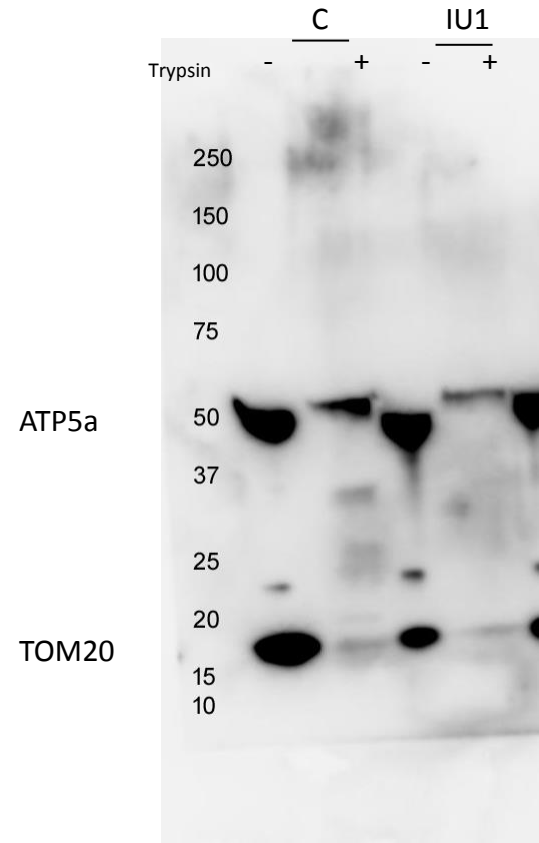

Fig 5D

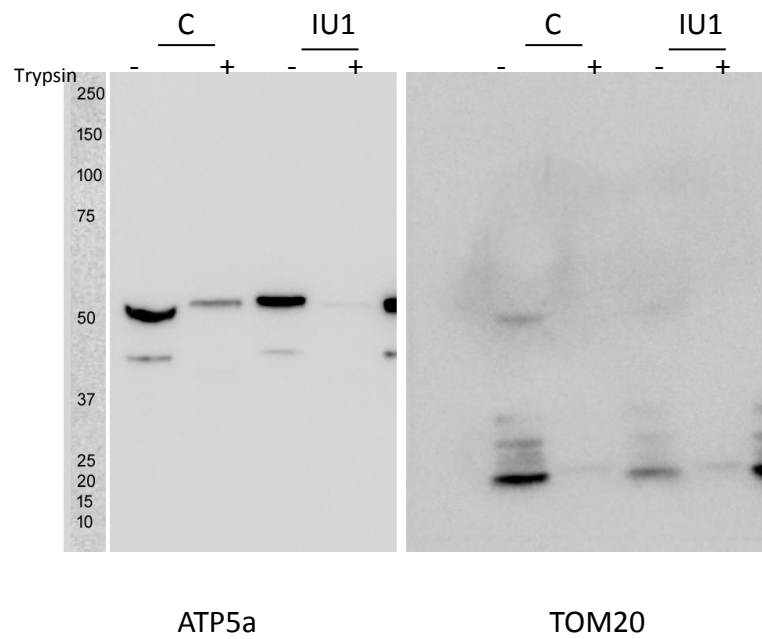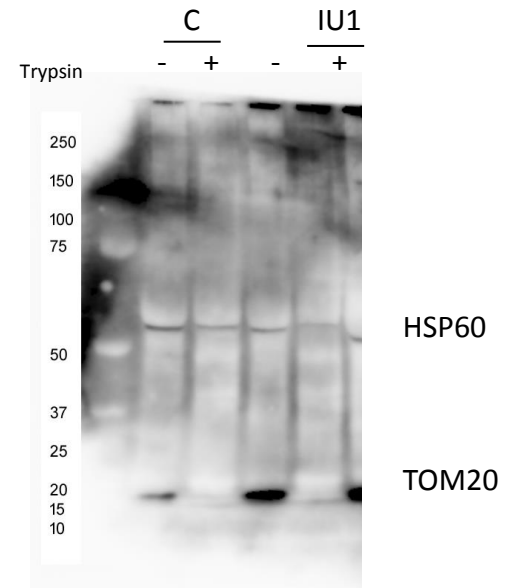

Fig 5F

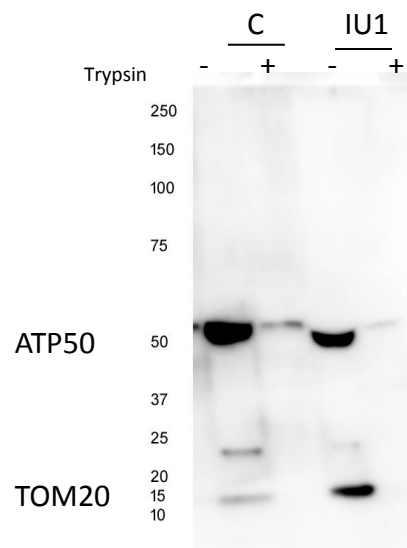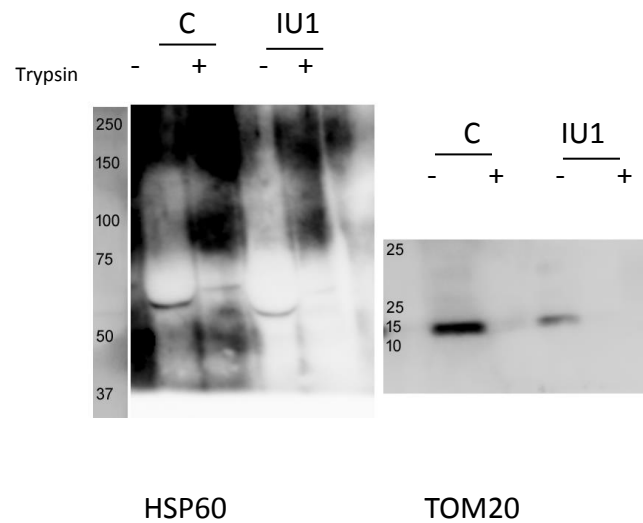

Fig 5H

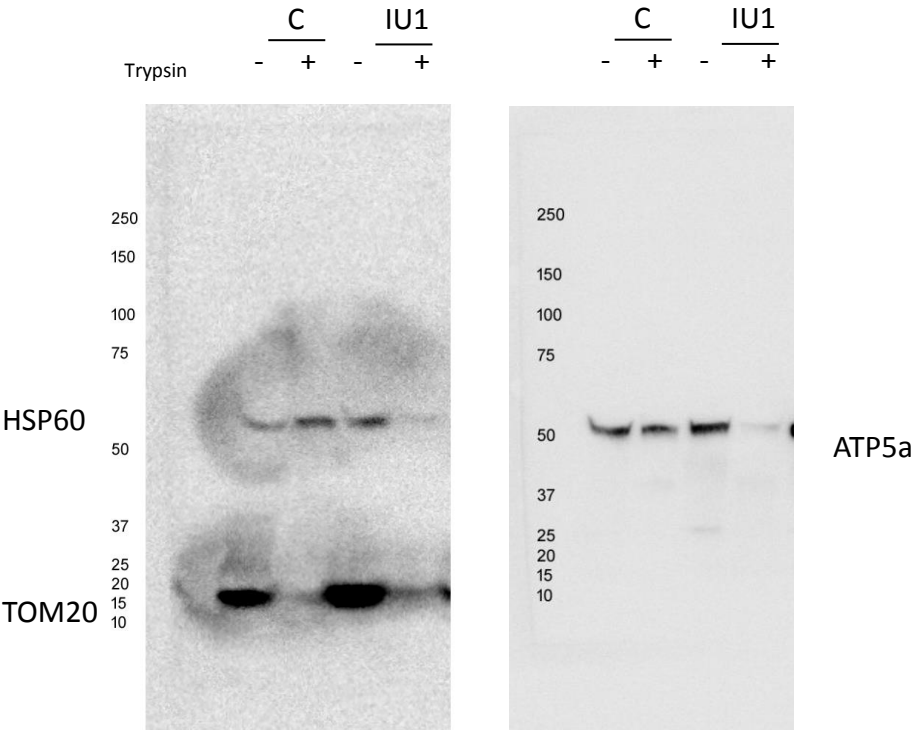

Fig 5J

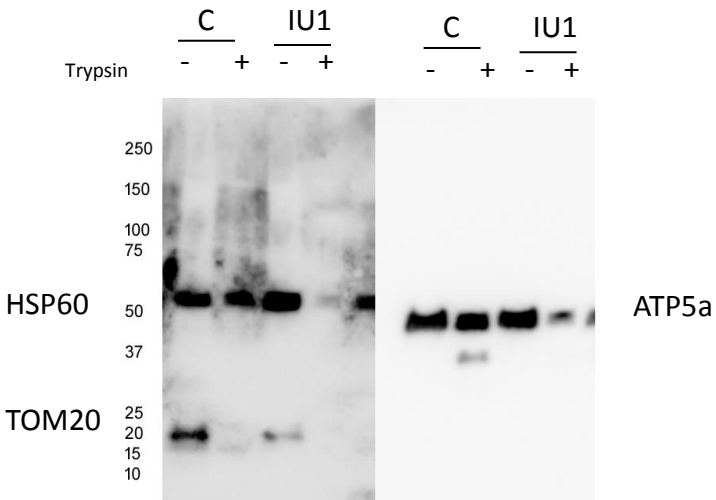

Supplement: Supplementary file 8 — Source Data for Figure 5 [file EMMM-10-e9014-s006.pdf]
