# Supplementary material for: USP14 inhibition corrects an in vivo model of impaired mitophagy
Source: EMBO Mol Med. 2018 Sep 24;10(11):e9014. doi: 10.15252/emmm.201809014 (PMC6220287; doi:10.15252/emmm.201809014)
Supplement: Supplementary file 7 — Source Data for Figure 3 [file EMMM-10-e9014-s005.pdf]

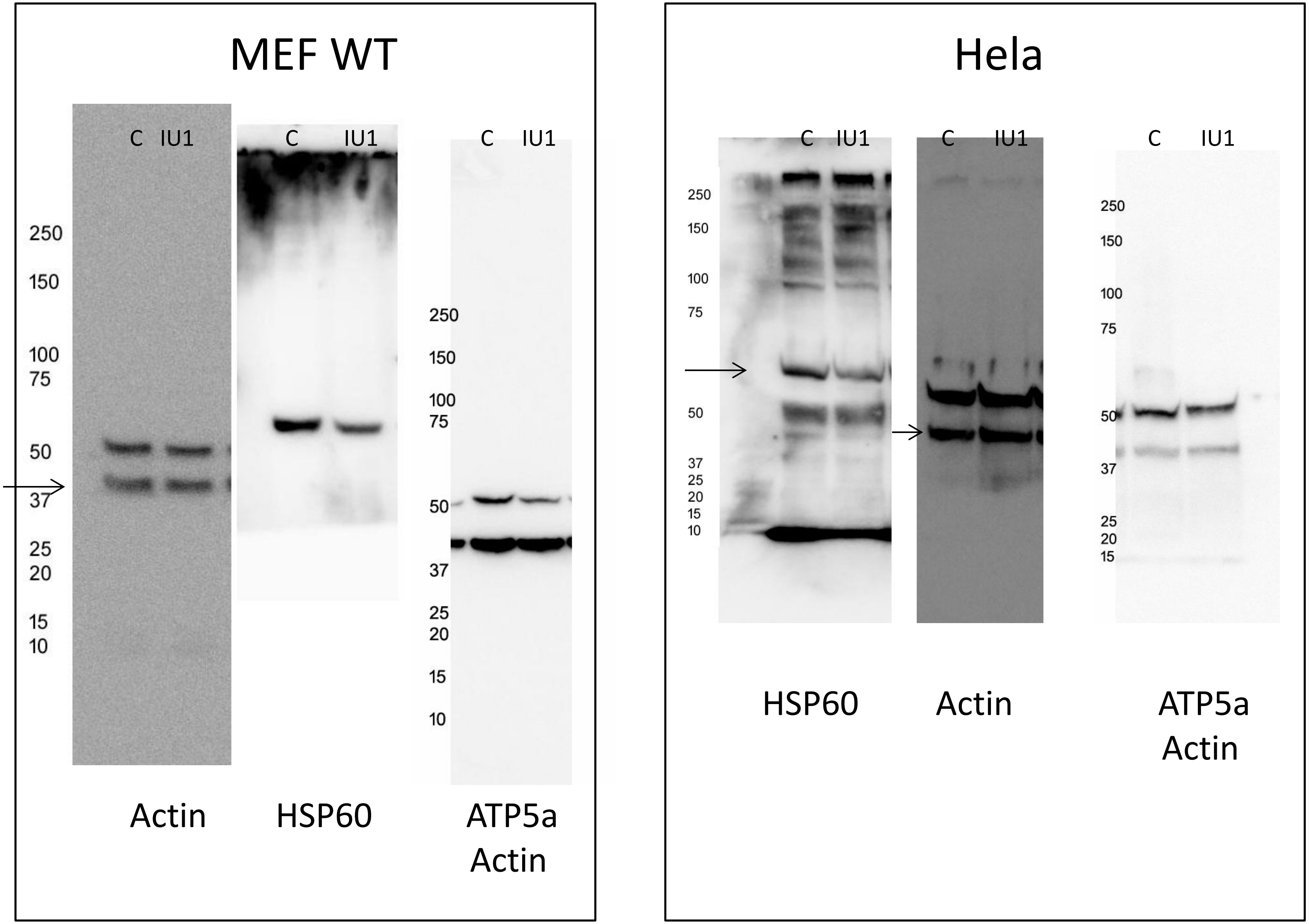
MEF WT
C IU1
C IU1
C IU1
250
150
100
75
50
37
25
20
15
10
Actin
HSP60
ATP5a
Actin
Hela
C IU1
C IU1
C IU1
250
150
100
75
50
37
25
20
15
10
HSP60
Actin
ATP5a
Actin

PINK1 KO

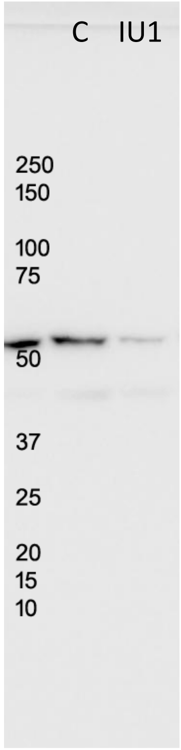

ATP5a

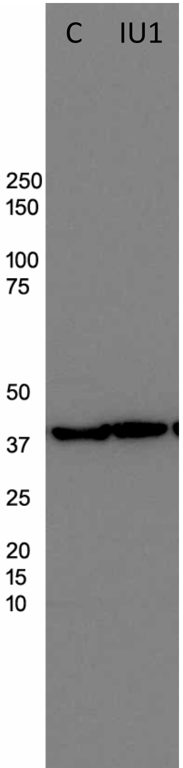

Actin

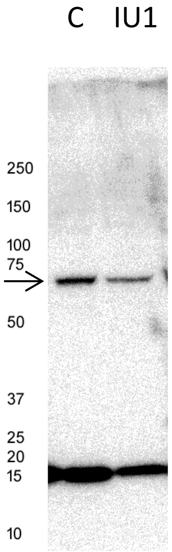

HSP60

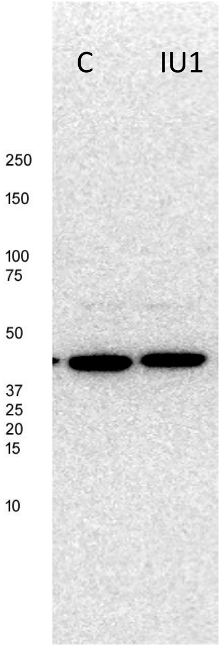

Actin

Control human

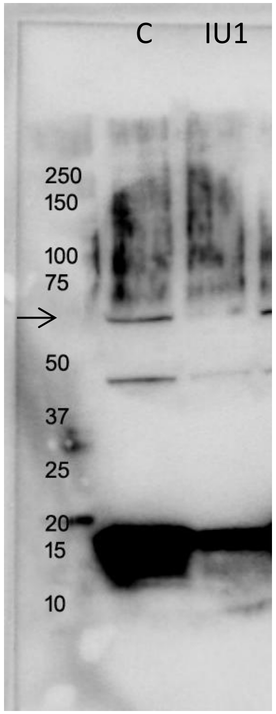

HSP60

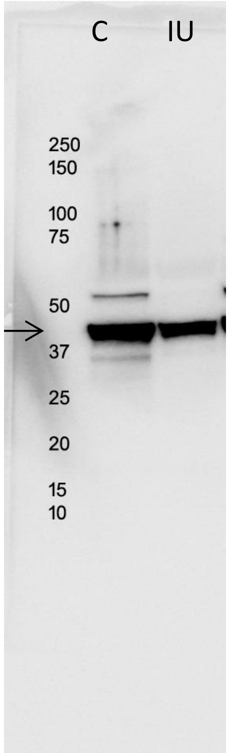

Actin

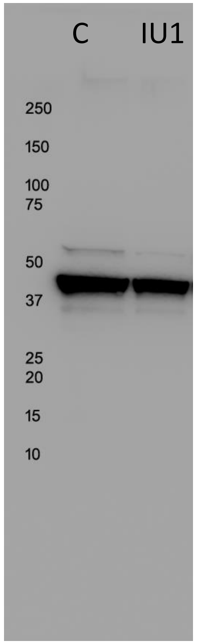

ATP5a
Actin

PD patient

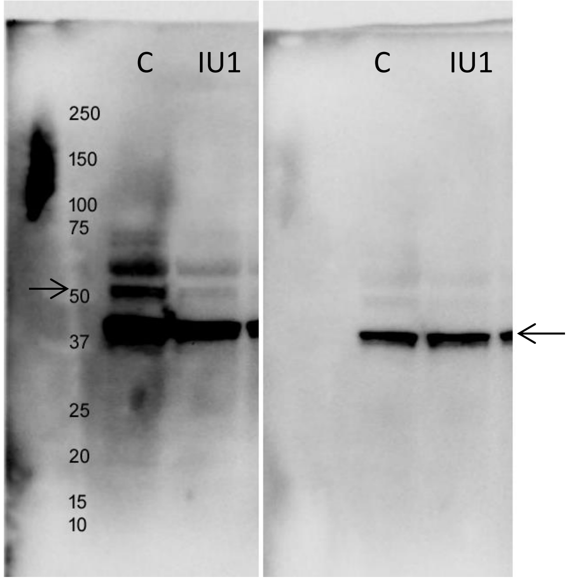

ATP5a

Actin

(Low exposure)

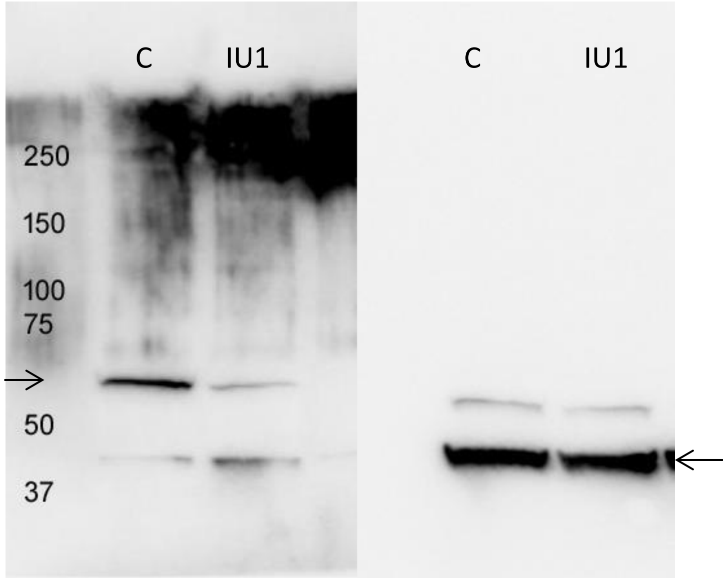

HSP60

Actin
