# Supplementary material for: USP14 inhibition corrects an in vivo model of impaired mitophagy
Source: EMBO Mol Med. 2018 Sep 24;10(11):e9014. doi: 10.15252/emmm.201809014 (PMC6220287; doi:10.15252/emmm.201809014)
Supplement: Supplementary file 4 — Review Process File [file EMMM-10-e9014-s008.pdf]

EMBO
Molecular Medicine

USP14 inhibition corrects an *in vivo* model of impaired mitophagy

Joy Chakraborty, Sophia von Stockum, Elena Marchesan, Federico Caicci, Vanni Ferrari, Aleksandar Rakovic, Christine Klein, Angelo Antonini, Luigi Bubacco and Elena Ziviani

Review timeline:

Submission date:	2 March 2018
Editorial decision:	12 April 2018
Revision received:	18 June 2018
Editorial decision:	11 July 2018
Revision received:	04 August 2018
Editorial decision:	13 August 2018
Revision received:	22 August 2018
Accepted:	24 August 2018

Editor: Céline Carret

Transaction Report:

(Note: With the exception of the correction of typographical or spelling errors that could be a source of ambiguity, letters and reports are not edited. The original formatting of letters and referee reports may not be reflected in this compilation.)

1st Editorial Decision 12 April 2018

Thank you for the submission of your manuscript to EMBO Molecular Medicine. We have now heard back from the two referees whom we asked to evaluate your manuscript.

You will see that both referees find the study interesting and convincing, with great prospects for PD therapeutic applications. While ref1 suggests a couple of experiments to strengthen the data and requests a more thorough job at referencing past literature, ref2 is a more critical. This referee regrets that the autophagy assays are under developed, statistical analyses are missing in place, more explanations are needed and provides a detailed listing of technical amendments to perform.

We would welcome the submission of a revised version within three months for further consideration and would like to encourage you to address all the criticisms raised as suggested to improve conclusiveness and clarity. Please note that EMBO Molecular Medicine strongly supports a single round of revision and that, as acceptance or rejection of the manuscript will depend on another round of review, your responses should be as complete as possible.

EMBO Molecular Medicine has a "scooping protection" policy, whereby similar findings that are published by others during review or revision are not a criterion for rejection. Should you decide to submit a revised version, I do ask that you get in touch after three months if you have not completed it, to update us on the status.

Please also contact us as soon as possible if similar work is published elsewhere. If other work is published we may not be able to extend the revision period beyond three months.

Please read below for important editorial formatting and consult our author's guidelines for proper formatting of your revised article for EMBO Molecular Medicine.

I look forward to receiving your revised manuscript.

© European Molecular Biology Organization

***** Reviewer's comments *****

Referee #1 (Remarks for Author):

In this study, Chakraborty et al discover a role of deubiquitinating enzyme USP14 in mitophagy. They find that USP14 is an inhibitor of basal mitophagy in mammalian cells as well as in drosophila. Consequently, knockdown of USP14, or alternatively pharmacological inhibition of USP14 with a small-molecule inhibitor of this enzyme, is manifestly beneficial to flies carrying mutation in the PINK1 or Parkin genes. For example, they see a major extension of the lifespan of these flies and well as improvement in indices of their health. It is logical that animals lacking in quality control mitophagy should particularly benefit from an enhancement of basal mitophagy, which is equally capable of clearing out defective mitochondria, just not selectively (or maybe not as selectively). The results are novel and quite surprising, and will be of interest to many readers. In addition, the results suggest a new potential approach for the treatment of Parkinson's disease. It is impressive that the authors have obtained such excellent data in a metazoan organism using a compound that is just an initial hit from a screen. In addition to all this, the paper is carefully done, so overall it is an unusually valuable contribution to the literature. It should stimulate many more studies in this area.

There are several open issues, whether or not they are beyond the scope of this paper. First, it is argued that the effect of USP14 is directed to basal mitophagy. Clearly it is, but does it only regulate basal mitophagy? It appears that the authors never explicitly tested a role in PINK1/Parkin-dependent mitophagy, which could be readily done. At least the discussion should better clarify that this is a question that remains to be addressed. Second, are the effects of USP14 on mitophagy and autophagy related, as the authors seem to favor? This is not explicitly addressed-namely, in the mutants that nicely abrogate the effect of USP14 on mitophagy (Drp1, Mfn2, Phb2), what happens to the effect of USP14 on autophagosome numbers and LC3? Is the entire stimulation of autophagy by USP14 a reflection of the stimulation of mitophagy?

Of course, despite the merits of the paper, the authors have not made any headway on the underlying mechanism by which USP14 suppresses mitophagy. However, it would be unrealistic to expect that in an initial paper (it could be a hard problem).

Some minor issues: in Fig 2E it would be better to show an additional blot, one for Drp1. In general, I think the referencing should to be improved. The general mitophagy references are mostly old (e.g., see line 92), and, in a field that moves extremely quickly, sort of outdated. There are excellent new (2018) reviews in Nat Rev Mol Cell Biol (19, 93) and Current Biology (28, R170), for example. Also the authors missed Nature 532, 398 (2016), which defines the many of the current paradigms of how USP14 acts.

Line 111: "To standardize a dose of USP14 inhibitor IU1, which does not affect cell survivability, we incubated SH-SY5Y cells......." should read "To standardize a dose of USP14 inhibitor IU1, we incubated SH-SY5Y cells......."

Line 340: "Based on this rational, recently deubqitinating enzymes (DUB) emerged as alternative to antagonise Parkin/ Pink1 dependency for mitophagy....." should read "Based on this rationale, recently deubiquitinating enzymes (DUB) emerged as alternative to antagonise Parkin/ Pink1 dependency for mitophagy....."

Referee #2 (Remarks for Author):

This is an interesting manuscript that reports a role for pharmacological and genetic inhibition of a proteasome-associated enzyme, USP14, in enhancing mitophagy and improving disease in fly models of Parkison's disease that are deficient in Pink1 or Parkin. This role of USP14 inhibition in enhancing mitophagy in cells appears to be genetically independent of Pink1 and Parkin but does require the autophagy gene, ATG7 and the inner mitochondrial membrane mitophagy receptor PHB2.

© European Molecular Biology Organization

As the authors discuss, this work is of potential importance, both in terms of enhancing our understanding of the molecular mechanisms of mitophagy and in providing support for the concept that targeting USP14 might be beneficial in the treatment of Parkinson's disease or other disorders associated with impaired mitophagy.

The manuscript is generally well-written (although there are numerous English language writing errors throughout). The experimental design benefits from the dual use of pharmacological and genetic inhibition of USP14, the use of multiple different assays to characterize mitophagy, the genetic approaches to define genes essential for USP14 inhibition phenotypes, and the use of different mammalian cell types and two established fly models of Parkinson's disease. Net, the overall message is fairly convincing and of interest. However, the autophagy assays are significantly underdeveloped, statistical analyses are lacking in many places, and numerous technical concerns exist about many of the figures. If these concerns can be addressed with further experiments, this work could represent an important contribution to the field.

Major Comments:
1. The evidence that IU1 or USP14 siRNA increases autophagy is underdeveloped. Measurement of LC3-II alone is not sufficient to draw any conclusions. This could be due to translational effects on LC3 and/or a block in autophagic flux. The EM data provided in Fig. 1B-D and S3B-C do not address this deficiency, as the authors state they are measuring autophagosomes, which would increase if autophagic flux (i.e. autophagolysosomal maturation) were blocked. Having said this, it is not clear whether they are quantitating true autophagosomes or both autophagosomes and autolysosomes; from the images shown, I suspect the major of structures they are calling "autophagosomes" are actually autolysosomes. (This concern applies to all EM data throughout the manuscript.)

2. It is not clear why the authors chose to include data with the inhibitor IU1 in the main text and delegate the data with USP14 siRNA to the supplemental figures. Genetic data are arguably more important than pharmacological data for USP14 inhibition.

3. The experiment in Figure 2C-D is difficult to interpret, as mitochondrial volume appears to significantly decreased in the Drp1 KO, Mfn1 KO and Mfn2 KO MEFs compared to WT MEFs in the absence of IU1 treatment. These data do not convincingly prove the point that mitochondria need to fragment before undergoing mitophagy.

4. It is unclear whether the immunogold staining for the UPS 20S subunit is specific in Fig. 4C and S7C, as there appears to be immunogold particles in the interior of the intact mitochondrion shown in the lower left inset of scramble siRNA-treated cells in Fig. S7C. Although the EM findings are included primarily as corroboration of the light microscopic findings, these EM experiments need to be performed in a rigorous manner with extensive controls if they are to be included in the manuscript. The data currently suggests, if taken at face value (Fig. 7C) that in baseline conditions, the UPS 20S proteasome complex is located in the middle of intact mitochondria.

5. Although the authors discuss the data showing lack of toxicity of IU1, can they speculate on potential adverse consequences of USP14 inhibition - both in the context of non-mitophagy-related effects and the potential long-term consequences of the observed increased numbers of mitochondria with membrane rupture.

6. The statement beginning on line 442 is a bit misleading, claiming that this study is the first effort to fully characterize in vivo the biology of a proteasome-associated deubiquitinating enzyme in the context of mitophagy. Perhaps this is technically correct if USP14 is truly a specific "proteasome-associated" DUB and USP30 is a specific mitochondrial deubiquitinating enzyme. Nonetheless, the authors should acknowledge and discuss their findings in light of previous work such as the Bingol et al, Nature, 2014 study showing that inhibition of USP30 exerts similar beneficial effects on mitophagy and other outcomes in fly models of Parkinson's disease.

Minor Comments:
1. The manuscript is well-written but needs professional editing throughout for proper English language writing.

2. Figure S1A-B. The y-axis label is unclear. Fold-change of what? Also, MTT is not an acceptable readout of viability, as it measures cell proliferation and mitochondrial metabolism. This is a particular issue for a study focused on the use of the IU1 inhibitor to study effects on mitophagy. Net, cell viability should be assessed using a direct measure of cell survival that is independent of these other variables.

3. Figure S1C. For non-experts in Drosophila wing motor neuron morphology, these images are difficult to interpret. The IU1 100 micromolar image looks different than the others, but I do not know whether this is biologically real/meaningful or not. Is there are a more objective or quantitative method to assess whether this concentration of IU1 has effects on Drosophila wing motor morphology?

4. Figure S2A-B. The authors do not clearly explain the rationale for measuring chymotrypsin-like activity. More importantly, the effects of IU1 on this activity are not that striking. As the authors acknowledge the caveats of this assay to measure proteasome activity (lines 125-128) and then go on to use alternative assays, I think the data in Figure S1A-B detracts from the study and its removal from the manuscript could be considered.

5. Figure S2C-D. The figure would be clearer if the authors change "GFP" to "GFP-Ub."

6. Figure 1E. Although there is quantitation provided normalizing the mitochondrial protein levels to actin, it is notable that there is a significant decrease in the Actin loaded on the gel beneath ATP5a in the IU1-treated group. This uneven loading for actin raises concerns about the validity of the conclusion that ATP5a levels are decreased.

7. Figure S3. The authors need to show western blots or RT-PCR to confirm that the USP14 siRNA is truly knocking down USP14.

8. Figure S4. As above, I think the authors are incorrectly calling autolysosomes (or autophagolysosomes) autophagosomes. The arrowhead in S4B convincingly shows a mitochondria inside an autophagic structure. However, in S4A, the arrowhead does not convincingly show a mitochondrion. The structure delineated by the arrowhead is in too advanced a stage of degradation to discern whether it is or is not a mitochondrion.

9. Figure S5A. This figure is not interpretable. The authors need to assess LC3 (and preferably also p62) in cell treated with control or IU1 in the presence or absence of IU1 in the same experiment. i.e. while there is more LC3-II in the IU1-treated cells than the control cells, it is not possible to determine whether there is an increase in LC3-II upon chloroquine and IU1 treatment versus IU1 treatment alone. This speaks to the central question raised above of whether the data convincingly show that IU1 increases autophagic flux (or rather, blocks, autophagosomal maturation). Also, for HSP60 and ATP5a levels, it is important to compare no chloroquine treatment and chloroquine treatment conditions in the same experiment (gel).

10. Lines 178-179. It is unclear what the authors mean by "mitochondrial shape and size are primary prerequisites for mitophagy";'

11. Figure S2B. The results are graphed as "normalized by actin". However, it is unclear what a value of 1.0 means, as the control levels for each protein are not 1.0. This type of "normalization" is unclear and not standard.

12. Figure S6A. The gels for TOM20 and OPA1 are so dark that it is hard to see specific bands. Better quality gels are required.

13. Figure S8C. In the gel shown, it appears that HSP60 (but not ATP55a) is reduced in the PHB2 flox/flox + cre group versus the PHB2 flox/flox group. There are no statistical analyses of this comparision in the graph in Fig. D to see if this is significant, but the data in the gel seem inconsistent with PHB2 playing a role in mitophagy and make it hard to assess whether PHB2 is required for IU1-induced mitophagy, as the levels of HSP60 already appear lower in the PHB2 deleted cells without IU1 treatment. Thus, the lack of a further decrease with IU1 treatment are difficult to interpret.

© European Molecular Biology Organization

14. Figure S10A. The precise p-value calculated by a log-rank test should be provided for these survival analyses. Similarly, statistics are missing for Figure S10B-C.

15. Figure 6A. The red color of the lines for the Pink1 KO and the Pink1 KO/USP14 KD appear quite similar. The color of one of these lines should be changed so the reader can more easily appreciate what genotype corresponds to what survival curve. The same concern applies to Figure 7A.7

16. Figure 6E. I am not sure "dark mitochondria" and "white mitochondria" are standard terms in the field to describe the different mitochondrial morphological phenotypes.

1st Revision - authors' response 18 June 2018

Referee #1 (Remarks for Author):

In this study, Chakraborty et al discover a role of deubiquitinating enzyme USP14 in mitophagy. They find that USP14 is an inhibitor of basal mitophagy in mammalian cells as well as in drosophila. Consequently, knockdown of USP14, or alternatively pharmacological inhibition of USP14 with a small-molecule inhibitor of this enzyme, is manifestly beneficial to flies carrying mutation in the PINK1 or Parkin genes. For example, they see a major extension of the lifespan of these flies and well as improvement in indices of their health. It is logical that animals lacking in quality control mitophagy should particularly benefit from an enhancement of basal mitophagy, which is equally capable of clearing out defective mitochondria, just not selectively (or maybe not as selectively). The results are novel and quite surprising, and will be of interest to many readers. In addition, the results suggest a new potential approach for the treatment of Parkinson's disease. It is impressive that the authors have obtained such excellent data in a metazoan organism using a compound that is just an initial hit from a screen. In addition to all this, the paper is carefully done, so overall it is an unusually valuable contribution to the literature. It should stimulate many more studies in this area.

We are very grateful to the reviewer for the careful analysis of our paper and for the appreciation of our work.

There are several open issues, whether or not they are beyond the scope of this paper. First, it is argued that the effect of USP14 is directed to basal mitophagy. Clearly it is, but does it only regulate basal mitophagy? It appears that the authors never explicitly tested a role in PINK1/Parkin-dependent mitophagy, which could be readily done. At least the discussion should better clarify that this is a question that remains to be addressed.

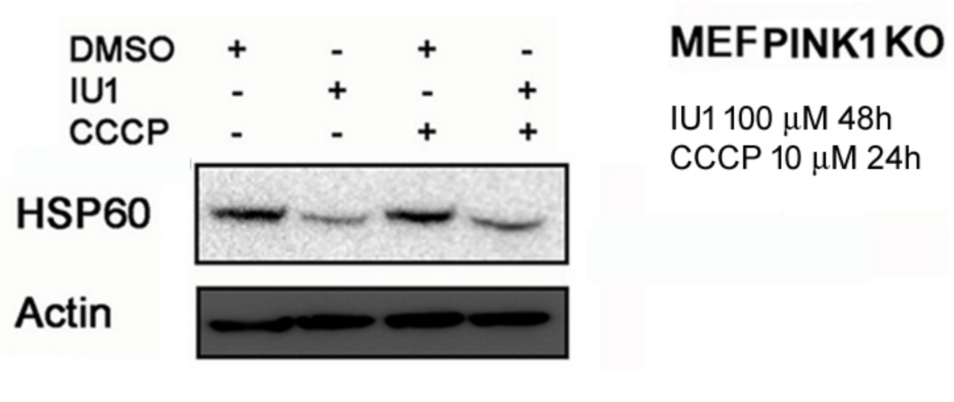

We are thankful to the reviewer for giving us the opportunity to clarify this aspect. We have initially assessed whether USP14 inhibition can elevate CCCP induced mitophagy in PINK1 KO MEFs. The difference in mitophagy between IU1 treated and IU1+CCCP treated PINK1 KO cells was found statistically insignificant (for reviewer's consideration Fig 1). There might be several explanations for this lack of effect. It is possible that CCCP treatment interferes with USP14 induced mitophagy.

For instance, we have found that CCCP in SH-SY5Y cells can reduce USP14 protein levels (for reviewer's consideration Fig 2). Therefore it is possible that IU1 inhibition might not have sufficient USP14 to block, and further boost mitophagy. Another possibility is that in other systems CCCP intoxication results in maximal mitophagy so that the synergistic effect of IU1 and CCCP cannot be revealed and/or it cannot be analysed against the background because it is below the threshold of the detecting technique.

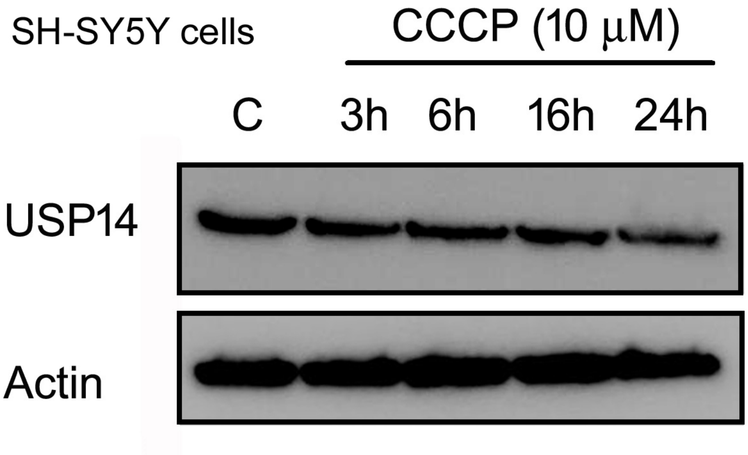

There can be many speculations, and we agree with the reviewer that this is an issue that remains open for further investigations.

Second, are the effects of USP14 on mitophagy and autophagy related, as the authors seem to favor? This is not explicitly addressed-namely, in the mutants that nicely abrogate the effect of USP14 on mitophagy (Drp1, Mfn2, Phb2), what happens to the effect of USP14 on autophagosome numbers and LC3? Is the entire stimulation of autophagy by USP14 a reflection of the stimulation of mitophagy?

Indeed mitophagy and autophagy are always interconnected, but how do they crosstalk is still a matter of debate. The effect of USP14 inhibition on autophagy has been well characterised by a number of independent studies (Boselli et al, 2017; Xu et al, 2016). Our main aim here was to assess whether this elevated autophagy is also accompanied with enhanced mitophagy, and if so whether we can use this for therapeutic interventions. We used DRP1, Mfn1, Mfn2 KO and PhB2 $^{F/F}$ cells to stress the point that mitochondrial morphology and LC3-receptor are essential prerequisites for USP14 mediated mitophagy. In our opinion dissecting the autophagic flux in each of these cell lines and in combination with USP14 inhibition, is beyond the scope of this work.

It also has to be mentioned that previous works have shown that the autophagic flux is not affected in DRP1 and Mfn2 KO MEF cells (Gomes et al, 2011) and LC3 levels remains unaffected after Phb2 knockdown (Wei et al, 2017), which minimises the possibility that impaired autophagosome formation might largely contribute to the lack of effect of IU1 in this backgrounds. As previously reported by Gomes et al., we also found that the autophagic flux measured in presence of chloroquine was comparable in both KO background (for reviewer's consideration Fig 3, upper panel) and that IU1 treatment increases LC3 levels in Mfn2 as well as DRP1 KO background (for reviewer's consideration Fig 3, lower panel).

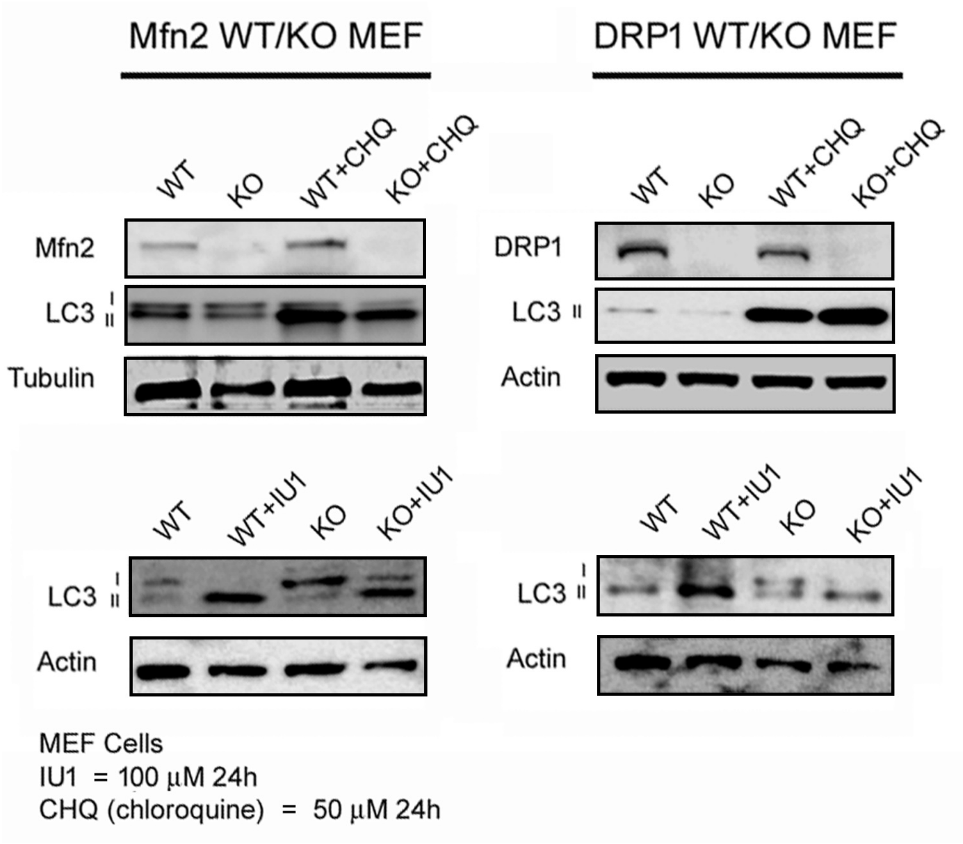

Further studies are required to clearly dissect mitophagy /autophagy interplay in these cell lines and in combination with USP14 inhibition.

Of course, despite the merits of the paper, the authors have not made any headway on the underlying mechanism by which USP14 suppresses mitophagy. However, it would be unrealistic to expect that in an initial paper (it could be a hard problem).

Indeed this is not a trivial question and requires further investigation. This study shows the novel finding that enhanced autophagy accompanied with increased proteasome activity and mitochondrial rupture leads to USP14 inhibition induced mitophagy. Currently we are trying to address which part of mitochondria is specifically targeted, and which mitochondrial protein population is controlled by USP14. In this respect mass spectrometry analysis is currently under evaluation for identifying novel USP14 targets and further studies will follow.

Some minor issues:
In Fig 2E it would be better to show an additional blot, one for Drp1.

We have modified the image and replaced the blots.

In general, I think the referencing should to be improved. The general mitophagy references are mostly old (e.g., see line 92), and, in a field that moves extremely quickly, sort of outdated. There are excellent new (2018) reviews in Nat Rev Mol Cell Biol (19, 93) and Current Biology (28, R170), for example. Also the authors missed Nature 532, 398 (2016), which defines the many of the current paradigms of how USP14 acts.

We thank the reviewer for this valid suggestion. We have included updated references in the text.

Line 111: "To standardize a dose of USP14 inhibitor IU1, which does not affect cell survivability, we incubated SH-SY5Y cells......." should read "To standardize a dose of USP14 inhibitor IU1, we incubated SH-SY5Y cells......."

We have modified the sentence according to the reviewer's suggestion.

Line 340: "Based on this rational, recently deubquitinating enzymes (DUB) emerged as alternative to antagonise Parkin/ Pink1 dependency for mitophagy....." should read "Based on this rationale, recently deubiquitinating enzymes (DUB) emerged as alternative to antagonise Parkin/ Pink1 dependency for mitophagy....."

We have modified the sentence according to the reviewer's suggestion.

Referee #2 (Remarks for Author):

This is an interesting manuscript that reports a role for pharmacological and genetic inhibition of a proteasome-associated enzyme, USP14, in enhancing mitophagy and improving disease in fly models of Parkison's disease that are deficient in Pink1 or Parkin. This role of USP14 inhibition in enhancing mitophagy in cells appears to be genetically independent of Pink1 and Parkin but does require the autophagy gene, ATG7 and the inner mitochondrial membrane mitophagy receptor PHB2.

As the authors discuss, this work is of potential importance, both in terms of enhancing our understanding of the molecular mechanisms of mitophagy and in providing support for the concept that targeting USP14 might be beneficial in the treatment of Parkinson's disease or other disorders associated with impaired mitophagy.

The manuscript is generally well-written (although there are numerous English language writing errors throughout). The experimental design benefits from the dual use of pharmacological and genetic inhibition of USP14, the use of multiple different assays to characterize mitophagy, the genetic approaches to define genes essential for USP14 inhibition phenotypes, and the use of different mammalian cell types and two established fly models of Parkinson's disease. Net, the overall message is fairly convincing and of interest. However, the autophagy assays are significantly underdeveloped, statistical analyses are lacking in many places, and numerous technical concerns exist about many of the figures. If these concerns can be addressed with further experiments, this work could represent an important contribution to the field.

We are very grateful to the reviewer for the careful analysis of our paper and for the appreciation of our work.

Major Comments:

1. The evidence that IU1 or USP14 siRNA increases autophagy is underdeveloped. Measurement of LC3-II alone is not sufficient to draw any conclusions. This could be due to translational effects on LC3 and/or a block in autophagic flux. The EM data provided in Fig. 1B-D and S3B-C do not address this deficiency, as the authors state they are measuring autophagosomes, which would increase if autophagic flux (i.e. autophagolysosomal maturation) were blocked. Having said this, it is not clear whether they are quantitating true autophagosomes or both autophagosomes and autolysosomes; from the images shown, I suspect the major of structures they are calling "autophagosomes" are actually autolysosomes. (This concern applies to all EM data throughout the manuscript.)

Previous independent studies provided detailed description that USP14 promotes the autophagic flux, mainly through Beclin1 dependent pathway. The mechanism demonstrates that Akt facilitates USP14 to negatively regulate K63 ubiquitination of Beclin1. Beclin1-K63 ubiquitination is necessary for Beclin1-ATG14L-Vps34 complex formation, which is involved in activation of autophagy and autophagosome maturation (Boselli et al, 2017; Xu et al, 2016). The aim of this study was not to reproduce the previously available data on autophagy, but rather investigate whether this elevated autophagy is accompanied with increased mitophagy, which was novel. We thought it was best to start from reproducing in our model system the established fact that genetic interference or pharmacologic inhibition of USP14 leads to increased autophagy by measuring LC3 levels. To this aim, we treated cells with IU1 in combination with chloroquine and confirmed that USP14

inhibition resulted in increased autophagic flux in SH-SY5Y cells (for reviewer's consideration Fig 4). Co-localisation studies by confocal microscopy also revealed increased global and punctate LC3 signal following USP14 interference, which was also found to be evident by electron microscopy. Reassured by these findings, we proceeded by investigating mitophagy, which we extensively did by using multiple different assays.

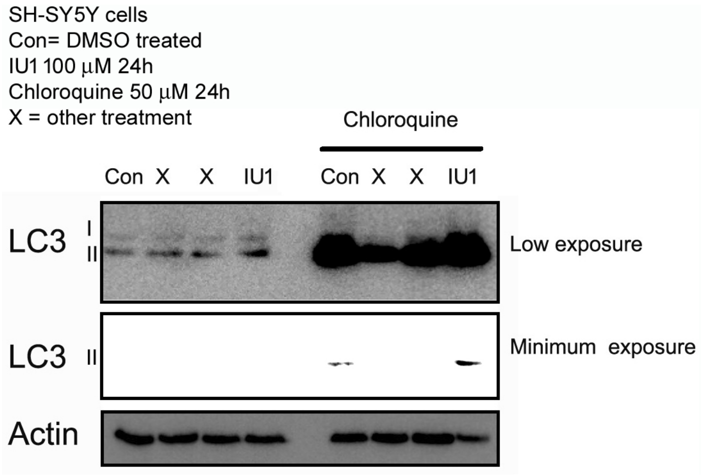

With respect to the EM data*,* we agree with the reviewer that it might be difficult to clearly discriminate between autophagosomes or autolysosomes (or somewhere in between). For this reason, and in accordance with the reviewer, we changed the term "autophagosome" to "autophagosome+autolysosome" per cell in the figures and in the main text. That does not change the rational behind that experiment, which had the intention of detecting mitochondria-like structures inside these vesicles. It should also be noticed that the analysis and detection was done blind folded, by the experienced technical assistants of the institute's electron microscopy facility.

It is not clear why the authors chose to include data with the inhibitor IU1 in the main text and delegate the data with USP14 siRNA to the supplemental figures. Genetic data are arguably more important than pharmacological data for USP14 inhibition.

We agree with the reviewer that genetic manipulation is probably the best approach to clearly dissect molecular pathways, and in this sense might be considered more important. However, the original intention of the current study was to evaluate the therapeutic medical potential of pharmacological substances that could impact mitophagy and therefore potentially ameliorate PD symptoms. In this respect, we really wanted to emphasize the therapeutic value of USP14 inhibition first, but also address the specificity of our findings by genetic knock down.

The experiment in Figure 2C-D is difficult to interpret, as mitochondrial volume appears to significantly decreased in the Drp1 KO, Mfn1 KO and Mfn2 KO MEFs compared to WT MEFs in the absence of IU1 treatment. These data do not convincingly prove the point that mitochondria need to fragment before undergoing mitophagy.

The point that mitochondria need to fragment before mitophagy has been well documented (Twig et al, 2008), and reproduced by many independent studies / groups. Accordingly, elongated mitochondria are spared from mitophagy (Gomes et al, 2011), which further sustain the knowledge that size matters when it comes to mitophagy. Our aim here was to show that also in IU1-activated mitophagy, an efficient fission machinery needs to be in place for this to occurs, possibly for attaining the optimal size for clearance. The differences between WT and DRP1 or Mfn1/2 KO groups could be because of the knock-out background itself, which affects mitochondrial biogenesis, degradation and orientation. This is expected and hard to avoid. It is for this reason that we did not rely on a single assay (i.e. measurement of mitochondrial volume) to quantify mitochondrial content and assess effect of IU1in different backgrounds. We also measured protein content of mitochondrial matrix resident protein HSP60 and inner membrane resident protein ATP5a (Figure 2E-F). We believe that the corroboration of these data, which basically complements each other, indicates that IU1 is ineffective in the absence of DRP1 and Mfn2.

It is unclear whether the immunogold staining for the UPS 20S subunit is specific in Fig. 4C and S7C, as there appears to be immunogold particles in the interior of the intact mitochondrion shown in the lower left inset of scramble siRNA-treated cells in Fig. S7C. Although the EM findings are included primarily as corroboration of the light microscopic findings, these EM experiments need to be performed in a rigorous manner with extensive controls if they are to be included in the manuscript. The data currently suggests, if taken at face value (Fig. 7C) that in baseline conditions, the UPS 20S proteasome complex is located in the middle of intact mitochondria.

Before performing the final experiments for immunogold staining, we standardised the incubation time for gold enhancer to minimise the noise, keeping two negative controls (one without primary, another without nanogold tagged secondary antibody). As proteasome is quite abandoned, and gold enhancer can amplify even tiny bit of noise, the reaction was carefully optimised (for reviewer's consideration Fig 5). We also carefully optimised the time of enhancer incubation, because gold particles continue to grow with time and if they are big enough we cannot speculate about their precise location. There could be negligible noise or endogenous protein complexes inside or outside mitochondria, which are electron dense and though tiny enough, may appear as small diffused black dots. However, size and intensity of the immunogold positive puncta is quite distinctive as in the case of the provided images. In any case, we apologise if the supplementary figure S7 and 4C are unclear and might lead to the impression that the UPS 20S proteasome complex is located in the middle of intact mitochondria. We have replaced the image in the revised version, please see revised Figure 4C and S7C.

As the reviewer has mentioned, the immunogold staining is just to supplement the confocal imaging data. UPS translocation on mitochondria for mitophagy has previously been confirmed also (Yoshii et al, 2011). It is up to the reviewer's suggestion whether we should exclude this experiment if it does not satisfy the reviewer standards.

Although the authors discuss the data showing lack of toxicity of IU1, can they speculate on potential adverse consequences of USP14 inhibition - both in the context of non-mitophagy-related effects and the potential long-term consequences of the observed increased numbers of mitochondria with membrane rupture.

Our intention is to project this inhibitor as a boost for mitochondrial clearance in mitophagy deficient diseased states. We do not have any evidence yet and it is very preliminary to suggest the adverse effects of IU1 in control subjects. Further studies are warranted to determine the off-target effects of IU1.The inhibitor might be administered in intermittent acute doses, to boost mitophagy transiently and rejuvenate mitochondrial population (if mitochondrial biogenesis pathway is intact). Investigating effects of IU1in mammalian systems could be the next step, before it can go for pre-clinical or clinical trials, which is applicable for any pharmacological substance.

The statement beginning on line 442 is a bit misleading, claiming that this study is the first effort to fully characterize in vivo the biology of a proteasome-associated deubiquitinating enzyme in the context of mitophagy. Perhaps this is technically correct if USP14 is truly a specific "proteasome-associated" DUB and USP30 is a specific mitochondrial deubiquitinating enzyme. Nonetheless, the authors should acknowledge and discuss their findings in light of previous work such as the Bingol et al, Nature, 2014 study showing that inhibition of USP30 exerts similar beneficial effects on mitophagy and other outcomes in fly models of Parkinson's disease.

We have acknowledged the findings by Bingol et al., 2014 in the revised MS.

Minor Comments:
The manuscript is well-written but needs professional editing throughout for proper English language writing.

We improved the language in the new version. Please see the revised version. To make the revision process as smooth as possible, we highlighted changes in red in the revised manuscript (deletions are in red font plus strike).

Figure S1A-B. The y-axis label is unclear. Fold-change of what? Also, MTT is not an acceptable readout of viability, as it measures cell proliferation and mitochondrial metabolism. This is a

particular issue for a study focused on the use of the IU1 inhibitor to study effects on mitophagy. Net, cell viability should be assessed using a direct measure of cell survival that is independent of these other variables.

We apologise for the confusion. This depicts difference in the fold change compared to the value where cells were incubated with equal volume of the vehicle. Actually we used this assay to follow the report that discovered IU1. They used this assay for MEF cells. However, we supplemented the data with new experiments (by Propidium Iodide / Hoechst staining) that shows IU1 is not toxic up to 100 mM concentration (please see revised Figure S1).

Figure S1C. For non-experts in Drosophila wing motor neuron morphology, these images are difficult to interpret. The IU1 100 micromolar image looks different than the others, but I do not know whether this is biologically real/meaningful or not. Is there are a more objective or quantitative method to assess whether this concentration of IU1 has effects on Drosophila wing motor morphology?

Neurotoxins affect neurons without showing any adverse effect on other body parts initially. Generally the wing morphology also changes later when wing neurons are damaged, which was not the case here (in any of the IU1 doses). *Drosophila* wing motor neuron imaging has emerged as a very effective tool to monitor the neuron in live animals to screen neurotoxicity, which is quite problematic for the other animal systems.

When neuropathy occurs in the neurons, it appears bead like or obvious bulge like structures. This is quite obvious and quantitative measurements can be taken, which is not the case here. We did not observe any significant change in the 100 mM dose group. Though the flies are from the same clonal lines, there is always a little variation in the gross morphology of motor neurons, which is expected.

Figure S2A-B. The authors do not clearly explain the rationale for measuring chymotrypsin-like activity. More importantly, the effects of IU1 on this activity are not that striking. As the authors acknowledge the caveats of this assay to measure proteasome activity (lines 125-128) and then go on to use alternative assays, I think the data in Figure S1A-B detracts from the study and its removal from the manuscript could be considered.

We thank the reviewer for this suggestion. We agree with the review that the removal of these data from the manuscript does not affect the take home message. We have therefore removed this data from the revised MS.

Figure S2C-D. The figure would be clearer if the authors change "GFP" to "GFP-Ub."

We have changed GFP to GFP-Ub.

Figure 1E. Although there is quantitation provided normalizing the mitochondrial protein levels to actin, it is notable that there is a significant decrease in the Actin loaded on the gel beneath ATP5a in the IU1-treated group. This uneven loading for actin raises concerns about the validity of the conclusion that ATP5a levels are decreased.

We have changed the representative blot.

Figure S3. The authors need to show western blots or RT-PCR to confirm that the USP14 siRNA is truly knocking down USP14.

We have carefully standardised the half-life and mode of degradation of USP14 protein and then studied efficacy of USP14 siRNA treatment by western blot before performing the experiments. We have shown western blot of USP14 after treatment with scr/USP14 siRNA in figure S6.

Figure S4. As above, I think the authors are incorrectly calling autolysosomes (or autophagolysosomes) autophagosomes. The arrowhead in S4B convincingly shows a mitochondria inside an autophagic structure. However, in S4A, the arrowhead does not convincingly show a mitochondrion. The structure delineated by the arrowhead is in too advanced a stage of degradation to discern whether it is or is not a mitochondrion.

We are very grateful to the reviewer for the careful analysis of the images; we have addressed this issue, and as previously mentioned we have changed the term "autophagosome" to "autophagosome+autolysosome" (per cell) in the figures and in the main text (please see revised Fig. S4). We also replaced the image with a better representative one. We would like to point out that mitochondrial cristae structure changes quickly inside autophagosome, mostly because of mitochondrial membrane depolarisation (so most of the time it appears white). We detected a mitochondrion inside autolysosome/autophagosome based on three primary criteria: the size, the presence of a few numbers of cristae-like structures, and presence of double membrane structure at least in some parts.

Figure S5A. This figure is not interpretable. The authors need to assess LC3 (and preferably also p62) in cell treated with control or IU1 in the presence or absence of IU1 in the same experiment. i.e. while there is more LC3-II in the IU1-treated cells than the control cells, it is not possible to determine whether there is an increase in LC3-II upon chloroquine and IU1 treatment versus IU1 treatment alone. This speaks to the central question raised above of whether the data convincingly show that IU1 increases autophagic flux (or rather, blocks, autophagosomal maturation). Also, for HSP60 and ATP5a levels, it is important to compare no chloroquine treatment and chloroquine treatment conditions in the same experiment (gel).

As previously mentioned, it has been shown by independent studies that USP14 inhibition has direct effect on LC3/ p62 levels and autophagic flux. Here our main aim is to prove that when autophagy is obstructed (both pharmacologically and genetically), IU1 mediated decrease in mitochondrial volume is also blocked. We have previously shown that IU1 reduces HSP60 and ATP5a levels, and here we are showing that with chloroquine this effect vanishes even though IU1+Chloroquine treatment has increased LC3 levels. To further prove the point that IU1 mediated decrease in mitochondrial content depends on autophagic machinery, we repeated the experiment in ATG7 KO cells.

We have changed the figure legend. We apologise if the reviewer thinks that chloroquine data is confusing for the readers and it is up to the reviewer's suggestion whether we should exclude the chloroquine part or not.

Lines 178-179. It is unclear what the authors mean by "mitochondrial shape and size are primary prerequisites for mitophagy";'

Mitochondria need to fragment before mitophagy (Twig et al, 2008). Also, elongated mitochondria are spared from mitophagy (Gomes et al, 2011), perhaps because of the hindrance caused during engulfment by autophagic membranes. Smaller, fragmented mitochondria are ideal for clearance. What we meant is that mitochondria size matters when it comes to mitophagy. We apologise if this was not clear in the text. We changed the text from "mitochondrial shape and size are primary prerequisites for mitophagy" to "because mitochondrial shape and size can affect mitophagy (fragmented ones are preferred over the elongated ones), we next evaluated…."

Figure S2B. The results are graphed as "normalized by actin". However, it is unclear what a value of 1.0 means, as the control levels for each protein are not 1.0. This type of "normalization" is unclear and not standard.

We did not find the graph mentioned in figure S2B. We assume that the reviewer meant figure S6B and D. The bar graphs presented are the average of the respective protein band intensity divided by loading control (actin). We have changed the legend in the revised figure.

Figure S6A. The gels for TOM20 and OPA1 are so dark that it is hard to see specific bands. Better quality gels are required.

We have provided with the better quality blots. Please see revised manuscript.

Figure S8C. In the gel shown, it appears that HSP60 (but not ATP55a) is reduced in the PHB2 flox/flox + cre group versus the PHB2 flox/flox group. There are no statistical analyses of this comparison in the graph in Fig. D to see if this is significant, but the data in the gel seem

inconsistent with PHB2 playing a role in mitophagy and make it hard to assess whether PHB2 is required for IU1-induced mitophagy, as the levels of HSP60 already appear lower in the PHB2 deleted cells without IU1 treatment. Thus, the lack of a further decrease with IU1 treatment are difficult to interpret.

It has been shown by previous studies that knocking down PhB2 can fragment mitochondria, and the disruption of PhB2/1 complex may directly affect mitochondrial biogenesis (Merkwirth et al, 2008; Merkwirth & Langer, 2009). We assumed that this could be the reason for the high variations in HSP60 level. Anyways, for this reason we always relied on quantifying two independent proteins to measure mitochondrial content. Our aim here was to assess whether IU1 can further deplete HSP60/ATP5a in PhB2 KO cells. However, to reduce confusion we have replaced the blots with better representative ones.

Figure S10A. The precise p-value calculated by a log-rank test should be provided for these survival analyses. Similarly, statistics are missing for Figure S10B-C.

We have provided the p-value in figure legends and mentioned the statistical differences in Figure S10B-C.

Figure 6A. The red colour of the lines for the Pink1 KO and the Pink1 KO/USP14 KD appear quite similar. The colour of one of these lines should be changed so the reader can more easily appreciate what genotype corresponds to what survival curve. The same concern applies to Figure 7A.7

We have changed the colour of the lines to green.

Figure 6E. I am not sure "dark mitochondria" and "white mitochondria" are standard terms in the field to describe the different mitochondrial morphological phenotypes.

We actually agree with the reviewer and apologies for having used this term. We have changed “dark mitochondria” to electron dense mitochondria and “white mitochondria” to less electron dense mitochondria.

Additional References:
Boselli M, Lee BH, Robert J, Prado MA, Min SW, Cheng C, Silva MC, Seong C, Elsasser S, Hatle KM, Gahman TC, Gygi SP, Haggarty SJ, Gan L, King RW, Finley D (2017) An inhibitor of the proteasomal deubiquitinating enzyme USP14 induces tau elimination in cultured neurons. *J Biol Chem* **292:** 19209-19225

Gomes LC, Di Benedetto G, Scorrano L (2011) During autophagy mitochondria elongate, are spared from degradation and sustain cell viability. *Nat Cell Biol* **13:** 589-598

Merkwirth C, Dargazanli S, Tatsuta T, Geimer S, Lower B, Wunderlich FT, von Kleist-Retzow JC, Waisman A, Westermann B, Langer T (2008) Prohibitins control cell proliferation and apoptosis by regulating OPA1-dependent cristae morphogenesis in mitochondria. *Genes Dev* **22:** 476-488

Merkwirth C, Langer T (2009) Prohibitin function within mitochondria: essential roles for cell proliferation and cristae morphogenesis. *Biochim Biophys Acta* **1793:** 27-32

Twig G, Elorza A, Molina AJ, Mohamed H, Wikstrom JD, Walzer G, Stiles L, Haigh SE, Katz S, Las G, Alroy J, Wu M, Py BF, Yuan J, Deeney JT, Corkey BE, Shirihai OS (2008) Fission and selective fusion govern mitochondrial segregation and elimination by autophagy. *EMBO J* **27:** 433-446

Wei Y, Chiang WC, Sumpter R, Jr., Mishra P, Levine B (2017) Prohibitin 2 Is an Inner Mitochondrial Membrane Mitophagy Receptor. *Cell* **168:** 224-238 e210

Xu D, Shan B, Sun H, Xiao J, Zhu K, Xie X, Li X, Liang W, Lu X, Qian L, Yuan J (2016) USP14 regulates autophagy by suppressing K63 ubiquitination of Beclin 1. *Genes Dev* **30:** 1718-1730

© European Molecular Biology Organization

Yoshii SR, Kishi C, Ishihara N, Mizushima N (2011) Parkin mediates proteasome-dependent protein degradation and rupture of the outer mitochondrial membrane. *J Biol Chem* **286:** 19630-19640

2nd Editorial Decision 11 July 2018

Thank you for the submission of your manuscript to EMBO Molecular Medicine. We have now heard back from the referee whom was asked to evaluate your manuscript.

You will see that while overall supportive, this referee still requests that 2 experiments that were previously requested but not performed, be so as critical for acceptance.

As you may know, we normally entertain one round of main revision. However, as these 2 key experiments were asked for before, and given the supportive reviews otherwise, we would like to give you a last opportunity to address these in a satisfactory manner. I would like to ask you to return the manuscript to us as soon as possible upon completion. In order to gain time, shall the manuscript move forward, please also carefully check our editorial requirements (see below).

I look forward to seeing a revised form of your manuscript as soon as possible.

***** Reviewer's comments *****

Referee #2 (Remarks for Author):

This is a timely and interesting paper that convincingly demonstrates a role for pharmacological and genetic inhibition of the DUB, USP14, in enhancing mitophagy in a PINK1- and Parkin-independent manner. Importantly, USP14 inhibition can reverse abnormal phenotypes in Pink1 or Parkin-deficient flies. Thus, this manuscript both has implications for understanding the molecular mechanisms of mitophagy as well as potentially for defining therapeutic targets for the treatment of Parkinson's disease.

Both referees appreciated the importance of the work during initial review. Referee #1 raised some conceptual questions that the authors partly discussed in their revised text. Referee #2 raised several technical concerns as well as a general concern about the quality of the English language writing. In response, (1) the manuscript has been extensively edited and the writing is now excellent; (2) the authors substituted some images and gels that referees had concerns about with better quality versions; (3) the authors clarified some additional experimental details; and (4) the authors provided detailed explanations in their authors' response letter about remaining issues that they did not address experimentally.

Net, the manuscript is significantly improved by the text changes and some changes in the display items. However, the lack of new experiments to address referee #2's concerns about the strength of the evidence in support of their claim that USP14 inhibition increases autophagy in SH-SY5Y remains a concern. Despite the overall strength and interest of the story, this concern (#2 below), as well as the concern about the chloroquine experiment in Fig. S5A (#4 below), should be addressed prior to publication. While this referee recognizes that these points may not be germane to the central new findings of the manuscript, all conclusions within the manuscript should be supported by appropriately designed experiments.

1. Referee #1 asked for an additional blot in Figure 2E, one for DRP1. This was not provided in the revised manuscript.

2. The authors were asked during original review to assess whether IUI and USP14 knockdown increased autophagic flux. They ascertain in their response that such experiments are not necessary as USP14 has been previously shown to function as a negative regulator of autophagy through a Beclin-dependent pathway and the question of autophagy regulation is not the focus of the paper. I agree with the authors that one does not need to necessarily confirm background literature. However, I do think that any claims made in the present manuscript need to be supported by data that meets the standards of the field. On lines 144-145, the authors state "we confirmed that USP14

inhibition can increase autophagy in SH-SY5Y cells". The current data do NOT support the authors' claim on line 144-145. As previously noted by this referee, the data in the manuscript cannot be interpreted with respect to the question of whether USP14 inhibition truly increases autophagy i.e. whether it increases autophagic flux. They show increased levels of LC3-II by western blot and increased total levels of autophagic structures by electron microscopy. Neither of these assays differentiates increased autophagic flux from a block in autophagosomal maturation. The authors are referred to the guidelines paper in the journal Autophagy (PMID: 2679965) for options of how to correctly assess autophagic flux. Some of the options, such as the performance of western blots to detect proteins such as LC3 and p62 in the presence and absence of a lysosomal inhibitor are quite straightforward and can be performed relatively quickly.

3. Figure S3. Why is there no assessment of HSP60 levels in the USP14 knockdown cells?

4. Figure S5A-B. The experiment in S5B showing that ATG7 KO decreases IUI-induced decreases in ATP5a and HSP60 is an excellent experiment and clearly indicates that the autophagy machinery is required for the effects of IUI on clearance of these mitochondrial proteins (a side point - ideally, a western blot for ATG7 detection should also be included). However, the authors do not provide an adequate response to previous concerns about Figure S5A. In order to draw any conclusions about the effects of chloroquine, either on the autophagy protein LC3 or the mitochondrial degradation of ATP5a or HSP60, they need to have non-chloroquine treated cells treated with vehicle or IUI on the same exact gel. One cannot use historical evidence from other studies to determine whether an "effect vanishes" with chloroquine. One needs to demonstrate in the same experiment that the effect is there without chloroquine before interpreting that it goes away with chloroquine. The authors state that "we apologize if the reviewer thinks that chloroquine data is confusing for the readers and it is up to the reviewer's suggestion whether we should exclude the chloroquine part or not". This reviewer does not think that the chloroquine data are "confusing"; they are uninterpretable as they are lacking essential controls. I think the experiment should be done with essential controls.

Minor Comments:
1. Line 36-37. Sentence is grammatically incorrect and needs editing.
2. Figure S1A. Some points on the graphs are labeled with four asterisks but this is not defined in the legend.
3. Line 140, typographical error "maintained at a low levels"
4. Line 171. Should read "mitochondria-like structures"
5. Line 395. What is "Miro-mitochondrion"?

2nd Revision - authors' response — 04 August 2018

Response to the reviewer's 2 comments.
This is a timely and interesting paper that convincingly demonstrates a role for pharmacological and genetic inhibition of the DUB, USP14, in enhancing mitophagy in a PINK1- and Parkin-independent manner. Importantly, USP14 inhibition can reverse abnormal phenotypes in Pink1 or Parkin-deficient flies. Thus, this manuscript both has implications for understanding the molecular mechanisms of mitophagy as well as potentially for defining therapeutic targets for the treatment of Parkinson's disease.

Both referees appreciated the importance of the work during initial review. Referee #1 raised some conceptual questions that the authors partly discussed in their revised text. Referee #2 raised several technical concerns as well as a general concern about the quality of the English language writing. In response, (1) the manuscript has been extensively edited and the writing is now excellent; (2) the authors substituted some images and gels that referees had concerns about with better quality versions; (3) the authors clarified some additional experimental details; and (4) the authors provided detailed explanations in their authors' response letter about remaining issues that they did not address experimentally.

Net, the manuscript is significantly improved by the text changes and some changes in the display items. However, the lack of new experiments to address referee #2's concerns about the strength of the evidence in support of their claim that USP14 inhibition increases autophagy in SH-SY5Y remains a concern. Despite the overall strength and interest of the story, this concern (#2 below), as

well as the concern about the chloroquine experiment in Fig. S5A (#4 below), should be addressed prior to publication. While this referee recognizes that these points may not be germane to the central new findings of the manuscript, all conclusions within the manuscript should be supported by appropriately designed experiments.

Our response: We thank the reviewer for the appreciation of our study.

1. Referee #1 asked for an additional blot in Figure 2E, one for DRP1. This was not provided in the revised manuscript.

Our response: Actually we changed the blots in the previous response, where we showed that IU1 has no effect on HSP60 and ATP5a levels in DRP1 KO MEF cells, whereas in WT MEF cells IU1 induces the reduction.

2. The authors were asked during original review to assess whether IUI and USP14 knockdown increased autophagic flux. They ascertain in their response that such experiments are not necessary as USP14 has been previously shown to function as a negative regulator of autophagy through a Beclin-dependent pathway and the question of autophagy regulation is not the focus of the paper. I agree with the authors that one does not need to necessarily confirm background literature.

However, I do think that any claims made in the present manuscript need to be supported by data that meets the standards of the field. On lines 144-145, the authors state "we confirmed that USP14 inhibition can increase autophagy in SH-SY5Y cells". The current data do NOT support the authors' claim on line 144-145. As previously noted by this referee, the data in the manuscript cannot be interpreted with respect to the question of whether USP14 inhibition truly increases autophagy i.e. whether it increases autophagic flux. They show increased levels of LC3-II by western blot and increased total levels of autophagic structures by electron microscopy. Neither of these assays differentiates increased autophagic flux from a block in autophagosomal maturation. The authors are referred to the guidelines paper in the journal Autophagy (PMID: 2679965) for options of how to correctly assess autophagic flux. Some of the options, such as the performance of western blots to detect proteins such as LC3 and p62 in the presence and absence of a lysosomal inhibitor are quite straightforward and can be performed relatively quickly.

Our response: As the reviewer has mentioned, these studies are not germane to the central findings, and we thought that the figure provided with the earlier response was satisfactory. However, we appreciate the reviewer's concern and according to the suggestion, we followed "Guidelines for the Use and Interpretation of Assays for Monitoring Autophagy (3rd edition)"(Klionsky et al, 2016). We measured LC3 II levels by immunobloting in presence of IU1 and in combination with two autophagy inhibitors- chloroquine and NH_4Cl (Please see revised Figure S2B). We also counted the degradative and initial autophagic vacuoles by electron microscopy according to the guidelines (Klionsky et al, 2016), and as initially pointed out by this reviewer, and we found an increase in the number of mature autophagic vacuoles (Please see revised Figure S2C).

These evidences indicate that USP14 inhibition leads to increased autophagy. Other autophagy related protein levels can be monitored time and context dependently with different controls, but because they vary cell and time dependently, we believe that investigating those parameters will pull the study exclusively towards an autophagy related report.

3. Figure S3. Why is there no assessment of HSP60 levels in the USP14 knockdown cells?

Our response: We apologise for this mistake. In the new figure we have included HSP60 immunoblot.

4. Figure S5A-B. The experiment in S5B showing that ATG7 KO decreases IUI-induced decreases in ATP5a and HSP60 is an excellent experiment and clearly indicates that the autophagy machinery is required for the effects of IUI on clearance of these mitochondrial proteins (a side point - ideally, a western blot for ATG7 detection should also be included). However, the authors do not provide an adequate response to previous concerns about Figure S5A. In order to draw any conclusions about the effects of chloroquine, either on the autophagy protein LC3 or the mitochondrial degradation of ATP5a or HSP60, they need to have non-chloroquine treated cells treated with vehicle or IUI on the

same exact gel. One cannot use historical evidence from other studies to determine whether an "effect vanishes" with chloroquine. One needs to demonstrate in the same experiment that the effect is there without chloroquine before interpreting that it goes away with chloroquine. The authors state that "we apologize if the reviewer thinks that chloroquine data is confusing for the readers and it is up to the reviewer's suggestion whether we should exclude the chloroquine part or not". This reviewer does not think that the chloroquine data are "confusing"; they are uninterpretable as they are lacking essential controls. I think the experiment should be done with essential controls.

Our response: We thank the reviewer for the comment on ATG7 KO cell experiment. Although we think that this experiment could be self-sufficient to prove that IU1 mediated mitophagy requires the autophagic machinery, we appreciate reviewer's concern and in the revised MS we included all the appropriate controls for S5A. In details we showed that in IU1 treated cells, ATP5a / HSP60 protein levels are deacreased, which cannot be attained when autophagy is inhibited by NH4Cl (Please see revised S5A). According to the guidelines (Klionsky et al, 2016) the compound used to block autophagy must have been confirmed to inhibit autophagy, depending on the cell type and context. We selected NH4Cl because it showed intense increase in LC3 II levels in our hands, as well as in others (Klionsky et al, 2016), for SH-SY5Y. We have also included a blot that shows that the cells are knockout for ATG7, as requested.

Minor Comments:
1. Line 36-37. Sentence is grammatically incorrect and needs editing.

Our response: We have rephrased the sentence.

2. Figure S1A. Some points on the graphs are labeled with four asterisks but this is not defined in the legend.

Our response: we apologise for the mistake. We have corrected that in the revised version.

3. Line 140, typographical error "maintained at a low levels"

Our response: we have corrected the sentence.

4. Line 171. Should read "mitochondria-like structures"

Our response: We have modified the sentence.

5. Line 395. What is "Miro-mitochondrion"?

Our response: We apologise for this. Miro, an element of the primary motor complex for mitochondrial carrier, attaches kinesin to the mitochondrial surface. Its phosphorylation leads to proteasomal degradation. We agree with the reviewer that the sentence does not sound right. We have modified the sentence *"Miro-mitochondrion complex disruption"* to "*Miro degradation, which disrupts its complex with-mitochondrion*".

Reference
Klionsky DJ, Abdelmohsen K, Abe A, Abedin MJ, Abeliovich H, Acevedo Arozena A, Adachi H, Adams CM, Adams PD, Adeli K et al (2016) Guidelines for the use and interpretation of assays for monitoring autophagy (3rd edition). Autophagy 12: 1-222

3rd Editorial Decision 14 August 2018

Thank you for the submission of your revised manuscript to EMBO Molecular Medicine. I am pleased to inform you that we will be able to accept your manuscript pending minor editorial amendments.

EMBO PRESS

YOU MUST COMPLETE ALL CELLS WITH A PINK BACKGROUND ➔

PLEASE NOTE THAT THIS CHECKLIST WILL BE PUBLISHED ALONGSIDE YOUR PAPER

Corresponding Author Name: Elena Ziviani
Journal Submitted to: EMBO Molecular Medicine
Manuscript Number: EMM-2018-09014-V4

Reporting Checklist For Life Sciences Articles (Rev. June 2017)

This checklist is used to ensure good reporting standards and to improve the reproducibility of published results. These guidelines are consistent with the Principles and Guidelines for Reporting Preclinical Research issued by the NIH in 2014. Please follow the journal's authorship guidelines in preparing your manuscript.

A- Figures

1. Data

The data shown in figures should satisfy the following conditions:

- ➔ the data were obtained and processed according to the field's best practice and are presented to reflect the results of the experiments in an accurate and unbiased manner.
- ➔ figure panels include only data points, measurements or observations that can be compared to each other in a scientifically meaningful way.
- ➔ graphs include clearly labeled error bars for independent experiments and sample sizes. Unless justified, error bars should not be shown for technical replicates.
- ➔ if n< 5, the individual data points from each experiment should be plotted and any statistical test employed should be justified
- ➔ Source Data should be included to report the data underlying graphs. Please follow the guidelines set out in the author ship guidelines on Data Presentation.

2. Captions

Each figure caption should contain the following information, for each panel where they are relevant:

- ➔ a specification of the experimental system investigated (eg cell line, species name).
- ➔ the assay(s) and method(s) used to carry out the reported observations and measurements
- ➔ an explicit mention of the biological and chemical entity(ies) that are being measured.
- ➔ an explicit mention of the biological and chemical entity(ies) that are altered/varied/perturbed in a controlled manner.

- ➔ the exact sample size (n) for each experimental group/condition, given as a number, not a range;
- ➔ a description of the sample collection allowing the reader to understand whether the samples represent technical or biological replicates (including how many animals, litters, cultures, etc.).
- ➔ a statement of how many times the experiment shown was independently replicated in the laboratory.
- ➔ definitions of statistical methods and measures:
  - common tests, such as t-test (please specify whether paired vs. unpaired), simple χ2 tests, Wilcoxon and Mann-Whitney tests, can be unambiguously identified by name only, but more complex techniques should be described in the methods section;
  - are tests one-sided or two-sided?
  - are there adjustments for multiple comparisons?
  - exact statistical test results, e.g., P values = x but not P values < x;
  - definition of 'center values' as median or average;
  - definition of error bars as s.d. or s.e.m.

Any descriptions too long for the figure legend should be included in the methods section and/or with the source data.

In the pink boxes below, please ensure that the answers to the following questions are reported in the manuscript itself. Every question should be answered. If the question is not relevant to your research, please write NA (non applicable). We encourage you to include a specific subsection in the methods section for statistics, reagents, animal models and human subjects.

USEFUL LINKS FOR COMPLETING THIS FORM

http://www.antibodypedia.com
http://1degreebio.org
http://www.equator-network.org/reporting-guidelines/improving-bioscience-research-repo
http://grants.nih.gov/grants/olaw/olaw.htm
http://www.mrc.ac.uk/Ourresearch/Ethicsresearchguidance/Useofanimals/index.htm
http://ClinicalTrials.gov
http://www.consort-statement.org
http://www.consort-statement.org/checklists/view/32-consort/66-title
http://www.equator-network.org/reporting-guidelines/reporting-recommendations-for-tun
http://datadryad.org
http://figshare.com
http://www.ncbi.nlm.nih.gov/gap
http://www.ebi.ac.uk/ega
http://biomodels.net/
http://biomodels.net/miriam/
http://jjj.biochem.sun.ac.za
http://oba.od.nih.gov/biosecurity/biosecurity_documents.html
http://www.selectagents.gov/

B- Statistics and general methods

Please fill out these boxes ➔ (Do not worry if you cannot see all your text once you press return)

1.a. How was the sample size chosen to ensure adequate power to detect a pre-specified effect size?	The sample size was chosen to ensure 80% power to detect an effect size of 0.75 on the basis of 5% type I error rate (t-test for two independent sample comparisons, ANOVA and ANOVA ad hoc for multiple comparison)
1.b. For animal studies, include a statement about sample size estimate even if no statistical methods were used.	No statistical method was used to predetermine sample size. We did not use any live vertebrate model in this study.
2. Describe inclusion/exclusion criteria if samples or animals were excluded from the analysis. Were the criteria pre-established?	No samples or animals were excluded from the analysis. We therefore did not use any pre-established criteria to include/exclude samples or animals.
3. Were any steps taken to minimize the effects of subjective bias when allocating animals/samples to treatment (e.g. randomization procedure)? If yes, please describe.	Animals as well as samples were randomly chosen for treatment.
For animal studies, include a statement about randomization even if no randomization was used.	Animals (D. melanogaster) were randomly choosen from the same species.
4.a. Were any steps taken to minimize the effects of subjective bias during group allocation or/and when assessing results (e.g. blinding of the investigator)? If yes please describe.	The investigator was not blinded to allocation during most of the in vitro experiments and outcome assessment. Western blotting analysis of some of the key experiments however were repeated by a second operator who did not know what to expect as outcome assessment.
4.b. For animal studies, include a statement about blinding even if no blinding was done	The investigator that perfomed the experiments in vivo was blinded to allocation during experiments and outcome assessment.
5. For every figure, are statistical tests justified as appropriate?	Yes. Please refer to page 31 for statistical analysis details, and to each figure caption and supplemental table for sample size and p value.
Do the data meet the assumptions of the tests (e.g., normal distribution)? Describe any methods used to assess it.	Statistical tests to evaluate Gaussianity need large sample size, moreover the normal distribution assumption is at the level of population. Given the nature of our measurements we do not have any evidence of deviation from symmetry and curtsies. Furthermore the assumption of measurements independence is always meets
Is there an estimate of variation within each group of data?	The estimation of variation is always included for each group, including the control group. Variation of the control group has always been taken into account to rule out the possibility that the difference between samples to treatment is smaller than the intrinsic variation of the control.
Is the variance similar between the groups that are being statistically compared?	The variance between gruops is similar.

C- Reagents

6. To show that antibodies were profiled for use in the system under study (assay and species), provide a citation, catalog number and/or clone number, supplementary information or reference to an antibody validation profile. e.g., Antibodypedia (see link list at top right), 1DegreeBio (see link list at top right).	We did that for each antibody tht was used in this study.
7. Identify the source of cell lines and report if they were recently authenticated (e.g., by STR profiling) and tested for mycoplasma contamination.	We did that for each cell line that was used in this study.

* for all hyperlinks, please see the table at the top right of the document

D- Animal Models

8. Report species, strain, gender, age of animals and genetic modification status where applicable. Please detail housing and husbandry conditions and the source of animals.	Drosophila melanogaster. Drosophila stocks were maintained under standard conditions at 25C on agar, cornmeal and yeast food. Please refer to Materials and Methods section page 29 for details about specific strains that were used in this study.
9. For experiments involving live vertebrates, include a statement of compliance with ethical regulations and identify the committee(s) approving the experiments.	NA
10. We recommend consulting the ARRIVE guidelines (see link list at top right) (PLoS Biol. 8(6), e1000412, 2010) to ensure that other relevant aspects of animal studies are adequately reported. See author guidelines, under 'Reporting Guidelines'. See also: NIH (see link list at top right) and MRC (see link list at top right) recommendations. Please confirm compliance.	We confirm compliance

E- Human Subjects

11. Identify the committee(s) approving the study protocol.	The study protocol was approved by the ethic committe of Fondazione Ospedale San Camillo IRCCS, Venezia, Italia
12. Include a statement confirming that informed consent was obtained from all subjects and that the experiments conformed to the principles set out in the WMA Declaration of Helsinki and the Department of Health and Human Services Belmont Report.	Informed consent was obtained from all subjects and the experiments conformed to the WMA Declaration of Helsinki and the Department of Health and Human Services Belmont Report.
13. For publication of patient photos, include a statement confirming that consent to publish was obtained.	NA
14. Report any restrictions on the availability (and/or on the use) of human data or samples.	No restrictons on the availability to report.
15. Report the clinical trial registration number (at ClinicalTrials.gov or equivalent), where applicable.	NA
16. For phase II and III randomized controlled trials, please refer to the CONSORT flow diagram (see link list at top right) and submit the CONSORT checklist (see link list at top right) with your submission. See author guidelines, under 'Reporting Guidelines'. Please confirm you have submitted this list.	NA
17. For tumor marker prognostic studies, we recommend that you follow the REMARK reporting guidelines (see link list at top right). See author guidelines, under 'Reporting Guidelines'. Please confirm you have followed these guidelines.	NA

F- Data Accessibility

18: Provide a "Data Availability" section at the end of the Materials & Methods, listing the accession codes for data generated in this study and deposited in a public database (e.g. RNA-Seq data: Gene Expression Omnibus GSE39462, Proteomics data: PRIDE PXD000208 etc.) Please refer to our author guidelines for 'Data Deposition'.<br><br>Data deposition in a public repository is mandatory for:<br>a. Protein, DNA and RNA sequences<br>b. Macromolecular structures<br>c. Crystallographic data for small molecules<br>d. Functional genomics data<br>e. Proteomics and molecular interactions	NA
19. Deposition is strongly recommended for any datasets that are central and integral to the study; please consider the journal's data policy. If no structured public repository exists for a given data type, we encourage the provision of datasets in the manuscript as a Supplementary Document (see author guidelines under 'Expanded View' or in unstructured repositories such as Dryad (see link list at top right) or Figshare (see link list at top right).	NA
20. Access to human clinical and genomic datasets should be provided with as few restrictions as possible while respecting ethical obligations to the patients and relevant medical and legal issues. If practically possible and compatible with the individual consent agreement used in the study, such data should be deposited in one of the major public access-controlled repositories such as dbGAP (see link list at top right) or EGA (see link list at top right).	NA
21. Computational models that are central and integral to a study should be shared without restrictions and provided in a machine-readable form. The relevant accession numbers or links should be provided. When possible, standardized format (SBML, CellML) should be used instead of scripts (e.g. MATLAB). Authors are strongly encouraged to follow the MIRIAM guidelines (see link list at top right) and deposit their model in a public database such as Biomodels (see link list at top right) or JWS Online (see link list at top right). If computer source code is provided with the paper, it should be deposited in a public repository or included in supplementary information.	NA

G- Dual use research of concern

22. Could your study fall under dual use research restrictions? Please check biosecurity documents (see link list at top right) and list of select agents and toxins (APHIS/CDC) (see link list at top right). According to our biosecurity guidelines, provide a statement only if it could.	NA
